# Infiltration metasomatism of the Allende coarse-grained calcium-aluminum-rich inclusions

**DOI:** 10.1186/s40645-021-00437-4

**Published:** 2021-11-04

**Authors:** Alexander N. Krot, Michail I. Petaev, Kazuhide Nagashima

**Affiliations:** 1 Hawai’i Institute of Geophysics and Planetology, School of Ocean and Earth Science and Technology, University of Hawai’i at Mānoa, Honolulu, HI 96822 USA; 2 Department of Earth and Planetary Sciences, Harvard University and Harvard-Smithsonian Center for Astrophysics, Cambridge, MA 02138 USA

## Abstract

We report on the mineralogy, petrography, and O and Al-Mg isotopic systematics of secondary mineralization in the metasomatically altered igneous Ca,Al-rich inclusions (CAIs) [compact type A (CTA), B1, B2, forsterite-bearing B (FoB), and C] from the CV3 carbonaceous chondrite Allende. This alteration affected mainly melilite, and to a lesser degree anorthite, and resulted in the formation of a variety of secondary minerals, including adrianite, Al-diopside, andradite, anorthite, calcite, celsian, clintonite, corundum, dmisteinbergite, ferroan olivine, ferroan monticellite, ferroan Al-diopside, forsterite, grossular, heazlewoodite, hedenbergite, hutcheonite, kushiroite, margarite, monticellite, Na-melilite, nepheline, pentlandite, pyrrhotite, sodalite, spinel, tilleyite, wadalite, and wollastonite. The secondary mineral assemblages are mainly defined by chemical compositions of the primary melilite replaced and elements introduced by an aqueous fluid. Gehlenitic melilite (Åk_<35_) in CTAs and mantles of B1s is mainly replaced by anorthite + grossular; clintonite, corundum, spinel, and Al-diopside are minor. Åkermanitic melilite (Åk_35-90_) in type B2s, FoBs, and cores of B1s is replaced by the grossular + monticellite + wollastonite, grossular + monticellite, and grossular + Al-diopside assemblages; forsterite, spinel, clintonite, and Na-melilite are minor. In type Cs, lacy melilite (åkermanitic melilite with rounded inclusions of anorthite) is pseudomorphically replaced by the grossular + forsterite + monticellite and grossular + Al-diopside assemblages; Na-melilite is minor. Primary and secondary anorthites in the peripheral portions of CAIs are replaced by nepheline, sodalite, and ferromagnesian olivine. Some CAIs contain voids and cracks filled by andradite, hedenbergite, wollastonite, ±sodalite, ±grossular, ±monticellite, ±tilleyite, and ±calcite. All CAIs studied are surrounded by Wark-Lovering rims, fine-grained matrix-like rims composed of lath-shaped ferroan olivine and abundant nepheline grains, and a layer of salite-hedenbergite pyroxenes + andradite + wollastonite. Grossular associating with monticellite, Al-diopside, and forsterite and replacing åkermanitic melilite (^27^Al/^24^Mg ~ 2) has high ^27^Al/^24^Mg ratios (30−100) and shows no resolvable excess of radiogenic ^26^Mg (^26^Mg*). The ^27^Al/^24^Mg ratios (7−10) and ^26^Mg* (2−3‰) in the nearly monomineralic grossular veins crosscutting gehlenitic melilite are similar to those of the host melilite and plot along a regression line with ^26^Al/^27^Al ratio of ~5×10^−5^. Oxygen isotopic compositions of secondary minerals in the most Type Bs measured in situ with the UH Cameca ims-1280 and matrix-matched standards plot along mass-dependent fractionation line with ∆^17^O of ~ −3±2‰ with δ^18^O ranging from ~0 to ~10‰. Primary melilite and anorthite in the host CAIs are similarly ^16^O-depleted, whereas spinel, forsterite, and most Al,Ti-diopside grains have ^16^O-rich compositions (∆^17^O ~ −25±2‰). Secondary grossular and forsterite in type Cs and type B1 CAI *TS-34* show a range of ∆^17^O, from ~ −15 to ~ −1‰; the ^16^O-enriched compositions of grossular and forsterite plot along the carbonaceous chondrite anhydrous mineral line. The similar ranges of ∆^17^O and positions on the three-isotope oxygen diagram are observed for primary anorthite; melilite is generally ^16^O-depleted compared to anorthite (∆^17^O ~ −5 to −1±2‰); spinel and fassaite are ^16^O-rich (except very Ti-rich fassaite in *TS-34* and CTA CAIs)*.* We conclude that Allende CAIs experienced an open-system in situ metasomatic alteration at relatively high temperatures (200-250 °C) in the presence of CO_2_- and H_2_O-bearing fluid with ∆^17^O of ~ −3±2‰ followed by thermal metamorphism at ~ 500 °C on the CV chondrite parent asteroid. During the alteration, most elements were mobile: Si, Na, Cl, K, Fe, S, and Ni were introduced; Al, Ti, Mg, and Ba were locally mobilized; Ca and some Mg and Al were lost from the host inclusions. The alteration occurred after nearly complete decay of ^26^Al, >3 Ma after crystallization of CAIs with the canonical (^26^Al/^27^Al)_0_ of (5.25±0.02)×10^-5^; ^26^Mg* in grossular was inherited from the primary melilite and provide no chronological significance. Oxygen isotopic heterogeneity of primary minerals in the Allende CAIs at least partly is due to isotopic exchange with an aqueous fluid that largely affected melilite, anorthite, perovskite, Zr- and Sc-rich oxides and silicates, and possibly very Ti-rich fassaite.

**Supplementary Information:**

The online version contains supplementary material available at 10.1186/s40645-021-00437-4.

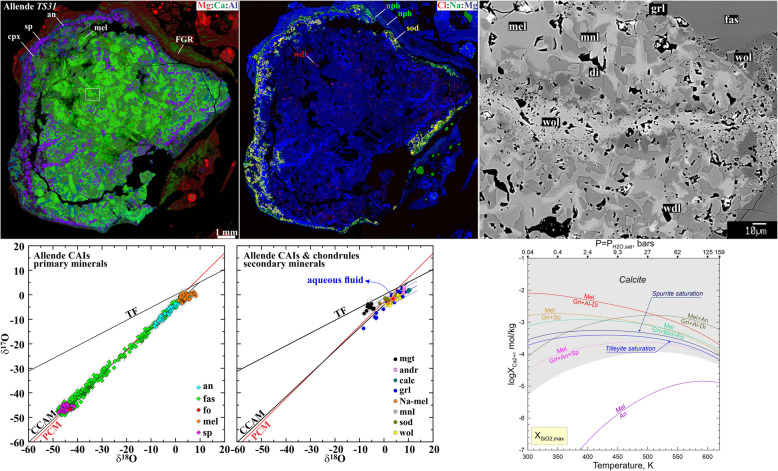

## Introduction

CV (Vigarano type) carbonaceous chondrites experienced metasomatic alteration (fluid-assisted thermal metamorphism) that affected their all major components—refractory inclusions [Ca,Al-rich inclusions (CAIs) and amoeboid olivine aggregates (AOAs)], chondrules, matrix, and chondritic lithic clasts (dark inclusions) (e.g., Kimura and Ikeda 1995; Kojima and Tomeoka 1996; Krot et al. 1995, 1998a, 1998b; Brearley and Krot 2013 and references therein). It is generally accepted that the alteration of the CV chondrules, matrices, and dark inclusions occurred in an asteroidal setting in the presence of aqueous solutions > 3 Ma after crystallization of CAIs having the canonical initial ^26^Al/^27^Al ratio [(^26^Al/^27^Al)_0_] of (5.25±0.02)×10^-5^ (e.g., Larsen et al. 2011; Brearley and Krot 2013 and references therein; Doyle et al. 2015; MacPherson et al. 2017). However, there is no agreement on the timing (early vs. late) and location (nebula vs. asteroid) of alteration of the CV refractory inclusions (e.g., Wark 1981; Hashimoto and Grossman 1987; McGuire and Hashimoto 1989; Fagan et al. 2005, 2006, 2007, 2015; Ushikubo et al. 2007; Krot et al. 2007a, 2007b, 2008; Ford and Brearley 2008), the oldest solids formed in the solar nebula (Bouvier and Wadhwa 2010; Connelly et al. 2012).

Refractory inclusions are thought to have formed near the protoSun in a highly reduced gas of approximately solar composition (H_2_O/H_2_ ~ 6.6×10^−4^; Lodders 2021, which is consistent with an oxidation state of titanium (Ti^3+^/Ti^4+^ ratio) in CAI fassaites (Simon et al. 2007) and the observations that vast majority of CAIs in unmetamorphosed (petrologic type 2-3.0) chondrites have close-to-solar ∆^17^O (deviation from the terrestrial fractionation line, = δ^17^O − 0.52×δ^18^O), ~ -24±2‰ (e.g., Yurimoto et al. 2008; Makide et al. 2009; McKeegan et al. 2011; Bodénan et al. 2014; Kööp et al. 2016; Ushikubo et al. 2017; Krot 2019 and references therein). In contrast, refractory inclusions in the metasomatically altered and metamorphosed CV chondrites of petrologic type ≥ 3.1 have heterogeneous ∆^17^O: spinel, hibonite, forsterite, and most Al,Ti-diopside grains have solar-like ∆^17^O (~ -24±2‰), whereas melilite, anorthite, and, occasionally, Ti-rich pyroxenes are ^16^O-depleted to various degrees (∆^17^O range from ~ -24 to ~ -1‰) (Clayton et al. 1977; MacPherson et al. 2017; Kawasaki et al. 2018). Bulk oxygen-isotope compositions of CAIs and their mineral separates from the CV3.6 chondrite Allende (Bonal et al. 2006) follow a mass-independent fractionation line with a slope of 0.94, named the Carbonaceous Chondrite Anhydrous Mineral (CCAM) line (Clayton et al. 1977). The nature of this oxygen isotopic heterogeneity and the nature of the CCAM line remain controversial. They may reflect gas-melt O-isotope exchange during incomplete melting of coarse-grained igneous CAI precursors in the nebular gas of variable oxygen isotopic composition (e.g., Yurimoto et al. 1998; Kawasaki et al. 2018). Alternatively or in addition to these nebular processes, the CV CAIs could have experienced mineralogically controlled O-isotope exchange during aqueous fluid-rock interaction on the CV chondrite parent asteroid that largely affected their anorthite, melilite, perovskite, grossite, and Zr- and Sc-rich oxides and silicates (e.g., Wakaki et al. 2013; Krot et al. 2008, 2019a, 2019b, 2019c). We note that chondrules and refractory inclusions from the least altered carbonaceous chondrite Acfer 094 (C3.0 ungrouped) have uniform oxygen isotopic compositions and on a three-isotope oxygen diagram plot along the line having a slope of 0.987±0.013, named the Primitive Chondrule Mineral (PCM) line (Ushikubo et al. 2012, 2017), which is different from the CCAM line.

In order to understand the nature (place, time, and mechanism) of metasomatic alteration of the CV CAIs and its possible effect on their O-isotope heterogeneity, we report here on the mineralogy, petrography, and in situ measured O-isotope compositions of secondary minerals in all major types of the Allende coarse-grained igneous CAIs (CTA, B1, B2, FoB, and C; Table 1) using the UH Cameca ims-1280 and matrix-matched standards. For classification of the CV coarse-grained CAIs, see MacPherson (2014) and references therein. Aluminum-magnesium isotope systematics was measured in grossular of several type B1 CAIs.

**Table 1:** A list of coarse-grained igneous CAIs from Allende studied here and compositional ranges of their melilites

CAI	Type	Åk, mol%	Na_2_O, wt%	Ref.
*ALH-2*	FTA/CTA	1−24	n.d.	[1]
*818-G*	CTA	12−27	n.d.	[2]
*CG-13/15*	CTA	3−32	n.d.	[2]
*TS-2*	CTA	9−65	Up to 0.15	[3]
*TS-68*	CTA	8−33	Up to 0.18	[2,4]
*AJEF*	B1	12−61	Up to 0.19	[2]
*Big-All*	B1	n.a.		[2]
*Egg-3*	B1	n.a.		[2]
*TS-23*	B1	10−72	Up to 0.30	[2,6]
*TS-34*	B1	10−65	Up to 0.30	[2,6]
*A-39*	B2	n.a.		[2]
*2*	B2	9−55	Up to 0.17	[2,7]
*3529Z*		B2	n.a.	[2]
*CG-6*	B2	13−75	Up to 0.20	[2]
*CG-10*	B2	10−74	Up to 0.25	[2]
*CG-11*	B2	n.a.	n.a.	[2]
*TS-21*	B2	10−75	Up to 0.30	[2,6]
*TS-31*	B2	76−87	0.10−0.20	[2]
*All-2*	FoB	79−87	0.12−0.23	[2]
*All-5-2*	FoB	66−90		[2,5]
*100*	C	58−74	0.15−0.25	[8,9]
*160*	C	44−72	Up to 0.36	[8,9]

References: [1] Simon et al. (2001); [2] this study; [3] Simon et al. (1998); [4] Simon et al. (1999); [5] Bullock et al. (2012); [6] Simon and Grossman (2006); Kawasaki et al. (2018); [7] Ushikubo et al. (2007); [8] Wark et al. (1987); [9] Krot et al. (2007b)

Preliminary results of this study have been previously reported by Brearley and Krot (2013) and Krot et al. (2010, 2020a).

## Previous studies

The Al-Mg and O isotopic compositions of secondary minerals in Allende CAIs have been previously reported in several papers and abstracts. In this section, we briefly summarize the major results obtained and interpretations proposed in these publications. Secondary minerals, their chemical formulae, and abbreviations used in the paper are listed in Table 2.

**Table 2:** A partial list of secondary minerals in the Allende coarse-grained igneous CAIs, their formulae, and abbreviations used in the text

Mineral/*abbreviation*	Chemical formula
Adrianite	Ca_12_(Al_4_Mg_3_Si_7_)O_32_Cl_6_
Anorthite/*an*	CaAl_2_Si_2_O_8_ (tricl.)
Andradite/*andr*	Ca_3_Fe_2_Si_3_O_12_
Awaruite	Ni_3_Fe
Beckettite	Ca_2_(V,Al,Ti,Mg)_6_Al_6_O_20_
Calcite/*cal*	CaCO_3_
Celsian/*cls*	BaAl_2_Si_2_O_8_
Clintonite/*cln*	Ca(Mg,Al)_3_(Al_3_Si)O_10_(OH)_2_
Corundum/*cor*	Al_2_O_3_
Coulsonite	(Fe,Mg)(V,Al)_2_O_4_
Diopside-Ca-Tschermakite_*ss*_	CaMgSiO_6_-CaAl_2_SiO_6_
Dmisteinbergite/*dms*	CaAl_2_Si_2_O_8_ (hex.)
Forsterite/*fo*	Mg_2_SiO_4_
Grossular/*grs*	Ca_3_Al_2_Si_3_O_12_
Heazlewoodite/*hzw*	Ni_3_S_2_
Hedenbergite/*hed*	CaFeSi_2_O_6_
Hercynite	(Fe,Mg)Al_2_O_4_
Hutcheonite/*htc*	Ca_3_Ti_2_Si_3_O_12_
Ilmenite	FeTiO_3_
Kaolinite	Al_2_Si_2_O_5_(OH)_4_
Magnetite/*mgt*	Fe^2+^Fe^3+^_2_O_4_
Margarite/*mrg*	CaAl_2_(Al_2_Si_2_)O_10_(OH)_2_
Molybdenite	MoS_2_
Monticellite/*mnl*	Ca(Mg,Fe)SiO_4_
Montmorillonite	(Na,Ca)_0.3_(Al,Mg)_2_Si_4_O_10_(OH)•2n(H_2_O)
Na-melilite/*Na-mel*	(CaNa)_2_(Al,Mg)[(AlSi)_2_O_7_]
Nepheline/*nph*	NaAlSiO_4_
Ferromagnesian olivine/*ol*	(Fe,Mg)_2_SiO_4_
Pentlandite/*pnt*	(Fe,Ni)_9_S_8_
Scheelite	Ca(WO_4_)
Sodalite/*sod*	Na_8_Al_6_Si_6_O_24_Cl_2_
Sphene	CaTiSiO_5_
Spinel/*sp*	(Mg,Fe)Al_2_O_4_
Tilleyite/*tlt*	Ca_5_Si_2_O_7_(CO_3_)_2_
Vesuvianite	Ca_10_Mg_2_Al_4_(SiO_4_)_5_(Si_2_O_7_)_2_(OH)_4_
Wadalite/*wad*	Ca_12_Al_10_Si_4_O_32_Cl_6_
Wollastonite/*wol*	CaSiO_3_

Wark (1981) described pockets of secondary alteration minerals enriched in Na, K, Rb, Cl, I, Fe, and Zn, including Fe-bearing grossular, plagioclase (An_90-100_), nepheline, sodalite, Fe-bearing spinel, andradite, and olivine and pyroxenes of variable compositions in several Allende CAIs. Wark (1981) concluded that the alteration affected largely melilite and resulted in formation of grossular and anorthite followed by crystallization of nepheline and sodalite, and diffusion of iron into spinel. Based on the presence of alkali-rich halos around the CAIs, which were interpreted as evidence for outward diffusion of Na and K, Wark (1981) concluded that alteration occurred *in the solar nebula* prior to incorporation of CAIs into the Allende meteorite.

Hutcheon and Newton (1981) described secondary minerals in the Allende Type B1 CAIs *TS-23* and *TS-34*. They showed that secondary grossular and monticellite form narrow veins separating primary melilite and anorthite crystals, and occasionally form a corona completely surrounding large melilite grains; bladed wollastonite crystals fill cavities lined with grossular. Hutcheon and Newton (1981) attributed formation of the grossular−monticellite assemblages to thermal decomposition of melilite at ~ 670 °C *in the solar nebula*:

1 $$ \underset{\kern2.25em \overset{\circ }{\mathrm{a}}\mathrm{kermanite}-\mathrm{gehlenite}\ \mathrm{solid}\ \mathrm{solution}\kern1.75em \mathrm{anorthite}\kern4.5em \mathrm{grossular}\kern6.75em \mathrm{monticellite}}{\mathrm{R}1:3{\mathrm{Ca}}_2{\mathrm{MgSi}}_2{\mathrm{O}}_{7\left(\mathrm{s}\right)}+{\mathrm{Ca}}_2{\mathrm{Al}}_2{\mathrm{Si}\mathrm{O}}_{7\left(\mathrm{s}\right)}+{\mathrm{Ca}\mathrm{Al}}_2{\mathrm{Si}}_2{\mathrm{O}}_{8\left(\mathrm{s}\right)}=2{\mathrm{Ca}}_3{\mathrm{Al}}_2{\mathrm{Si}}_3{\mathrm{O}}_{12\left(\mathrm{s}\right)}+3{\mathrm{Ca}\mathrm{MgSiO}}_{4\left(\mathrm{s}\right)}} $$

No resolvable excess of radiogenic ^26^Mg (^26^Mg*) was found in grossular and sodalite: the upper limits on the initial ^26^Al/^27^Al ratio [(^26^Al/^27^Al)_0_] are < 4.4×10^-6^ and < 1×10^-6^, respectively, suggesting the alteration occurred > 2.4 and > 3.9 Ma after crystallization of the host CAIs characterized by approximately the canonical (^26^Al/^27^Al)_0_ of ~ 4.5×10^-5^.

Tomeoka and Buseck (1982) reported intergrown mica and montmorillonite in a fine-grained CAI from Allende, but proposed no mechanism of their formation.

Hashimoto and Grossman (1987) and McGuire and Hashimoto (1989) described secondary grossular, anorthite, feldspathoids, ilmenite, ferroan olivine, andradite, salite-hedenbergite pyroxenes, and phyllosilicates (a mixture of Na-phlogopite and chlorite or Al-rich serpentine) in AOAs and several coarse- and fine-grained CAIs from Allende. These authors concluded that the Allende refractory inclusions experienced an open-system alteration *in the* cooling *solar nebula* (from ~1000 to ~350 K): Si, Na, K, Fe, Cr, Cl, and H_2_O were introduced, whereas Ca was lost, presumably as gaseous Ca(OH)_2_. Magnesium may have been lost during the replacement of melilite by anhydrous minerals and subsequently added during the formation of phyllosilicates.

Keller and Buseck (1991) described secondary micas, clintonite, and margarite in an Allende CTA CAI. Clintonite crystals, 10-500 nm in size, partially replace grossular in alteration veins crosscutting gehlenitic melilite (Åk_10-20_), whereas margarite occurs as lamellae intergrown with secondary anorthite replacing melilite. Keller and Buseck concluded that the micas formed by hydration reactions below 450 K *in the solar nebula*:

2 $$ \underset{\mathrm{anorthite}}{\mathrm{R}2:2{\mathrm{CaAl}}_2{\mathrm{Si}}_2{\mathrm{O}}_{8\left(\mathrm{s}\right)}+{\mathrm{H}}_2{\mathrm{O}}_{\left(\mathrm{g}\right)}=}\underset{\mathrm{margarite}}{{\mathrm{CaAl}}_2\left({\mathrm{Al}}_2{\mathrm{Si}}_2\right){\mathrm{O}}_{10}{\left(\mathrm{OH}\right)}_{2\left(\mathrm{s}\right)}+{\mathrm{CaO}}_{\left(\mathrm{g}\right)}+2{\mathrm{Si}\mathrm{O}}_{2\left(\mathrm{g}\right)}} $$

3 $$ \underset{\mathrm{grossular}}{\mathrm{R}3:2{\mathrm{Ca}}_3{\mathrm{Al}}_2{\mathrm{Si}}_3{\mathrm{O}}_{12}+}\underset{\mathrm{spinel}}{2{\mathrm{Mg}\mathrm{Al}}_2{\mathrm{O}}_{4\left(\mathrm{g}\right)}+2{\mathrm{Fe}}_{\left(\mathrm{g}\right)}+3{\mathrm{H}}_2{\mathrm{O}}_{\left(\mathrm{g}\right)}=}\underset{\mathrm{clintonite}}{\mathrm{Ca}\left({\mathrm{Mg}}_2\mathrm{Al}\right)\left({\mathrm{Al}}_3\mathrm{Si}\right){\mathrm{O}}_{10}{\left(\mathrm{OH}\right)}_2+}\underset{\mathrm{hercynite}}{2{\mathrm{Fe}\mathrm{Al}}_2{\mathrm{O}}_{4\left(\mathrm{s}\right)}+5{\mathrm{Ca}\mathrm{O}}_{\left(\mathrm{g}\right)}+5{\mathrm{Si}\mathrm{O}}_{2\left(\mathrm{g}\right)}+2{\mathrm{H}}_2\ \left(\mathrm{g}\right)} $$

Davis et al. (1994) reported on the mineralogy, petrography, trace element abundances, and Al-Mg isotope systematics of primary and secondary minerals in *TS-23* and *TS-34*. Bulk chemical compositions, trace element abundances, and textures suggest that alteration is dominated by a reaction of Mg-rich melilite with SiO_2_; a reaction of melilite and anorthite also occurred, as did a loss of Sr and gain of volatiles. Melilite mantles of *TS-23* and *TS-34* are only slightly altered and contain mainly grossular veins, whereas larger patches of secondary grossular, monticellite, and wollastonite occur between partially altered grains of anorthite and åkermanitic melilite (Åk_72-76_) in the CAI cores, suggesting that Mg-rich melilite is more easily altered than gehlenitic melilite; fassaite and spinel show no evidence for alteration. No detectable ^26^Mg* was found in grossular; primary anorthite in altered zones shows evidence for disturbed Al-Mg isotope systematics. The rare earth element patterns of altered zones are similar to those of adjacent late-crystallized melilite. The altered zones are enriched in B, F, Na, K, Cl, and Pb relative to the adjacent primary phases; they are also fairly high in Sr, Ba, and Eu. The altered zones appear to have inherited their trace element abundances from melilite; Sr was lost in varying degrees relative to Ba and Eu. Davis et al. (1994) concluded the alteration occurred *in the solar nebula*.

Hiyagon (1998) reported δ^17^O and δ^18^O with large uncertainties (2σ for δ^17^O and δ^18^O are ~3-4 and 4-9‰, respectively) of secondary grossular (-12 to -4‰ and -6 to +2‰, respectively; ∆^17^O ~ -9 to -5‰) replacing melilite and anorthite, and of wollastonite, andradite, and hedenbergite (-3 to + 4‰ and + 2 to + 10‰; ∆^17^O ~ -4 to -1‰) in cavities and topographic depressions on the surface of several coarse-grained Allende CAIs. No matrix-matched standards were used in this study (San Carlos olivine was used as a standard for all secondary minerals). As matrix effects could not have been properly corrected, only ∆^17^O can be trusted.

Ushikubo et al. (2007) reported ^36^Cl-^36^S, ^26^Al-^26^Mg, and oxygen isotopic systematics of secondary minerals (San Carlos olivine was used as a standard) in the Allende Type B2 CAI *2*. Two secondary mineral domains were identified: “ragged” anorthite-grossular domain in the CAI interior and sodalite-anorthite-nepheline-ferroan olivine domain in its outer part. ∆^17^O of ragged anorthite and grossular (SIMS spots in grossular overlapped with primary fassaite and spinel) range from -17 to -9±6‰. Sodalite and anorthite in the Na-rich domain and primary melilite appear to be slightly ^16^O-depleted (∆^17^O ~ -7±6‰). No resolvable ^26^Mg* [(^26^Al/^27^Al)_0_ < 4.4×10^-7^] is observed in grossular, lath-shaped anorthite, and sodalite. In contrast, the inferred (^26^Al/^27^Al)_0_ in ragged anorthite is (1.2±0.2)×10^-5^. Ushikubo et al. (2007) interpreted these observations as an evidence for multiple alteration events experienced by the CAI, 1.5 and 5.7 Ma after its crystallization.

Fagan et al. (2007) studied several type B1, B2, CTA, and fluffy type A (FTA) CAIs from Allende and identified two textural and mineralogic types (domains) of secondary mineralization: a grossular-rich domain concentrated along melilite grain boundaries in CAI interiors and a feldspathoid-bearing domain confined mostly to CAI margins, just interior to the Wark-Lovering rim sequence. In type B1s, most secondary minerals, and some secondary minerals in other CAI types, show no resolvable ^26^Mg*, suggesting formation > 3 Ma after primary CAI minerals. All but two analyses of secondary minerals from the FTA CAI yield (^26^Al/^27^Al)_0_ ~ (4.9±2.8)×10^-6^, suggesting formation ~ 1.8-3.2 Ma after the primary CAI minerals. One grossular from the Type B2 CAI, and several grossular and feldspar analyses from a CTA CAI, have ^26^Mg* consistent with (^26^A/^27^Al)_0_ ~ 4.5×10^-5^. Fagan et al. (2007) suggested that Allende CAIs experienced a *protrackted, episodic alteration: some alteration occurred during the formation of CAIs; some continued during melting events associated with chondrule formation in the solar nebula and/or on the CV parent asteroid*.

Ford and Brearley (2008) described secondary anorthite, grossular**,** nepheline, and sodalite replacing melilite in a Type A CAI from Allende. The CAI is surrounded by an aureole of Ca,Fe-rich pyroxenes and andradite. Ford and Brearley (2008) concluded that Na, Cl, and Si were introduced into the CAI, whereas Ca was lost during metasomatic alteration. Mass-balance calculations show that amount of Ca lost is sufficient to explain Ca in the Ca,Fe-rich aureole, suggesting that the alteration occurred in situ on the CV parent asteroid. Subsequently, Brearley et al. (2014) described dmisteinbergite associated with disordered biopyriboles replacing melilite in this CAI, indicating the formation of the secondary minerals in the presence of an aqueous fluid.

Che and Brearley (2021) described secondary mineralization in a Type C CAI from Allende. Primary anorthite in this inclusion is pseudomorphically replaced by sodalite, nepheline, and ferroan olivine (Fa_48_); secondary hedenbergite, diopside, and wollastonite occur in nodules. Sodalite is more abundant in the core of the CAI, whereas nepheline is more abundant in its mantle. According to Che and Brearley (2021), the zoned distribution of Na- and Cl-bearing phases may either reflect a two-stage alteration process on the CV parent asteroid or evolution of the fluid composition during the alteration.

## Analytical methods

### Mineralogy and petrology

Polished sections of the Allende CAIs provided by 1David Wark (Australian National University), Gerald Wasserburg (California Institute of Technology), Ian Hutcheon (Lawrence Livermore National Laboratory), and Steven Simon (University of Chicago) were mapped in Mg, Ca, Al, Ti, Na, Cl, Si, S, Ni, and Fe K∝ x-rays using a 5-10 μm electron beam, 15 kV accelerating voltage, 50 nA beam current, 10-20 ms per pixel acquisition time, and resolution of 5-10 μm per pixel with wavelength-dispersive spectrometer detectors with the University of Hawai’i (UH) field-emission electron microprobe JEOL JXA-8500F. Elemental maps in (*i*) Mg, Ca, and Al, and (*ii*) Cl, Na, and Al were combined using an RGB-color scheme (Mg, or Cl—red, Ca or Na—green, and Al—blue). The MgCaAl maps are used to illustrate textures and primary mineralogy of the Allende CAIs studied. In these maps, spinel is purple; melilite and most secondary minerals, which typically replace melilite, are bright-green; Al,Ti-diopside is dark-green; anorthite is dark-blue; and forsterite is red. The ClNaAl maps are used to illustrate distribution of adrianite/wadalite (red), sodalite (yellow), and nepheline (green) inside and outside CAIs. All CAIs were subsequently studied in backscattered electron (BSE) images and analyzed for chemical compositions using the UH JEOL JXA-8500F. Quantitative wavelength-dispersive analyses were obtained at 15 kV with counting times of 30 s for peak and for background measurements for each analysis. Natural minerals were used as standards. Electron probe data were reduced via the modified ZAF correction procedure PAP (Pouchou and Pichoir 1984).

Representative chemical compositions of secondary minerals in the Allende igneous CAIs are listed in Supplementary Material (Table EA1). X-ray elemental maps and backscattered electron (BSE) images of the representative Allende CAIs studied are shown in Figs. 1, 2, 3, 4, 5, 6, 7, 8, 9, 10, 11, and 12.

**Fig. 1:**
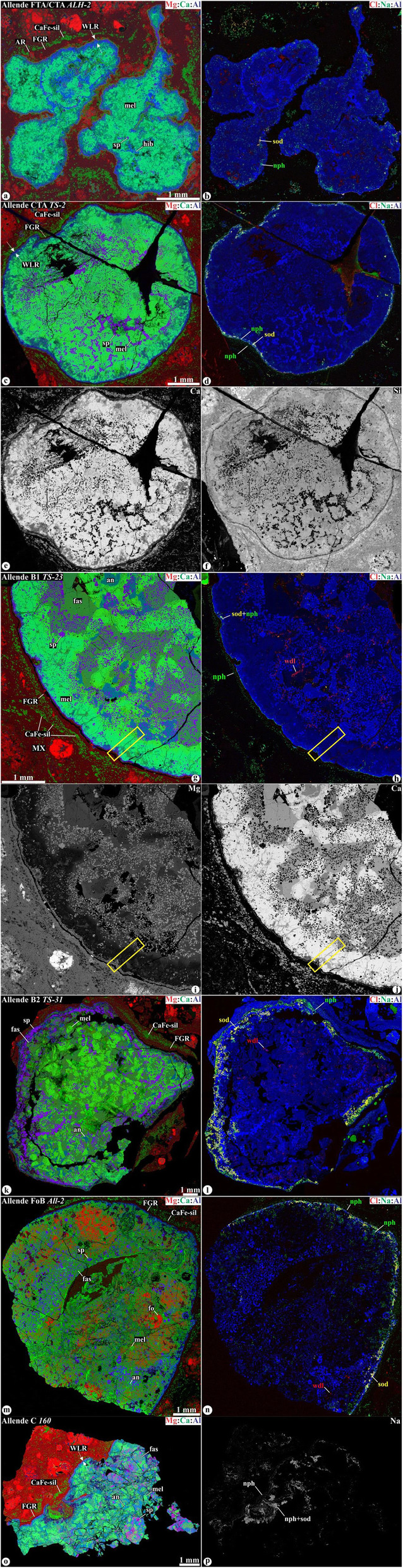
Combined x-ray elemental maps in (**a**, **c**, **g**, **k**, **m**, **o**) Mg (red), Ca (green), and Al (blue), (**b**, **d**, **h**, **l**, **n**) Cl (red), Na (green), and Al (blue) and elemental maps in (**e**, **j**) Ca, (**f**) Si, (**j**) Mg, and (**p**) Na of representative samples of coarse-grained CAIs from the CV3 carbonaceous chondrites Allende studied: *ALH2* (FTA/CTA), *TS2* (CTA), *TS23* (B1), *TS31* (B2), *Al-2* (FoB), and *160* (C). Regions outlined in “**g−j**” are shown in detail in Fig. X. All CAIs are surrounded by Wark-Lovering rims (WLR), fine-grained matrix-like rims (FGR), and a layer of Ca,Fe-rich silicates (CaFe-sil). Nepheline and sodalite are concentrated in the peripheral portions of CAIs. Abundant nepheline grains occur also in fine-grained matrix-like rims. Wadalite/adrianite are found exclusively in cores of Type B CAIs (high Cl signal in **b** comes from epoxy). fas = Al,Ti-diopside; mel = melilite. Hereafter: abbreviations of secondary minerals are listed in Table 1

**Fig. 2:**
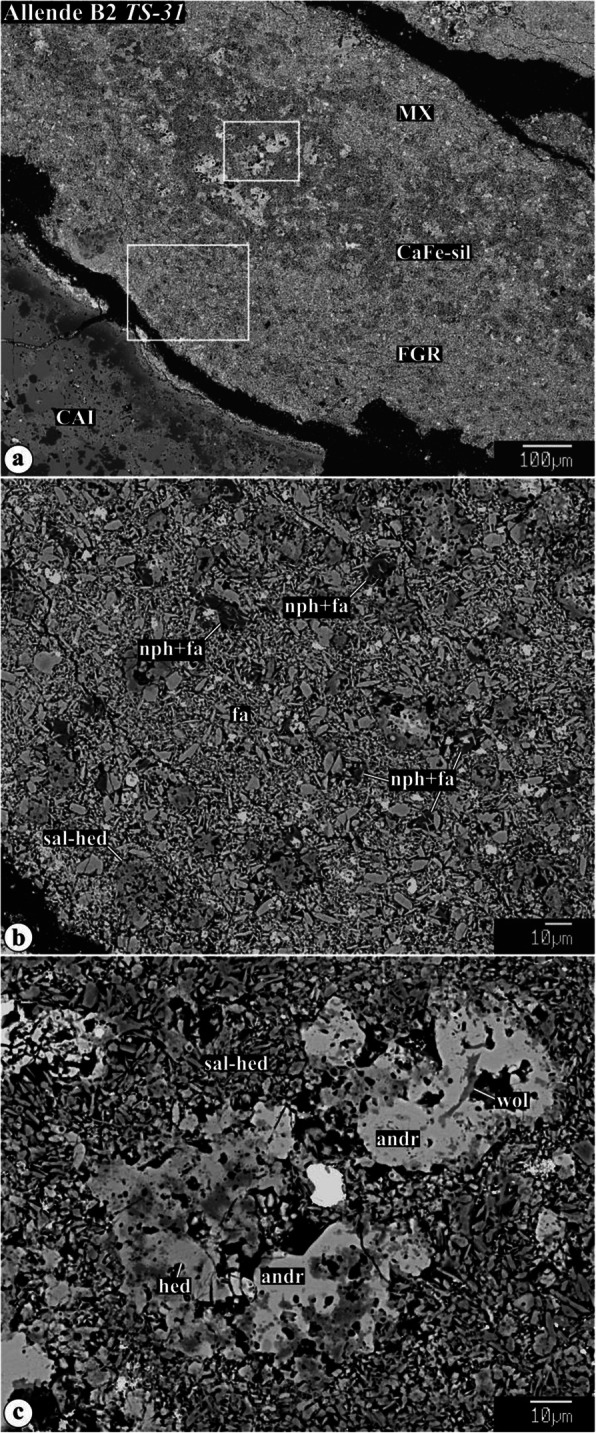
Backscattered electron images of a fine-grained matrix-like rim and a Ca,Fe-rich silicate layer around Type B2 CAI *TS31*. The matrix-like rim consists predominantly of lath-shaped ferroan olivine, nepheline, and salite-hedenbergite pyroxene nodules. The Ca,Fe-rich silicate layer is composed of salite-hedenbergite pyroxenes, andradite, and wollastonite

**Fig. 3:**
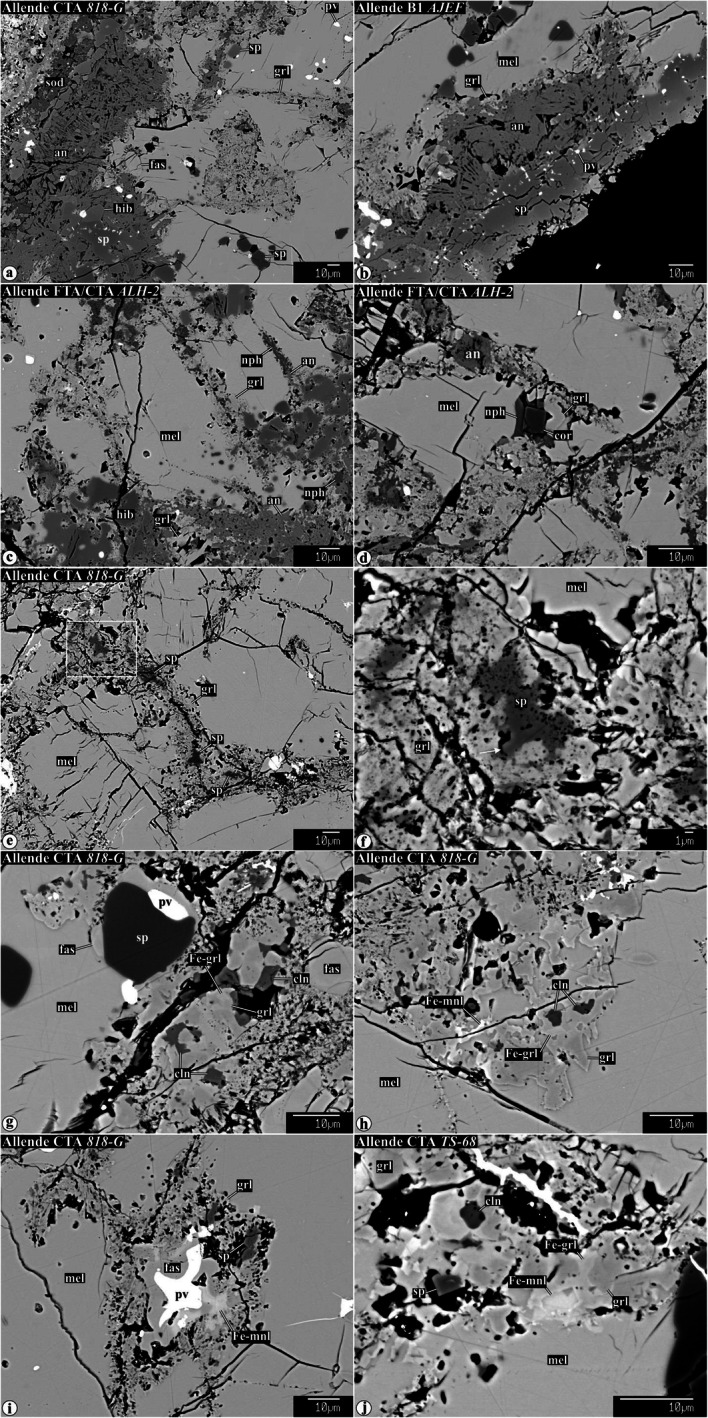
Backscattered electron images of secondary minerals replacing gehlenitic melilite in CTA CAIs and mantles of Type B1 CAIs. In the CAI peripheries, melilite is replaced by anorthite and grossular. The abundance of anorthite decreases, whereas the abundance of grossular increases towards the CAI interiors. Other phases coexisting with grossular in the CAI interiors are micron-sized spinel, clintonite, FeO-enriched monticellite, and FeO-enriched grossular. The ferroan monticellite and grossular overgrow FeO-poor grossular. Clintonite is typically enclosed by grossular. Spinel grains growing into void space in veins have euhedral outlines (indicated by arrow in **f**). Near the CAI edges, secondary anorthite is replaced by nepheline, sodalite, and ferromagnesian olivine

**Fig. 4:**
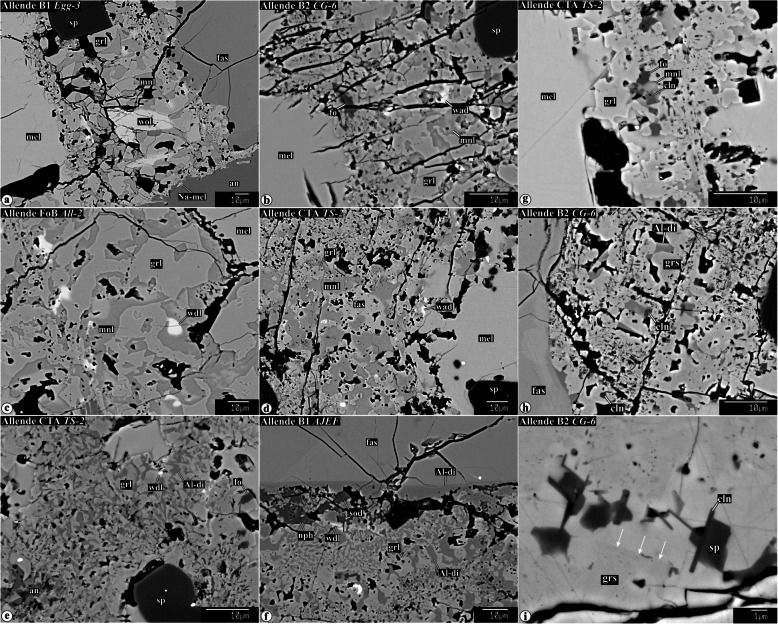
Backscattered electron images of secondary mineral assemblages replacing åkermanitic melilite in Type B1, B2, FoB, and CTA CAIs: **a** grossular-monticellite-wollastonite, **b**−**d** grossular-monticellite, and **e**, **f** grossular-Al-diopside. All these mineral assemblages may contain minor clintonite, forsterite, spinel, Na-melilite, and wadalite. Na-melilite is commonly observed at the boundary between altered melilite and igneous anorthite (see **a**). Arrows in **i** indicate a boundary between grossulars with different MgO contents (dark, 3.7 and light, 2.3 wt%)

**Fig. 5:**
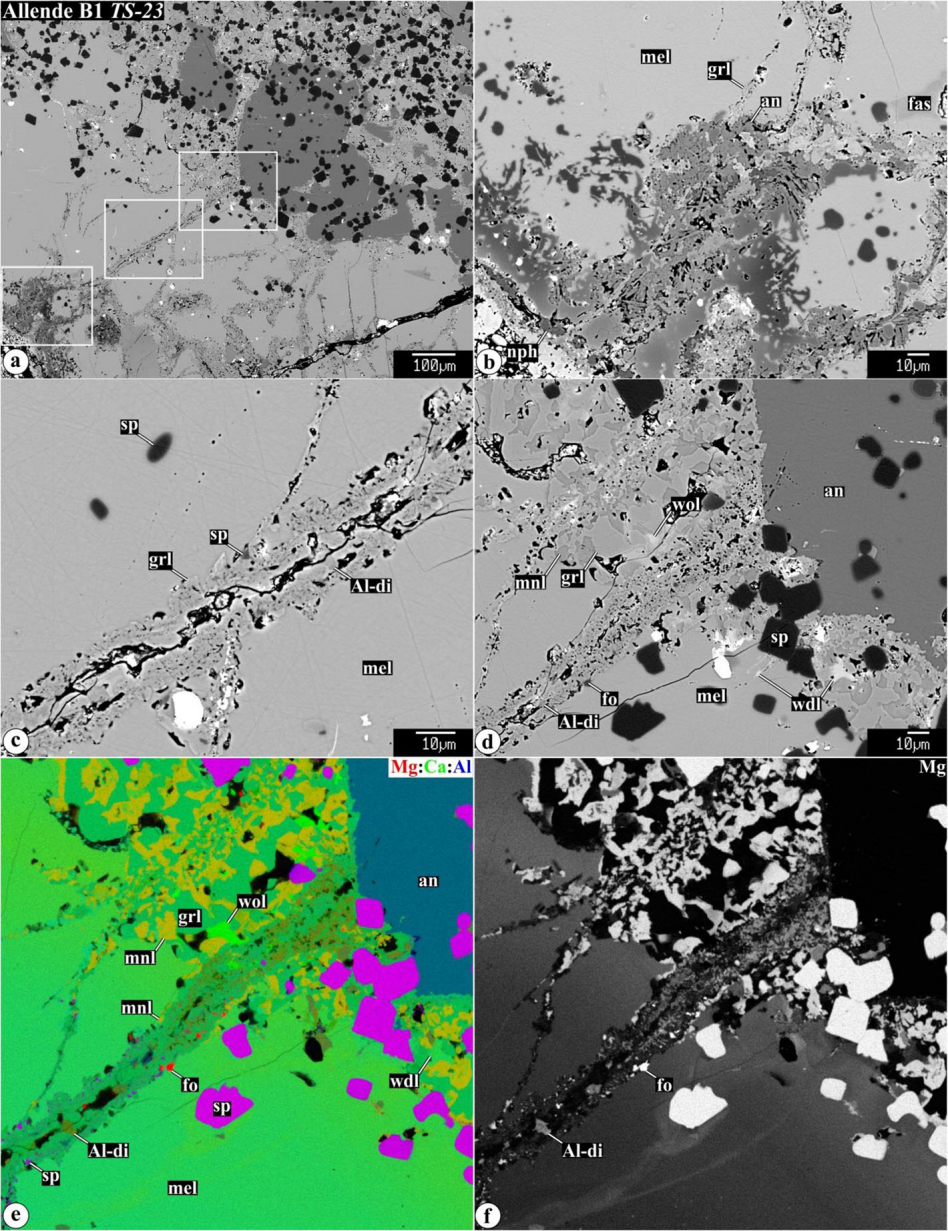
**a**−**d** Backscattered electron images, **e** combined x-ray elemental map in Mg (red), Ca (green), and Al (blue), and **f** elemental map in Mg of a vein crosscutting gehlenitic melilite mantle around Type B1 CAI *TS-23*. Regions outlined in **a** are shown in detail in **b**, **c**, and **d**−**f**, respectively. Towards the CAI interior, the melilite becomes more åkermanitic. The mineralogy of the vein changes towards the CAI interior from anorthite-rich to grossular-rich; minor phases include Al-diopside, forstetrite, and spinel. Åkermanitic melilite in the core is replaced by coarse-grained grossular, monticellite, wollastonite, and minor wadalite

**Fig. 6:**
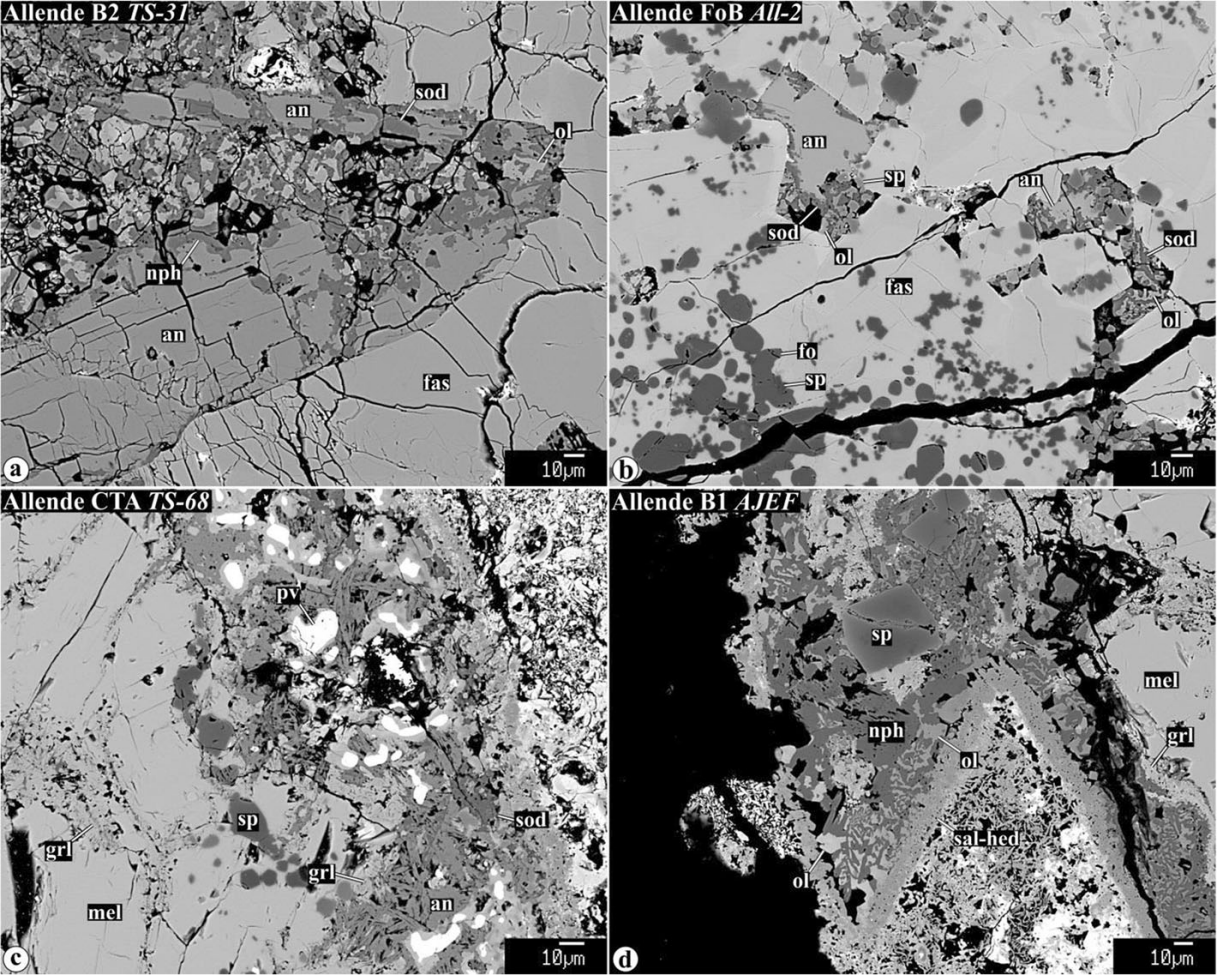
Backscattered electron images primary anorthite (**a**, **b**) and secondary anorthite (**c**, **d**) in the peripheries of the Allende coarse-grained igneous CAIs. Both types of anorthite are replaced by nepheline, sodalite, and ferromagnesian olivine

**Fig. 7:**
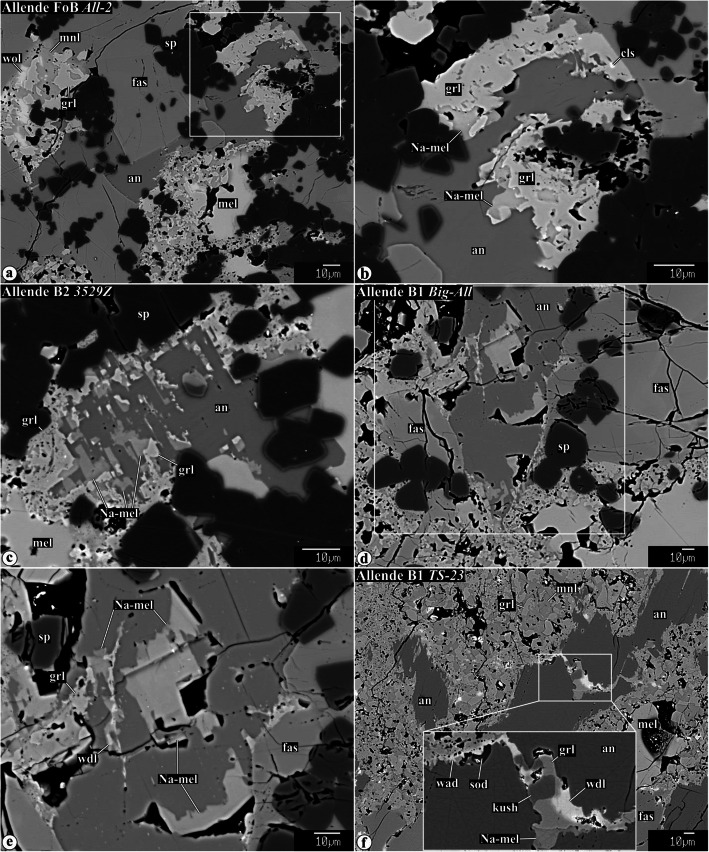
Backscattered electron images of Alteration of primary igneous anorthite in the interior regions of the Type B1, B2, and FoB Allende CAIs. Regions outlined in **a** and **e** are shown in details in **b** and **f**. Anorthite along the boundaries with heavily altered regions of åkermanitic melilite contains irregularly shaped embayments, inclusions along cleavage planes, and veins of Na-melilite, grossular, kushiroite, and wadalite. Occasionally, wadalite is replaced by sodalite. Na-melilite growing into open space shows euhedral outlines (see **e** and **f**). Variations in brightness of Na-melilite correspond to variations in Na_2_O content

**Fig. 8:**
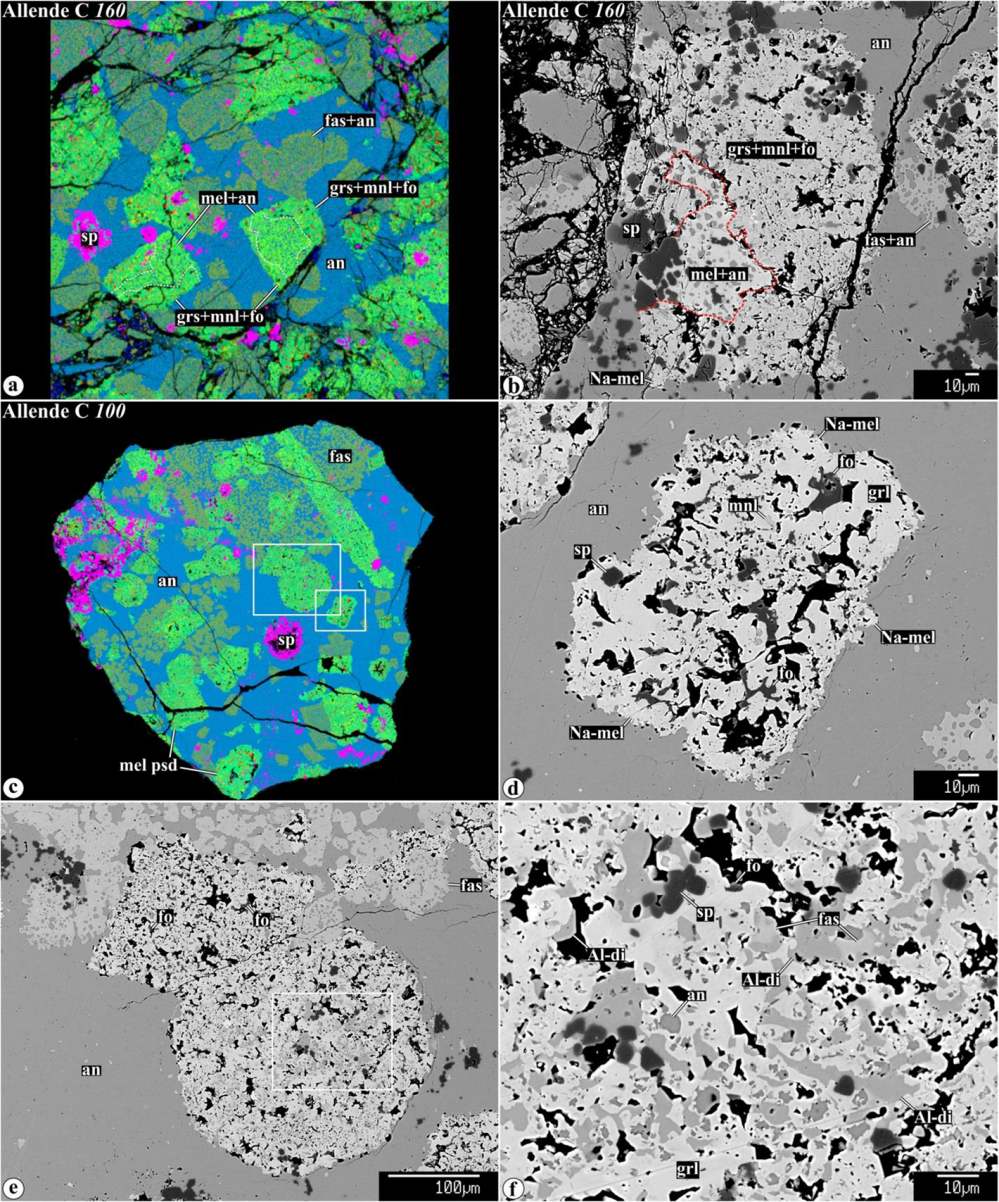
**a**, **c** Combined x-ray elemental maps in Mg (red), Ca (green), and Al (blue), and (**b**, **d**−**f**) backscattered electron images of the interior regions of Type C CAIs (**a**, **b**) *160* and (**c**−**f**) *100.* Regions outlined in **c** and **e** are shown in detail in **d**–**f**, respectively. Lacy melilite composed of åkermanitic melilite with numerous rounded inclusions of anorthite is pseudomorphically replaced to various degrees by grossular-forsterite-monticellite and grossular-Al-diopside. Anorthite groundmass around melilite pseudomorphs shows little evidence for alteration: small amount of Na-melilite occurs along edges of melilite pseudormorphs

**Fig. 9:**
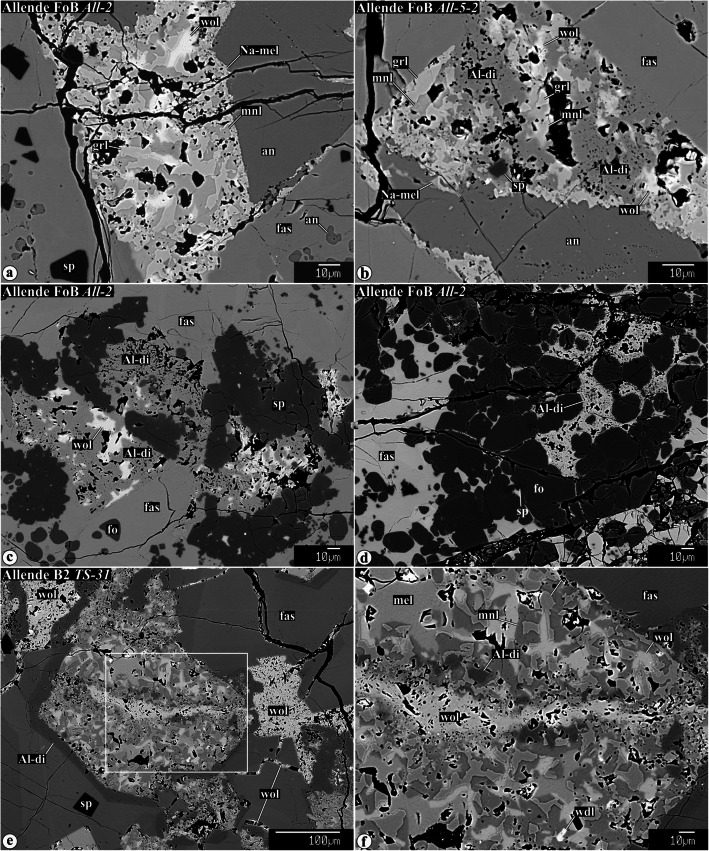
Backscattered electron images of multiple generation of secondary mineral assemblages in the Allende Type B CAIs. Region outlined in **e** is shown in detail in **f**. **a**−**d** Replacement of grossular-monticellite-wollastonite assemblages to various degrees by Al-diopside. **e**, **f** Grossular-monticellite-wollastonite-Al-diopside assemblage is crosscut by a wollastonite vein. Wollastonite also fills veins and irregularly shaped regions in fassaite

**Fig. 10:**
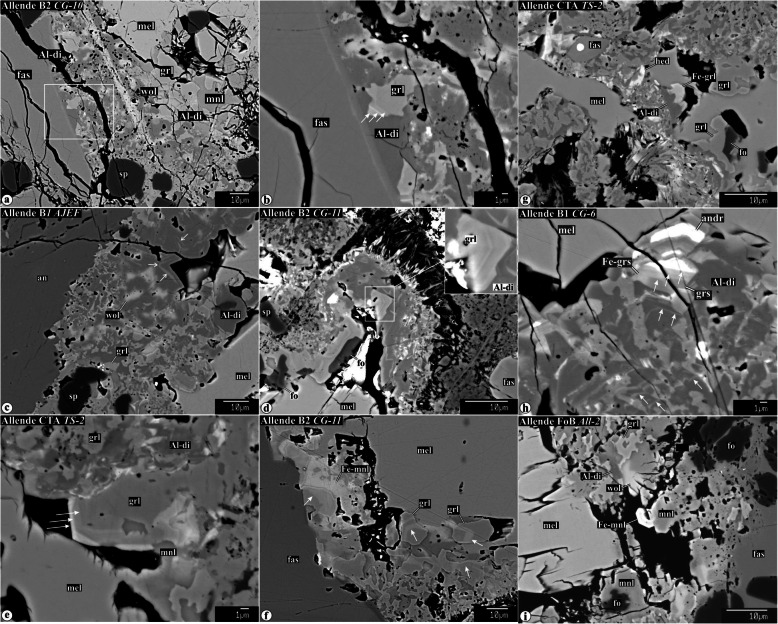
Backscattered electron images of chemically zoned secondary minerals in the Allende coarse-grained CAIs. The oscillatory zoned grossular and Al-diopside are commonly observed in grossular-Al-diopside mineral assemblages and often contain FeO-rich secondary minerals: andradite, hedenbergite, ferroan monticellite, and ferroan grossular

**Fig. 11:**
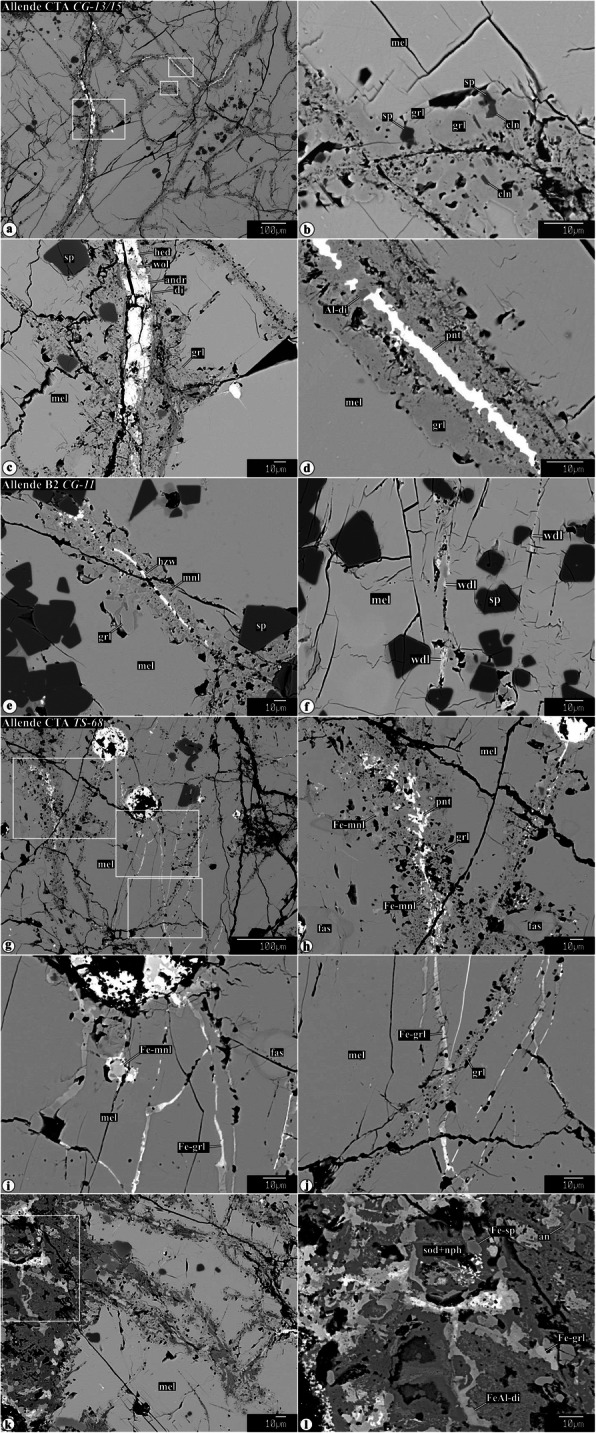
Backscattered electron images of different generations of veins in the Allende coarse-grained CAIs. Regions outlined in **a** and **g** are shown in detail in **b**−**d** and **h**−**j**, respectively. Central regions of some grossular-rich veins are occupied by pentlandite, heazlewoodite, or andradite + wollastonite + hedenbergite. **g**−**l** Occasionally, grossular-rich veins are crosscut by veins composed of ferroan grossular and ferroan-Al-diopside

**Fig. 12:**
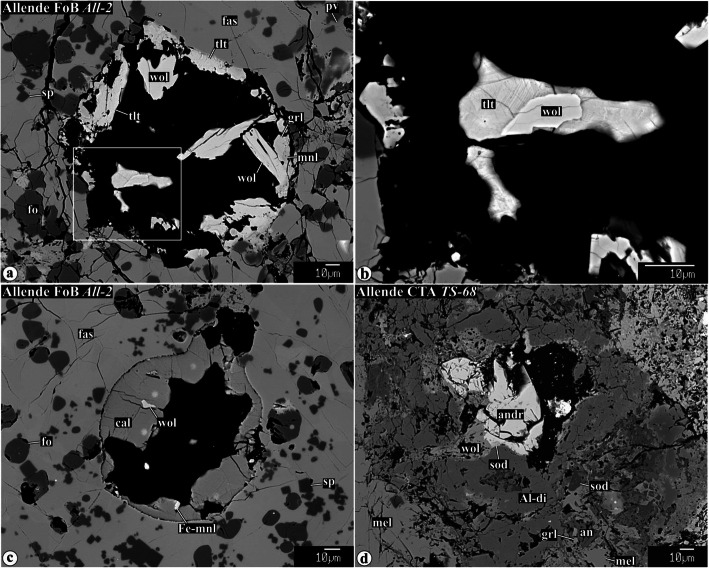
Secondary minerals growing in voids inside coarse-grained Allende CAIs. Region outlined in **a** is shown in detail in **b**

### Oxygen isotopes

Oxygen isotopic compositions were analyzed in situ with the UH Cameca ims-1280 ion microprobe using the method of Nagashima et al. (2015). Briefly, a primary Cs^+^ ion beam of ~25 pA focused to ~1–2 μm was used. The ion microprobe was operated at –10 keV with a 50 eV energy window. Three oxygen isotopes were measured simultaneously: ^16^O^–^ was measured on a Faraday cup (FC) and ^17^O^–^ and ^18^O^–^ were measured on electron multipliers (EMs). The mass resolving power (m/∆m) for ^16^O^–^ and ^18^O^–^ was ~2000, and that for ^17^O^–^ was ~5500, sufficient to separate interfering ^16^OH^–^. A normal-incidence electron flood gun was used for charge compensation. ^16^OH^−^ signal was monitored after each measurement. The contribution of ^16^OH^−^ onto ^17^O^−^ was corrected based on a peak/tail ratio. The correction was typically less than 0.05‰. Instrumental fractionation was corrected using terrestrial standards including San Carlos olivine (for melilite, Na-melilite, forsterite, and ferroan olivine), augite (for Al-diopside and kushiroite), Burma spinel (for spinel), Miyake-jima anorthite (for anorthite and dmisteinbergite), andradite (for andradite), sodalite (for sodalite), nepheline (for nepheline), calcite (for calcite), wollastonite (for wollastonite), and grossular (for grossular) (Nagashima et al. 2020). To verify the positions of the sputtered regions, the spots analyzed for oxygen isotopes were studied with secondary and BSE images using the UH JEOL JXA-8500F electron microprobe before and after SIMS measurements. Oxygen-isotope compositions are reported as δ^17^O and δ^18^O, deviations from Vienna Standard Mean Ocean Water (VSMOW; ^17^O/^16^O_VSMOW_ = 0.000380; ^18^O/^16^O_VSMOW_ = 0.002005; De Laeter et al. 2003) in parts per thousand: δ^17,18^O_SMOW_ = [(^17,18^O/^16^O_sample_) / (^17,18^O/^16^O_VSMOW_) – 1] × 1000, and as deviation from the terrestrial fractionation (TF) line, ∆^17^O. Typical uncertainties for δ^17^O, δ^18^O, and ∆^17^O were ~ 1.5, 2, and 2‰, respectively.

**Fig. 13:**
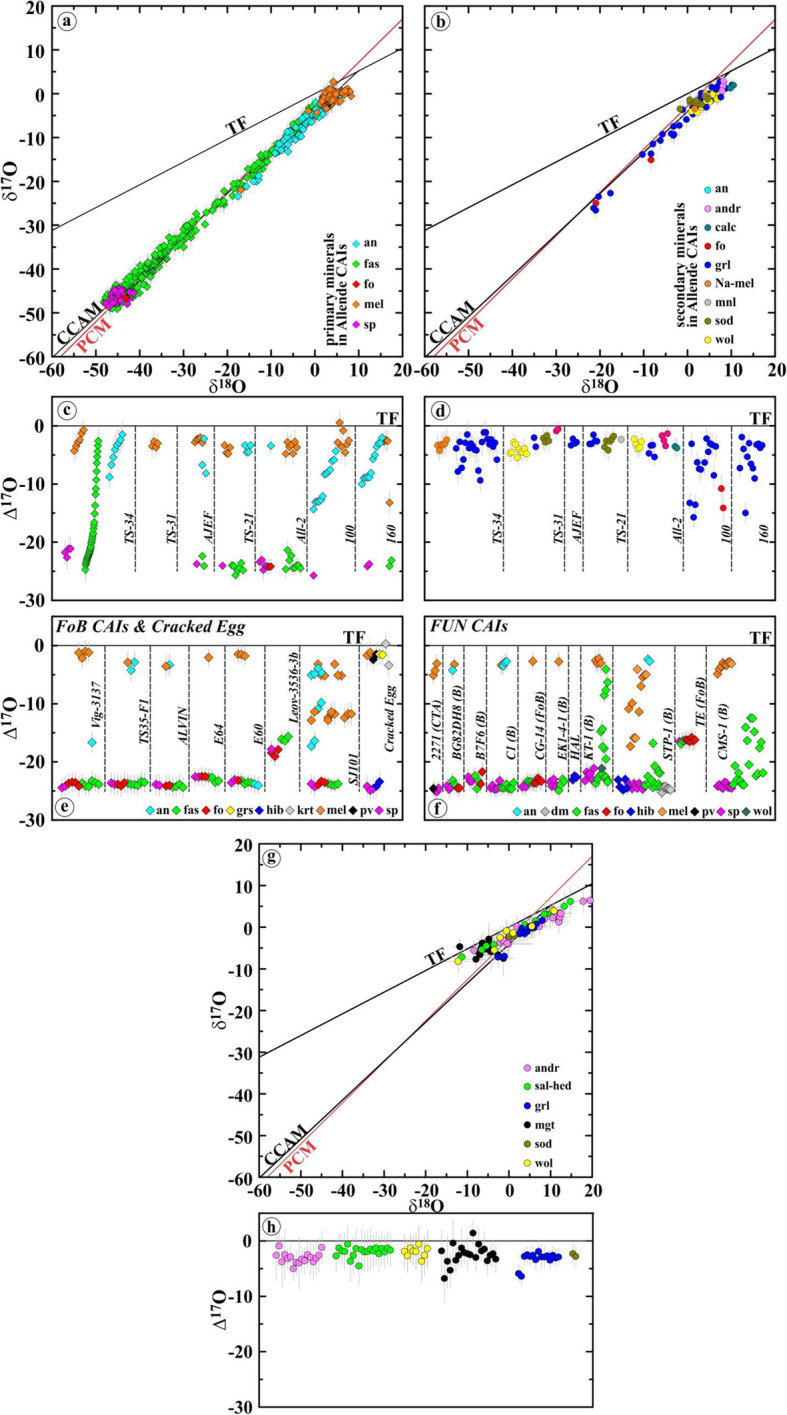
**a**, **b** Three-isotope oxygen diagrams and **c**, **d** ∆^17^O values of primary and secondary minerals in the Allende coarse-grained igneous CAIs *TS-34* (B1), *TS-31* (B2), *AJEF* (B1), *TS-21* (B2), *All-2* (FoB), *100* (C), and *160* (C)*.* Data for primary minerals in *TS-34* are from Kawasaki et al. (2018). Spinel and most analyses of Al,Ti-diopside have ^16^O-rich compositions; anorthite and melilite are ^16^O-depleted to various degrees. Al,Ti-diopside grains in *TS-34* show a large range of ∆^17^O that correlates with their igneous zoning: very Ti-rich pyroxene cores are ^16^O-depleted relative to Ti-poor pyroxene edges. Secondary minerals have generally ^16^O-poor compositions. The observed range of ∆^17^O of secondary minerals in individual CAIs is similar to those of primary melilite and anorthite. **e**, **f** ∆^17^O values of primary minerals in coarse-grained igneous CAIs of different types (CTA, B, FoB, and *Cracked Egg* that is rich in krotite (krt, CaAl_2_O_4_) and grossite (grs, CaAl_4_O_7_)) from CV chondrites. FUN (fractionation and unidentified nuclear effects) CAIs show large mass-dependent fractionation effects in oxygen isotopes indicating crystallization during melt evaporation in vacuum (Mendybaev et al. 2017). Melilite and anorthite in CV CAIs of all types are ^16^O-depleted relative to hibonite, spinel, forsterite, and fassaite. The observed O-isotope heterogeneity may reflect mineralogically controlled exchange with an aqueous fluid (∆^17^O = −3±2‰) on the CV parent asteroid. Gas-melt isotopic exchange in gaseous reservoirs of variable oxygen isotopic composition cannot be excluded. Data from Bullock et al. (2012), Connolly et al. (2019c), Krot et al. (2014), and Williams et al. (2017). **g** Three-isotope oxygen diagram and **h** ∆^17^O of secondary minerals in CAIs, chondrules, and matrices of the oxidized Allende-like CV chondrites. Most secondary minerals were measured without matrix-matched standards; magnetite is the only exception. Data from Choi et al. (1997), Krot et al. (2000, 2010), and Cosarinsky et al. (2008)

Oxygen isotopic compositions of the primary and secondary minerals in the Allende coarse-grained igneous CAIs measured here and plotted in Fig. 13a-d are listed in Supplementary Material (Tables EA2 and EA3, respectively).

### Aluminum-magnesium isotope systematics

Aluminum and magnesium isotopic compositions of grossular were measured with the UH Cameca ims-1280 using the method of Jacobsen et al. (2011). Briefly, a primary ^16^O^−^ ion beam with a current of ~150 pA focused to ~5×7 μm^2^ was used. ^24^Mg^+^, ^25^Mg^+^, and ^26^Mg^+^ were measured sequentially using a monocollector EM. ^27^Al^+^ was measured using a multicollection FC simultaneously with ^25^Mg^+^. The mass-resolving power was set to ~3800, sufficient to separate interfering hydrides and doubly charged ^48^Ca^2+^. The ^27^Al^+^/^24^Mg^+^ ratios were corrected using a sensitivity factor determined on a terrestrial grossular standard.

Magnesium isotopic compositions of melilite were measured simultaneously using 4 FCs. A ~10 nA ^16^O^−^ ion beam with ~20 μm size was used. The mass-resolving power was set to ~3800 using an exit slit width of 250 μm (multicollection exit slit #2). The count rates of ^24^Mg^+^ ranged from ~1×10^7^ to 5×10^7^ cps. The instrumental mass-fractionation effects and relative sensitivities were corrected using synthetic melilite glass. Excesses of ^26^Mg (^26^Mg*) were determined using an exponential law with an exponent of 0.514. We note differences in ^26^Mg* calculated using exponents of 0.514 and 0.5128 (Davis et al. 2015) are negligibly small (<0.03‰).

## Results and discussion

### Petrography and primary mineralogy of the Allende coarse-grained igneous CAIs

Based on the mineralogy, petrography, and bulk chemical compositions, several types of coarse-grained igneous CAIs are recognized in CV chondrites: compact type A (CTA), Type B (B1, B2), forsterite-bearing type B (FoB), and type C (e.g., Grossman 1980; Wark 1987; Wark et al. 1987; Simon et al. 1999; Simon and Grossman 2006; Bullock et al. 2012). Bulk chemical compositions of these CAIs projected from spinel onto the gehlenite (Ca_2_Al_2_SiO_7_)−forsterite (Mg_2_SiO_4_)−anorthite (CaAl_2_Si_2_O_8_) plane of the CaO−MgO−Al_2_O_3_−SiO_2_ (CMAS) system plot in the liquidus fields of melilite (CTA, B1, and B2; B2s are generally more SiO_2_-rich than B1s), forsterite (FoB), and anorthite (C).

In this section, we characterize the petrography and primary mineralogy of representative members of different CAI types studied (Fig. 1). Other Allende CAIs studied, their classification, and variations in chemical compositions of their melilite, one of the major silicate minerals that experienced metasomatic alteration, are listed in Table 1.

#### Type A CAIs

Type A Ca,Al-rich inclusions are a major type of coarse-grained refractory inclusions in CV3 chondrites. Type A CAIs, originally defined by Grossman (1975) as extremely melilite-rich inclusions with minor spinel and Al,Ti-diopside, were subsequently subdivided into irregularly shaped, fluffy ones (FTA), and round, compact (CTA) ones (Grossman 1980). Fluffy Type A CAIs are thought to have formed by gas-solid condensation (MacPherson and Grossman 1984), whereas CTA CAIs crystallized from melts (Simon et al. 1999). However, spinel and hibonite grains in FTA CAIs often have rounded or corroded outlines, and melilite grains often show inverse compositional zoning, probably reflecting incomplete melting and evaporation (Allen et al. 1978; Simon et al. 2001). As a result, there is a textural and mineralogical continuum between CTA and FTA CAIs. One of such intermediate FTA/CTA CAIs studied, *ALH-2*, is described below.

Fluffy/compact type A CAI *ALH-2* (Fig. 1a, b) consists mostly of gehlenitic melilite (Åk_1-24_), hibonite, spinel, perovskite, and traces of Al,Ti-diopside (MacPherson and Grossman 1984; Simon et al. 2001). Melilite ranges from small, prismatic crystals 40−70 μm across to anhedral grains ~ 200 μm across. The melilite grains show inverse chemical zoning with Åk-poor rims and relatively Åk-rich cores. The CAI is surrounded by a multilayered Wark-Lovering (WL) rim (from inside outward) of hibonite + spinel + perovskite, nearly pure gehlenite, and Al,Ti-diopside, a coarse-grained forsteritic olivine accretionary rim (Krot et al. 2001a), a fine-grained matrix-like rim, and a layer of Ca,Fe-rich silicates (salite-hedenbergite pyroxenes and andradite).

Compact type A CAIs, including those studied here (*818-G*, *CG-13/15*, *TS-68*, and *TS-2*) consist of melilite, spinel, Al,Ti-diopside (16−20 wt% TiO_2_, 18−21 wt% Al_2_O_3_), perovskite, ±hibonite, and ±platinum group element-bearing (PGE) metal nuggets. Melilite in most CTA CAIs is typically highly gehlenitic (Åk_<33_); åkermanite content increases towards the CAI cores. Individual melilite grains typically show normal igneous zoning. Nearly pure gehlenite (Åk_3_) occurs in and near WL rims. The anomalous CTA CAI *TS-2* (Fig. 1c−f), characterized in detail by Simon et al. (1998), shows a much larger compositional range of melilite (Åk_9−65_). Many melilite crystals consist of patches of åkermanitic melilite (Åk_32-62_, median Åk_52_) set in or partially enclosed by, and optically continuous with, relatively gehlenitic melilite (Åk_25-53_, median Åk_38_). The gehlenitic melilites have dendritic shapes and enclose numerous, fine inclusions of spinel and minor perovskite and Al,Ti-diopside. Coarse-grained spinel, ~50−150 μm across, occurs in clumps and chains enclosed in relatively åkermanitic melilite. The sample also contains a spinel-free palisade body composed almost entirely of Åk-rich (45−65 mol%) Na-bearing (up to 0.15 wt% Na_2_O) melilite. Melilite near WL rim is highly gehlenitic (Åk_9_).

#### Type B CAIs

Type B CAIs consist of melilite, Al,Ti-diopside, spinel, anorthite, and minor perovskite, hibonite, and ±PGE metal nuggets. A small subset of Type B inclusions contain igneous forsterite; these are classified as forsterite-bearing Type B CAIs (FoB, Bullock et al. 2012). Type Bs are subdivided into two textural subtypes: the B1s, which have a melilite-rich outer mantle enclosing a pyroxene-rich core; and the B2s, which have a rather homogeneous distribution of minerals (Wark and Lovering 1982b; Simon and Grossman 2006). There is a textural continuum between B1s and B2s. Melilite compositions of Type B1 and B2 inclusions are generally similar (Åk_5−75_). The composition of melilite in FoBs spans even a larger range (Åk_6−90_). There is a positive correlation between Åk and Na_2_O contents in Type Bs. Melilite in the outermost parts of type B1 mantles is generally more gehlenitic than that in B2s, FoBs, and cores of type B1s. Both normally and inversely zoned regions are observed within individual melilite grains in type Bs (MacPherson et al. 1984; Simon and Grossman 2006). Fassaite grains are igneously zoned with decreasing TiO_2_, Sc_2_O_3_, and V_2_O_3_ contents from cores to edges of the grains. Anorthite and spinel are compositionally pure.

Type B1 CAI *TS-23* (Fig. 1g−j) consists of melilite, Al,Ti-diopside (3−8 wt% TiO_2_; 15−20 wt% Al_2_O_3_), spinel, and anorthite (Grossman 1975; Hutcheon et al. 1978; MacPherson et al. 1984; Simon and Grossman 2006). It has a gehlenitic melilite mantle ~0.5-1 mm thick that encloses sparse spinel and fassaite grains and surrounds a relatively fassaite-rich and melilite-poor core. Melilite crystals (Åk_27-72_) in the core show complex (normal and reverse) compositional zoning (MacPherson et al. 1984). The CAI is surrounded by a spinel−Al-diopside WL rim, a fine-grained matrix-like rim composed of ferroan olivine and nepheline, and a layer of Ca,Fe-rich silicates composed of salite-hedenbergite pyroxenes, andradite, and wollastonite.

The anomalous Type B2 CAI *TS-31* (Fig. 1k, l) consists of highly åkermanitic melilite (Åk_76-87_; 0.1-0.2 wt% Na_2_O), sector-zoned Al,Ti-diopsides (TiO_2_, 4-11 wt%; Al_2_O_3_, 12-22 wt%), and compositionally pure anorthite and spinel. The peripheral portion of the CAI is extensively altered and contains small anhedral Ti-rich fassaites (TiO_2_, 17-20 wt%; Al_2_O_3_, 18-20 wt%). Wark-Lovering rim layers cannot be clearly defined due to extensive alteration. The CAI is surrounded by a fine-grained matrix-like rim composed of lath-shaped ferroan olivine and nepheline grains, and by a discontinuous layer of salite-hedenbergite pyroxenes + andradite + wollastonite.

Forsterite-bearing Type B CAI *Al-2* (Fig. 1m, n) consists mainly of Al,Ti-diopside (TiO_2_, 0.6-5.5 wt%; Al_2_O_3_, 4.7-21.7 wt%), highly åkermanitic Na-bearing melilite (Åk_79-87_, 0.12-0.23 wt% Na_2_O), and compositionally pure anorthite; all phases poikilitically enclose Ca-rich (1.0-1.3 wt% CaO) forsterite and spinel grains. Distribution of forsterite is very heterogeneous. Anorthite is preferentially concentrated in the peripheral part of the inclusion. The CAI is surrounded by a spinel-rich layer containing rare grains of hibonite (TiO_2_, 0.97 wt%; MgO, 8.5 wt%), a fine-grained matrix-like rim composed of lath-shaped ferroan olivine and nepheline, and a layer of salite-hedenbergite pyroxenes + andradite + wollastonite.

#### Type C CAIs

Type C CAI *160* is a fragmented inclusion partly surrounded by a WL rim of spinel and Al,Ti-diopside (Fig. 1o, p; see also Figs. 10 and 11 in Krot et al., 2007b). It is composed of Al,Ti-diopside, melilite, and spinel, all embedded into a fine-grained anorthite groundmass. Anorthite is compositionally pure CaAl_2_Si_2_O_8_. Al,Ti-diopside (TiO_2_, 4.6-13.9 w%; Al_2_O_3_, 18-20 wt%) occurs as coarse grains with abundant 3-5 μm-sized inclusions of anorthite (lacy pyroxenes; Wark 1987) and rounded inclusions of spinel, and as rare massive grains poikilitically enclosing euhedral spinel (see Fig. 11 in Krot et al., 2007b). Melilite occurs as coarse subhedral grains with abundant 3-5 μm-sized inclusions of anorthite (named “lacy melilite” by Wark 1987) and as compact polycrystalline regions with interstitial Al,Ti-diopside and poikilitically enclosing spinel. Melilite in the CAI mantle is more gehlenitic than in its core (Åk_44-56_ vs. Åk_56-72_); in both occurrences, melilite contains detectable Na_2_O (0.10-0.36 wt%) (Fig. 3 in Krot et al., 2007b). Spinel, nearly pure MgAl_2_O_4_, is heterogeneously distributed within the CAI and occurs as individual grains enclosed in melilite, anorthite and Al,Ti-diopside, and as framboids.

### Alteration of primary and secondary minerals in the Allende CAIs

#### Alteration of gehlenitic melilite in compact type A CAIs and type B1 CAIs

Gehlenitic melilite is common in CTAs and mantles of Type B1s. Nearly pure gehlenite occurs near WL in most CAIs studied. Highly gehlenitic melilite (Åk_<10_) in the outermost regions of all CAIs studied is replaced by nearly monomineralic anorthite/dmisteinbergite (Fig. 3a−c; hereafter anorthite; Raman or EBSD studies are required to distinguish between anorthite and dmisteinbergite; Fintor et al. 2014; Park et al. 2013). The anorthite/grossular ratio decreases towards the CAI cores (Fig. 3a−c), which correlates with the increase of åkermanite content in melilite (Åk_5−20_). Minor clintonite, spinel, and corundum occur in grossular-rich veins (Fig. 3d−g). Secondary spinel and corundum grains are generally less than 3 μm in size, and often form subhedral-to-euhedral grains (Fig. 3d−f, i, j). Occasionally, large concentrations of μm-sized spinel grains occupy central zones of some grossular-rich veins (Fig. 3e,f). Subhedral-to-anhedral clintonite grains generally occur in interstitial regions between grossular grains (Fig. 3g). In the clintonite-bearing regions, the compositionally distinct grossular grains are observed—FeO-poor/free and FeO-bearing; the latter typically overgrow the former and may associate with ferroan monticellite (monticellite−kirschsteinite solid solution) (Fig. 3g−j).

#### Alteration of åkermanitic melilite

Åkermanitic melilite is common in B2s, FoBs, Cs, cores of B1s, and CTA CAI *TS-2*. Several assemblages of secondary minerals replacing åkermanitic melilite are observed in the CAIs studied: (*i*) grossular + monticellite + wollastonite (Fig. 4a), (*ii*) grossular + monticellite (Fig. 4b−d), and (*iii*) grossular + Al-diopside (Fig. 4de, f). Minor wadalite (Fig. 4a−f), forsterite (Fig. 4b, g), clintonite (Fig. 4g−i), and spinel (Fig. 4i) can be found in all of them. In the clintonite-bearing regions, two compositionally distinct grossulars are observed with sharp boundaries between them—FeO-poor/free and FeO-bearing (Fig. 4i). In contrast to secondary minerals replacing gehlenitic melilite, secondary anorthite replacing åkermanitic melilite is virtually absent.

Changes in secondary mineral assemblages replacing melilite of different chemical compositions are illustrated in Fig. 5 showing a vein crosscutting a gehlenitic melilite mantle around Type B1 CAI *TS-23*. Near the WL rim, the vein consists predominantly of anorthite. Towards the CAI core, the abundance of anorthite decreases, whereas the abundance of grossular increases. In the anorthite-free regions of the grossular vein, minor Al-diopside, monticellite, and forsterite are observed. The abundance of Al-diopside is highest in a region where the vein enters the CAI core containing åkermanitic melilite (Fig. 5e). The åkermanitic melilite in the core is replaced by a coarse-grained mineral assemblage of grossular, monticellite, wollastonite, and wadalite (Fig. 5d).

#### Alteration of primary and secondary anorthite

Both primary and secondary anorthites in the peripheral portions of CAIs are replaced by nepheline, sodalite, and minor ferromagnesian olivine (Figs. 1 and 6). There are no clear textural relationships between nepheline and sodalite. Sodalite tends to be located closer to the CAI interiors than nepheline (Fig. 1d, l); however, this is not always the case (Fig. 1b, n). Ferromagnesian olivine ranges in composition from Fa~_10_ to Fa~_40_; fayalite content typically increases towards CAI peripheries.

In contrast to åkermanitic melilite, coarse-grained primary anorthite in type B and FoB CAI cores is generally only weakly altered. In contact with altered regions of åkermanitic melilite, the edges of anorthite grains appear to be corroded by Na-rich melilite and nearly pure grossular (Fig. 7a, b). In some cases, Na-rich melilite and grossular occur along cleavage planes of the anorthite grains (Fig. 7c). In others, subhedral grains of Na-melilite grow in open spaces within anorthite crystals (Fig. 7d, e). These subhedral grains of Na-melilite are often chemically zoned: sodium content increases towards grain edges. Occasionally, anorthite grains are crosscut by veins composed of Na-melilite, grossular, ± kushiroite, and ± wadalite (Fig. 7e, f).

#### Alteration of lacy melilite in Type C CAIs

In type C CAIs *160* and *100*, melilite and fassaite grains have “lacy” textures characterized by the presence of abundant rounded inclusions of anorthite ~ 5−10 μm in size. The lacy melilite and fasssaite are embedded in fine-grained (~ 5 μm in size) anorthite groundmass (Figs. 1 and 8o). Anorthite inclusions in lacy fassaite and melilite, and anorthite groundmass in the CAI cores show little evidence for alteration. At the same time, lacy melilite grains are pseudomorphed to varying degrees by grossular + forsterite + monticellite (Fig. 8a−d) and grossular + Al-diopside assemblages (Fig. d−f). Although the outlines of the original lacy melilite grains are generally well-preserved, the replaced portions are highly porous. The secondary grossular, forsterite, and Al-diopside growing into the pore spaces often show euhedral outlines (Fig. 8d, f). X-ray elemental mapping revealed enrichment in sodium along the peripheries of the pseudomorphed lacy melilites (see Fig. 2EA in Krot et al., 2007b). This enrichment is due to the presence of micron-sized anhedral grains of Na-rich melilite (Fig. 8b, d).

#### Secondary minerals replacing, overgrowing, and crosscutting earlier formed secondary phases

Some secondary minerals and/or mineral assemblages can be replaced, overgrown, and crosscut by newly formed ones, as illustrated in Figs. 9, 10, and 11. For example, (*i*) grossular + monticellite + wollastonite mineral assemblages replacing highly åkermanitic melilite in the FoB CAIs *All-2* and *All-5-2* are in turn replaced to a various degree by Al-diopside (Fig. 9a−d). (*ii*) In the Type B2 CAI *TS-31*, grossular + monticellite + wollastonite mineral assemblages replacing åkermanitic melilite are crosscut by wollastonite veins (Fig. 9e, f). (*iii*) In the Type B2 CAI *CG-10*, a wollastonite vein crosscuts an Al-diopside-rich region replacing an earlier formed grossular + monticellite_−_wollastonite assemblage (Fig. 10a); grossular in the Al-diopside-rich region shows oscillatory zoning (Fig. 10b). The coexisting oscillatory zoned grossular and Al-diopside are also found in the CAIs *AJEF* (B1, Fig. 10c), *CG-11* (B2, Fig. 10d, f), *TS-2* (CTA, Fig. 10e), and *CG-6* (B1, Fig. 10h). (*iv*) Occasionally, the earlier formed FeO-poor secondary minerals (Al-diopside, grossular, and monticellite) are either overgrown or crosscut by veins composed of ferroan grossular, ferroan monticellite, ferroan Al-diopside, hedenbergite, wollastonite, andradite, pentlandite, and heazlewoodite (Figs. 10 and 11g−i).

#### Secondary minerals in voids inside CAIs

Some coarse-grained igneous CAIs from Allende contain voids filled by anhedral-to-subhedral secondary grossular, monticellite, ferroan monticellite, wollastonite, tilleyite, calcite, andradite, hedenbergite, and Al-diopside (Fig. 12).

#### Secondary minerals around CAIs

All Allende CAIs studied are surrounded by the multilayered WL rims, fine-grained matrix-like rims, and a layer of Ca,Fe-rich silicates (Fig. 1). The peripheral regions of the CAIs contain abundant nepheline and sodalite (green and yellow grains, respectively, in Cl:Na:Al combined elemental maps) replacing primary and secondary anorthite. Sodalite tends to occur closer to the CAI interiors than nepheline. Sodium-free Cl-rich minerals, wadalite and/or adrianite (red grains in Cl:Na:Al maps; EBSD studies are required to distinguish between these minerals) occur almost exclusively in CAIs containing åkermanite-rich melilite—B2s, FoBs, cores of B1s, and the anomalous CTA CAI *TS-2*.

The fine-grained matrix-like rims consist of lath-shaped ferroan olivine, Fe,Ni-sulfides, and abundant nepheline grains typically enclosing lath-shaped ferroan olivine (Fig. 2). Occasionally, fine-grained rims contain salite-hedenbergite pyroxene nodules (Fig. 2b).

The calcium- and iron-rich silicate layers separate the fine-grained rims from the Allende matrix and consist of salite-hedenbergite pyroxenes, wollastonite, and andradite (Fig. 2c).

### Oxygen isotopic compositions

In Fig. 13a−d, we plotted oxygen isotopic compositions of primary and secondary minerals in the coarse-grained Allende CAIs measured with the matrix-matched mineral standards. In Fig. 13g, h, we plotted the previously reported data for secondary andradite, grossular, magnetite, salite-hedenbergite pyroxenes, sodalite, and wollastonite measured without matrix-matched standards (magnetite is the only exception) in the Allende matrix, chondrules, and dark inclusions. Like in the previously reported dataset, most secondary minerals in the Allende CAIs plot along mass-dependent fractionation line with ∆^17^O of ~ −3±2‰, but show a significantly smaller range of δ^18^O (~ 0 to + 10‰ vs. ~ −15 to ~ + 20‰). Larger ranges of ∆^17^O were found in grossular and forsterite in the Type B1 CAI *TS-34* (~ −10 to ~ −1‰) and Type C CAIs *100* and *160* (~ −15 to ~ −1‰); these minerals plot along the CCAM line. The similar ranges of ∆^17^O are observed for primary anorthite in these CAIs; melilite is generally more ^16^O-depleted (∆^17^O ~ −5 to ~ −1‰). Spinel and fassaite are ^16^O-rich (∆^17^O ~ −25 to ~ −20‰); the only exception is very Ti-rich pyroxenes in *TS-34*, which are ^16^O-depleted to various degrees.

### Aluminum-magnesium isotope systematics

**Fig. 14:**
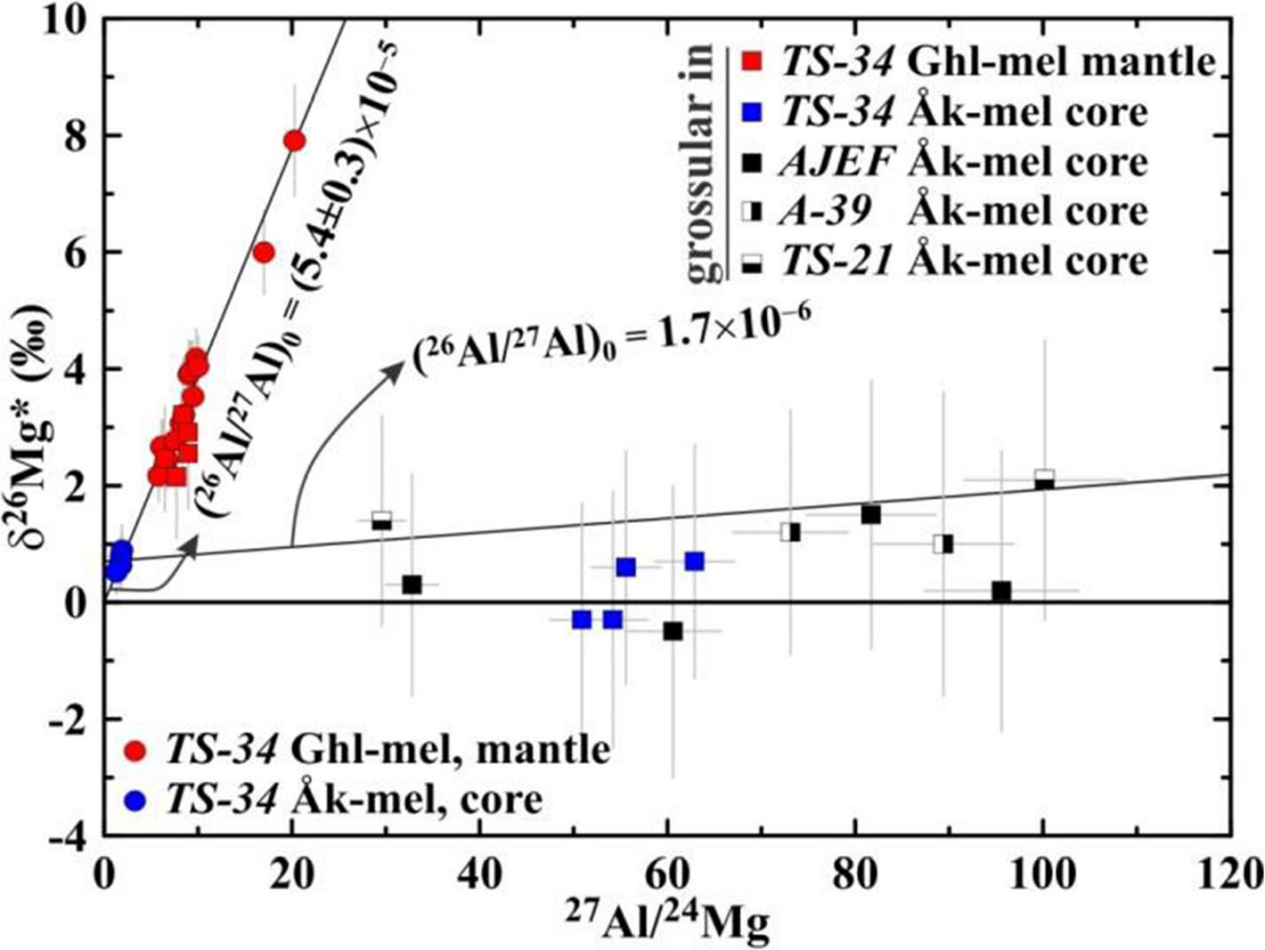
Aluminum-magnesium isotope diagram of primary melilite in *TS-34* (B1) and secondary grossular in *TS-34*, *AJEF* (B1), *A-39* (B2), and *TS-21* (B2). All melilite analyses show ^26^Mg* corresponding to the initial ^26^Al/^27^Al ratio, (^26^Al/^27^Al)_0_, of (5.4±0.3)×10^−5^, which is indistinguishable from the canonical (^26^Al/^27^Al)_0_ of 5.25×10^−5^. Grossular grains in nearly monomineralic veins crosscutting gehlenitic melilite mantle in *TS-34* show resolvable ^26^Mg* and plot along Al-Mg isochron defined by melilite; both minerals have similar ^27^Al/^24^Mg ratios. Coarse grossular grains replacing åkermanitic melilite and associating with monticellite, ±wollastonite, ±Al-diopside, and ±forsterite show no resolvable ^26^Mg*; Al-Mg isochron with (^26^Al/^27^Al)_0_ of 1.7×10^−6^ is shown for reference. We infer that replacement of melilite by grossular took place after nearly complete decay of ^26^Al; ^26^Mg* in the *TS-34* grossular replacing gehlenitic melilite was inherited by the melilite and provide no chronological information

In Fig. 14, we plotted excess of radiogenic ^26^Mg (^26^Mg*) vs. ^27^Al/^24^Mg ratio in gehlenitic melilite in the mantle and åkermanitic melilite in the core of the Type B CAI *TS-34*, and of secondary grossular replacing the melilite in this and several other Allende Type B CAIs. All melilite analyses in *TS-34* show resolvable ^26^Mg* and plot along correlation line corresponding to the initial ^26^Al/^27^Al ratio of (5.4±0.3)×10^−5^, which is indistinguishable from the canonical value of (5.25±0.02)×10^−5^ (Larsen et al. 2011). Grossular grains in veins crosscutting the gehlenitic melilite mantle in *TS-34* show resolvable ^26^Mg* and ^27^Al/^24^Mg ratio (in most cases, ~ 7−10) similar to those of melilite and plot along the Al-Mg isochron defined by the melilite. The coarse grossular grains replacing åkermanitic melilite and associating with monticellite, wollastonite, Al-diopside, and forsterite have significantly higher ^27^Al/^24^Mg ratios, ~30-100 (50−62 in *TS-34*), and show no resolvable ^26^Mg* within 2σ uncertainty of our measurements, ~±2‰.

### Secondary mineral assemblages in the Allende CAIs and inferred metasomatic reactions

#### Alteration of gehlenitic melilite

Highly gehlenitic melilite (Åk_<10_) in the outermost regions of the Allende CAIs is largely replaced and crosscut by veins of nearly monomineralic anorthite; grossular and corundum are minor. The abundance of anorthite in veins decreases and the abundance of grossular increases towards the CAI interiors containing more åkermanitic melilite (Åk_10−30_). Other secondary minerals observed in the grossular-rich veins include clintonite, spinel, and Al-diopside. The presence of clintonite provides clear evidence for the involvement of H_2_O in the alteration reactions, which can be expressed in mass-balanced form as the following:

4 $$ \underset{\mathrm{gehlenite}}{\mathrm{R}4:{\mathrm{Ca}}_2{\mathrm{Al}}_2{\mathrm{Si}\mathrm{O}}_{7\left(\mathrm{s}\right)}+{\mathrm{Si}\mathrm{O}}_2\to}\underset{\mathrm{anorthite}}{{\mathrm{Ca}\mathrm{Al}}_2{\mathrm{Si}}_2{\mathrm{O}}_{8\left(\mathrm{s}\right)}+\mathrm{CaO}} $$

5 $$ \underset{\mathrm{gehlenite}}{\mathrm{R}5:2{\mathrm{Ca}}_2{\mathrm{Al}}_2{\mathrm{Si}\mathrm{O}}_{7\left(\mathrm{s}\right)}+3{\mathrm{Si}\mathrm{O}}_2\to}\underset{\mathrm{grossular}}{{\mathrm{Ca}}_3{\mathrm{Al}}_2{\mathrm{Si}}_3{\mathrm{O}}_{12\left(\mathrm{s}\right)}+}\underset{\mathrm{anorthite}}{{\mathrm{Ca}\mathrm{Al}}_2{\mathrm{Si}}_2{\mathrm{O}}_{8\left(\mathrm{s}\right)}} $$

6 $$ \underset{\mathrm{gehlenite}}{\mathrm{R}6:3{\mathrm{Ca}}_2{\mathrm{Al}}_2{\mathrm{Si}\mathrm{O}}_{7\left(\mathrm{s}\right)}+3{\mathrm{Si}\mathrm{O}}_2\to}\underset{\mathrm{grossular}}{2{\mathrm{Ca}}_3{\mathrm{Al}}_2{\mathrm{Si}}_3{\mathrm{O}}_{12\left(\mathrm{s}\right)}+}\underset{\mathrm{corundum}}{{\mathrm{Al}}_2{\mathrm{O}}_{3\left(\mathrm{s}\right)}} $$

7 $$ \underset{\mathrm{gehlenite}-\overset{\circ }{a}\mathrm{kermanite}\ \mathrm{ss}}{\mathrm{R}7:2{\mathrm{Ca}}_2{\mathrm{Al}}_2{\mathrm{Si}\mathrm{O}}_7+{\mathrm{Ca}}_2{\mathrm{MgSi}}_2{\mathrm{O}}_{7\left(\mathrm{s}\right)}+2{\mathrm{Si}\mathrm{O}}_2\to}\underset{\mathrm{grossular}}{{\mathrm{Ca}}_3{\mathrm{Al}}_2{\mathrm{Si}}_3{\mathrm{O}}_{12\left(\mathrm{s}\right)}+}\underset{\mathrm{diopside}-\mathrm{Ca}-\mathrm{Tschermakite}\ \mathrm{ss}}{{\mathrm{Ca}\mathrm{MgSi}}_2{\mathrm{O}}_6+{\mathrm{Ca}\mathrm{Al}}_2{\mathrm{Si}\mathrm{O}}_{6\left(\mathrm{s}\right)}+\mathrm{CaO}} $$

8 $$ \underset{\mathrm{gehlenite}-\overset{\circ }{a}\mathrm{kermanite}\ \mathrm{ss}}{\mathrm{R}8:3{\mathrm{Ca}}_2{\mathrm{Al}}_2{\mathrm{Si}\mathrm{O}}_7+2{\mathrm{Ca}}_2{\mathrm{MgSi}}_2{\mathrm{O}}_{7\left(\mathrm{s}\right)}+{\mathrm{H}}_2\mathrm{O}\to}\underset{\mathrm{grossular}}{{\mathrm{Ca}}_3{\mathrm{Al}}_2{\mathrm{Si}}_3{\mathrm{O}}_{12\left(\mathrm{s}\right)}+}\underset{\mathrm{clintonite}}{{\mathrm{Ca}\mathrm{Mg}}_2{\mathrm{Al}}_4{\mathrm{Si}\mathrm{O}}_{10}{\left(\mathrm{OH}\right)}_{2\left(\mathrm{s}\right)}+6\mathrm{CaO}+3{\mathrm{Si}\mathrm{O}}_2} $$

9 $$ \underset{\mathrm{gehlenite}-\overset{\circ }{a}\mathrm{kermanite}\ \mathrm{ss}}{\mathrm{R}9:3{\mathrm{Ca}}_2{\mathrm{Al}}_2{\mathrm{Si}\mathrm{O}}_7+{\mathrm{Ca}}_2{\mathrm{MgSi}}_2{\mathrm{O}}_{7\left(\mathrm{s}\right)}+{\mathrm{Si}\mathrm{O}}_2\to}\underset{\mathrm{grossular}}{2{\mathrm{Ca}}_3{\mathrm{Al}}_2{\mathrm{Si}}_3{\mathrm{O}}_{12\left(\mathrm{s}\right)}+}\underset{\mathrm{spinel}}{{\mathrm{MgAl}}_2{\mathrm{O}}_{4\left(\mathrm{s}\right)}+2\mathrm{CaO}} $$

10 $$ \underset{\mathrm{gehlenite}-\overset{\circ }{a}\mathrm{kermanite}\ \mathrm{ss}}{\mathrm{R}10:2{\mathrm{Ca}}_2{\mathrm{Al}}_2{\mathrm{Si}\mathrm{O}}_7+{\mathrm{Ca}}_2{\mathrm{MgSi}}_2{\mathrm{O}}_{7\left(\mathrm{s}\right)}+2{\mathrm{Si}\mathrm{O}}_2+{\mathrm{Al}}_2{\mathrm{O}}_3\to}\underset{\mathrm{grossular}}{2{\mathrm{Ca}}_3{\mathrm{Al}}_2{\mathrm{Si}}_3{\mathrm{O}}_{12\left(\mathrm{s}\right)}+}\underset{\mathrm{spinel}}{{\mathrm{MgAl}}_2{\mathrm{O}}_{4\left(\mathrm{s}\right)}} $$

The released calcium (reactions (R) 4, 7, 8, and 9) could have precipitated as a layer of Ca,Fe-rich silicates (salite-hedenbergite pyroxenes, andradite and wollastonite) outside and inside fine-grained rims surrounding altered CAIs (see below).

#### Alteration of åkermanitic melilite

Åkermanitic melilite in Type B2s, FoBs, cores of B1s, and CTA CAI *TS-2* is replaced by three major secondary minerals assemblages: (*i*) grossular + monticellite + wollastonite (R11), (*ii*) grossular + monticellite (R12), and (*iii*) grossular + Al-diopside (R7); minor secondary minerals include clintonite, forsterite, and spinel. Again, the presence of clintonite and margarite (this study; Keller and Buseck 1991) in the secondary mineral assemblages requires the presence of H_2_O during alteration reactions:

11 $$ \underset{\mathrm{gehlenite}-\overset{\circ }{a}\mathrm{kermanite}\ \mathrm{ss}}{\mathrm{R}11:{\mathrm{Ca}}_2{\mathrm{Al}}_2{\mathrm{Si}\mathrm{O}}_{7\left(\mathrm{s}\right)}+3{\mathrm{Ca}}_2{\mathrm{MgSi}}_2{\mathrm{O}}_7+{\mathrm{Si}\mathrm{O}}_2\to}\underset{\mathrm{grossular}}{{\mathrm{Ca}}_3{\mathrm{Al}}_2{\mathrm{Si}}_3{\mathrm{O}}_{12\left(\mathrm{s}\right)}+}\underset{\mathrm{monticellite}\ }{3{\mathrm{Ca}\mathrm{MgSiO}}_{4\left(\mathrm{s}\right)}+}\underset{\mathrm{wollastonite}}{2{\mathrm{Ca}\mathrm{SiO}}_{3\left(\mathrm{s}\right)}} $$

12 $$ \underset{\mathrm{gehlenite}-\overset{\circ }{a}\mathrm{kermanite}\ \mathrm{ss}}{\mathrm{R}12:{\mathrm{Ca}}_2{\mathrm{Al}}_2{\mathrm{Si}\mathrm{O}}_{7\left(\mathrm{s}\right)}+2{\mathrm{Ca}}_2{\mathrm{MgSi}}_2{\mathrm{O}}_7\to}\underset{\mathrm{grossular}}{{\mathrm{Ca}}_3{\mathrm{Al}}_2{\mathrm{Si}}_3{\mathrm{O}}_{12\left(\mathrm{s}\right)}+}\underset{\mathrm{monticellite}}{2{\mathrm{Ca}\mathrm{MgSiO}}_{4\left(\mathrm{s}\right)}+\mathrm{CaO}} $$

13 $$ \underset{\mathrm{gehlenite}-\overset{\circ }{a}\mathrm{kermanite}\ \mathrm{ss}}{\mathrm{R}13:{\mathrm{Ca}}_2{\mathrm{Al}}_2{\mathrm{Si}\mathrm{O}}_{7\left(\mathrm{s}\right)}+{\mathrm{Ca}}_2{\mathrm{MgSi}}_2{\mathrm{O}}_7+{\mathrm{Si}\mathrm{O}}_2\to}\underset{\mathrm{grossular}}{{\mathrm{Ca}}_3{\mathrm{Al}}_2{\mathrm{Si}}_3{\mathrm{O}}_{12\left(\mathrm{s}\right)}+}\underset{\mathrm{monticellite}}{{\mathrm{Ca}\mathrm{MgSiO}}_{4\left(\mathrm{s}\right)}} $$

In some cases, grossular + monticellite + wollastonite assemblages are subsequently replaced by Al-diopside (Fig. 9a-d):

14 $$ \underset{\mathrm{grossular}}{\mathrm{R}14:{\mathrm{Ca}}_3{\mathrm{Al}}_2{\mathrm{Si}}_3{\mathrm{O}}_{12\left(\mathrm{s}\right)}+}\underset{\mathrm{monticellite}}{{\mathrm{Ca}\mathrm{MgSi}\mathrm{O}}_{4\left(\mathrm{s}\right)}+}\underset{\mathrm{wollastonite}}{{\mathrm{Ca}\mathrm{SiO}}_{3\left(\mathrm{s}\right)}\to}\underset{\mathrm{diopside}-\mathrm{Ca}-\mathrm{Tschermakite}\ \mathrm{ss}}{{\mathrm{Ca}\mathrm{MgSi}}_2{\mathrm{O}}_{6\left(\mathrm{s}\right)}+{\mathrm{Ca}\mathrm{Al}}_2{\mathrm{Si}\mathrm{O}}_{6\left(\mathrm{s}\right)}+2{\mathrm{Si}\mathrm{O}}_2+3\mathrm{CaO}} $$

The released calcium and silica may have been used to form a new generation of wollastonite that either crosscuts the earlier formed secondary mineral assemblages or fills the voids in the CAI interiors (Figs. 9 and 10a):

15 $$ \mathrm{R}15:\mathrm{CaO}+{\mathrm{SiO}}_2\to \underset{\mathrm{wollastonite}}{{\mathrm{CaSiO}}_{3\left(\mathrm{s}\right)}} $$

These elements may have been also used to form a layer of Ca,Fe-rich silicates (salite-hedenbergite pyroxenes, andradite, and wollastonite) outside fine-grained rims around CAIs (see below).

#### Alteration of lacy melilite

In contrast to the preferential replacement of coarse åkermanitic melilite grains in Type B and FoB CAIs, in Type Cs, lacy melilite grains composed of åkermanitic melilite and anorthite are pseudomorphically replaced by the grossular + monticellite + forsterite (R16) and by grossular + Al-diopside assemblages (R17). The pseudomorphs contain neither clintonite nor margarite, and are surrounded by the virtually unaltered fine-grained anorthite groundmass, suggesting the alteration may have occurred under nearly dry conditions:

16 $$ \underset{\mathrm{gehlenite}-\overset{\circ }{a}\mathrm{kermanite}\ \mathrm{ss}}{\mathrm{R}16:{\mathrm{Ca}}_2{\mathrm{Al}}_2{\mathrm{Si}\mathrm{O}}_{7\left(\mathrm{s}\right)}+3{\mathrm{Ca}}_2{\mathrm{Mg}\mathrm{Si}}_2{\mathrm{O}}_7+}\underset{\mathrm{anorthite}}{2{\mathrm{Ca}\mathrm{Al}}_2{\mathrm{Si}}_2{\mathrm{O}}_{8\left(\mathrm{s}\right)}\to}\underset{\mathrm{grossular}}{3{\mathrm{Ca}}_3{\mathrm{Al}}_2{\mathrm{Si}}_3{\mathrm{O}}_{12\left(\mathrm{s}\right)}+}\underset{\mathrm{monticellite}}{{\mathrm{Ca}\mathrm{MgSiO}}_{4\left(\mathrm{s}\right)}+}\underset{\mathrm{forsterite}}{{\mathrm{Mg}}_2{\mathrm{Si}\mathrm{O}}_{4\left(\mathrm{s}\right)}} $$

17 $$ \underset{\mathrm{gehlenite}-\overset{\circ }{a}\mathrm{kermanite}\ \mathrm{ss}}{\mathrm{R}17:{\mathrm{Ca}}_2{\mathrm{Al}}_2{\mathrm{Si}\mathrm{O}}_{7\left(\mathrm{s}\right)}+2{\mathrm{Ca}}_2{\mathrm{MgSi}}_2{\mathrm{O}}_7+}\underset{\mathrm{anorthite}}{{\mathrm{Ca}\mathrm{Al}}_2{\mathrm{Si}}_2{\mathrm{O}}_{8\left(\mathrm{s}\right)}+{\mathrm{Si}\mathrm{O}}_2\to}\underset{\mathrm{grossular}}{{\mathrm{Ca}}_3{\mathrm{Al}}_2{\mathrm{Si}}_3{\mathrm{O}}_{12\left(\mathrm{s}\right)}+}\underset{\mathrm{diopside}-\mathrm{Ca}-\mathrm{Tschermakite}\ \mathrm{ss}}{2{\mathrm{Ca}\mathrm{MgSi}}_2{\mathrm{O}}_6+{\mathrm{Ca}\mathrm{Al}}_2{\mathrm{Si}\mathrm{O}}_{6\left(\mathrm{s}\right)}+\mathrm{CaO}} $$

18 $$ \underset{\mathrm{gehlenite}-\overset{\circ }{a}\mathrm{kermanite}\ \mathrm{ss}}{\mathrm{R}18:{\mathrm{Ca}}_2{\mathrm{Al}}_2{\mathrm{Si}\mathrm{O}}_{7\left(\mathrm{s}\right)}+{\mathrm{Ca}}_2{\mathrm{MgSi}}_2{\mathrm{O}}_7+}\underset{\mathrm{anorthite}}{{\mathrm{Ca}\mathrm{Al}}_2{\mathrm{Si}}_2{\mathrm{O}}_{8\left(\mathrm{s}\right)}+{\mathrm{Si}\mathrm{O}}_2\to}\underset{\mathrm{grossular}}{{\mathrm{Ca}}_3{\mathrm{Al}}_2{\mathrm{Si}}_3{\mathrm{O}}_{12\left(\mathrm{s}\right)}+}\underset{\mathrm{diopside}-\mathrm{Ca}-\mathrm{Tschermakite}\ \mathrm{ss}}{2{\mathrm{Ca}\mathrm{MgSi}}_2{\mathrm{O}}_6+{\mathrm{Ca}\mathrm{Al}}_2{\mathrm{Si}\mathrm{O}}_{6\left(\mathrm{s}\right)}} $$

The pseudomorphically replaced lacy melilite grains are surrounded by a thin discontinuous rim of Na-rich melilite (Fig. 8b, d). Because the primary åkermanitic melilite contains 0.1-0.3 wt% Na_2_O (Table 1), it is possible that sodium in the Na-rich melilite may have had a local origin, i.e., it may be a byproduct of alteration of the primary Na-bearing åkermanitic melilite. The preferential occurrence of Na-melilite along edges of lacy melilite pseudomorphs may reflect normal igneous zoning of the lacy melilite grains: a positive correlation between Na_2_O and åkermanite content in melilite was described in many igneous CV CAIs (e.g., Beckett and Grossman 1988; Beckett et al. 2000; Simon and Grossman 2006). A similar origin could be inferred for Na-melilite in all Allende containing Na-bearing igneous åkermanitic melilite (Fig. 7).

#### Alteration of primary and secondary anorthite

The occurrences of primary igneous anorthite depend on the CAI types: In type B1 CAIs, it occurs in the CAI cores; in Type B2 CAIs, it occurs throughout the inclusions (Fig. 1g, k, m). Secondary anorthite occurs almost exclusively in the peripheral regions of the Allende CAIs where it replaces gehlenitic melilite (Fig. 1). Primary and secondary anorthites in the peripheral portions of the Allende CAIs are largely replaced by nepheline, sodalite, and ferromagesian olivine:

19 $$ \underset{\mathrm{anorthite}}{\mathrm{R}19:{\mathrm{CaAl}}_2{\mathrm{Si}}_2{\mathrm{O}}_{8\left(\mathrm{s}\right)}+{\mathrm{Na}}_2\mathrm{O}\to}\underset{\mathrm{nepheline}}{2{\mathrm{Na}\mathrm{AlSiO}}_{4\left(\mathrm{s}\right)}+\mathrm{CaO}} $$

20 $$ \underset{\mathrm{anorthite}}{\mathrm{R}20:3{\mathrm{CaAl}}_2{\mathrm{Si}}_2{\mathrm{O}}_{8\left(\mathrm{s}\right)}+3{\mathrm{Na}}_2\mathrm{O}+2\mathrm{NaCl}\to}\underset{\mathrm{sodalite}}{{\mathrm{Na}}_8{\mathrm{Al}}_6{\mathrm{Si}}_6{\mathrm{O}}_{24}{\mathrm{Cl}}_{2\left(\mathrm{s}\right)}+3\mathrm{CaO}} $$

The magnesium content in primary and secondary anorthite is too low and iron is essentially absent, so the presence of secondary ferromagnesian olivine associated with nepheline and sodalite requires an external source of these elements, perhaps matrix. We suggest that Mg, Fe, and likely SiO_2_ were supplied to the host inclusions by an external aqueous solution:

21 $$ \mathrm{R}21:{{\mathrm{Mg}}^{2+}}_{\left(\mathrm{aq}\right)}+{{\mathrm{Fe}}^{2+}}_{\left(\mathrm{aq}\right)}+{\mathrm{SiO}}_{2\left(\mathrm{aq}\right)}+2{\mathrm{H}}_2{\mathrm{O}}_{\left(\mathrm{aq}\right)}\to \underset{\mathrm{fayalite}-\mathrm{forsterite}\ \mathrm{ss}}{{\left(\mathrm{Fe},\mathrm{Mg}\right)}_2{\mathrm{SiO}}_{4\left(\mathrm{s}\right)}+4{{\mathrm{H}}^{+}}_{\left(\mathrm{aq}\right)}} $$

### Zoned distribution of Na- and Cl-bearing minerals and Ca,Fe-rich silicates in and around Allende CAIs: Evidence for in situ open-system metasomatic alteration of the Allende CAIs

The observed depletion in calcium and enrichment in silicon in altered gehlenitic melilite regions, the presence of a Ca,Fe-rich silicate layer outside fine-grained rims around altered CAIs, and the zoned distribution of Na- and Cl-bearing minerals (nepheline, sodalite, and wadalite) in and around Allende CAIs (Fig. 1) provide clear evidence for an open-system behavior of several elements (Si, Na, Cl, Ca, and Al) during in situ metasomatic alteration of these CAIs: Si, Na, and Cl were added, whereas Ca and, probably, some Al were removed from the inclusions. The mobility of aluminum during the alteration is consistent with the presence of grossular, kushiroite, and Na-rich melilite in veins crosscutting primary anorthite grains inside the inclusions (e.g., Fig. 7f). The outward transport of dissolved Al from CAIs and the influx of Na, K, and Si with an aqueous fluid equilibrated with the Allende matrix could explain the abundant nepheline grains in fine-grained rims around Allende CAIs studied (Figs. 1 and 2):

22 $$ \mathrm{R}22:{{\mathrm{Na}}^{+}}_{\left(\mathrm{aq}\right)}+{{\mathrm{Al}}^{3+}}_{\left(\mathrm{aq}\right)}+{\mathrm{SiO}}_{2\left(\mathrm{aq}\right)}+2{\mathrm{H}}_2{\mathrm{O}}_{\left(\mathrm{aq}\right)}\to \underset{\mathrm{nepheline}}{{\mathrm{Na}\mathrm{AlSiO}}_{4\left(\mathrm{s}\right)}+4{{\mathrm{H}}^{+}}_{\left(\mathrm{aq}\right)}} $$

The fact that fine-grained rims lack sodalite may indicate an earlier formation of nepheline than sodalite. A similar conclusion was reached by Che and Brearley (2021) based on the zoned distribution of nepheline and sodalite within Allende CAIs.

Wark (1981) interpreted the presence of alkali-rich halos around altered Allende CAIs as an evidence for outward diffusion of volatiles and suggested that the alteration occurred in the solar nebula prior to incorporation of CAIs into the Allende meteorite. We conclude instead that this interpretation is incorrect and cannot be used as evidence for nebular alteration.

Calcium released during replacement of gehlenitic melilite by anorthite (R4) and subsequently by nepheline and sodalite (R19, R20) may have interacted with Fe-, Mg- and Si-enriched fluid that most likely was in equilibrium with the Allende fine-grained matrix (Krot et al. 2001b; Ganino and Libourel 2017). This process resulted in precipitation of Ca,Fe-rich silicates at the boundary between fine-grained rims and the Allende matrix (Figs. 1 and 2) and, occasionally, within fine-grained rims (Fig. 2b):

23 $$ \mathrm{R}23:{{\mathrm{Ca}}^{2+}}_{\left(\mathrm{aq}\right)}+{{\mathrm{Fe}}^{2+}}_{\left(\mathrm{aq}\right)}+2{\mathrm{SiO}}_{2\left(\mathrm{aq}\right)}+2{\mathrm{H}}_2{\mathrm{O}}_{\left(\mathrm{aq}\right)}\to \underset{\mathrm{hedenbergite}}{{\mathrm{Ca}\mathrm{FeSi}}_2{\mathrm{O}}_{6\left(\mathrm{s}\right)}+4{{\mathrm{H}}^{+}}_{\left(\mathrm{aq}\right)}} $$

24 $$ \mathrm{R}24:3{{\mathrm{Ca}}^{2+}}_{\left(\mathrm{aq}\right)}+2{{\mathrm{Fe}}^{3+}}_{\left(\mathrm{aq}\right)}+3{\mathrm{Si}\mathrm{O}}_{2\left(\mathrm{aq}\right)}+6{\mathrm{H}}_2{\mathrm{O}}_{\left(\mathrm{aq}\right)}\to \underset{\mathrm{andradite}}{{\mathrm{Ca}}_3{\mathrm{Fe}}_2{\mathrm{Si}}_3{\mathrm{O}}_{12\left(\mathrm{s}\right)}+12{{\mathrm{H}}^{+}}_{\left(\mathrm{aq}\right)}} $$

25 $$ \mathrm{R}25:{{\mathrm{Ca}}^{2+}}_{\left(\mathrm{aq}\right)}+{\mathrm{SiO}}_{2\left(\mathrm{aq}\right)}+{\mathrm{H}}_2{\mathrm{O}}_{\left(\mathrm{aq}\right)}\to \underset{\mathrm{wollastonite}}{{\mathrm{Ca}\mathrm{SiO}}_{3\left(\mathrm{s}\right)}+2{{\mathrm{H}}^{+}}_{\left(\mathrm{aq}\right)}} $$

26 $$ \mathrm{R}26:{{\mathrm{Ca}}^{2+}}_{\left(\mathrm{aq}\right)}+0.5{{\mathrm{Fe}}^{2+}}_{\left(\mathrm{aq}\right)}+0.5{{\mathrm{Mg}}^{2+}}_{\left(\mathrm{aq}\right)}+2{\mathrm{Si}\mathrm{O}}_{2\left(\mathrm{aq}\right)}+2{\mathrm{H}}_2{\mathrm{O}}_{\left(\mathrm{aq}\right)}\to \underset{\mathrm{salite}-\mathrm{hedenbergite}\ \mathrm{ss}}{\mathrm{Ca}\left(\mathrm{Fe},\mathrm{Mg}\right){\mathrm{Si}}_2{\mathrm{O}}_{6\left(\mathrm{s}\right)}+4{{\mathrm{H}}^{+}}_{\left(\mathrm{aq}\right)}} $$

The preferential precipitation of Ca,Fe-rich silicates outside fine-grained rims may be due to a relatively compact nature of these rims compared to the boundaries between rims and matrix, where a geochemical barrier between fluids of different compositions, Ca-rich and Fe,Mg,Si-rich, which were in equilibrium with the host CAIs and surrounding matrix, respectively, could have been generated.

### Evidence for multistage metasomatic alteration of the Allende CAIs and variations in chemical composition of the metasomatic fluids

Our mineralogical observations indicate that metasomatic alteration of the Allende coarse-grained CAIs was multistage and resulted in the formation of multiple generations of secondary minerals. These include (*i*) replacement of grossular + monticellite + wollastonite mineral assemblages by Al-diopside (Fig. 9a−d), (*ii*) wollastonite veins crosscutting grossular + monticellite + wollastonite mineral assemblages (Fig. 9e, f), (*iii*) crosscutting relationships between grossular-rich veins and veins containing abundant iron-rich minerals (andradite, hedenbergite, ferroan Al-diopside, ferroan monticellite, pentlandite, and heazlewoodite) (Fig. 11); (*iv*) overgrowth of monticellite by ferroan monticellite (Fig. 10i); (*v*) overgrowth of Al-diopside by ferroan grossular (Fig. 10g); (*vi*) overgrowth of grossular by ferroan grossular and andradite (Figs. 3 and 10g-j and h); (*vii*) oscillatory zoning of grossular and Al-diopside grains (Fig. 10b-h); (*viii*) replacement of secondary anorthite by nepheline, sodalite, and ferromagnesian olivine (Figs. 1 and 6c, d). In most cases, the later formed secondary minerals contain higher iron content than the earlier ones. These observations suggest that alteration occurred in the presence of aqueous solutions of variable chemical compositions.

### Physico-chemical analysis of alteration reactions inferred in Allende CAIs

#### Transport of elements in aqueous solution and steam during metasomatic alteration of Allende CAIs

Mineralogical and petrographic observations "Petrography and primary mineralogy of the Allende coarse-grained igneous CAIs", chemical compositions of secondary phases, and the inferred chemical reactions "Secondary mineral assemblages in the Allende CAIs and inferred metasomatic reactions", "Zoned distribution of Na- and Cl-bearing minerals and Ca,Fe-rich silicates in and around Allende CAIs: Evidence for in situ open-system metasomatic alteration of the Allende CAIs", "Evidence for multistage metasomatic alteration of the Allende CAIs and variations in chemical composition of the metasomatic fluids" suggest the involvement of water in metasomatic alteration of CV chondrites and their components (see also Brearley and Krot 2013; Ganino and Libourel 2017). The altered patches and veins in mantles and cores of the Allende coarse-grained CAIs are dominated by Ca, Al, Mg-rich phases which are enriched in SiO_2_ and depleted in CaO compared to the primary phases. Such mineralogy requires an efficient addition of Si to and removal of Ca from CAIs. Most secondary ferromagnesian silicates in CAI interiors—forsterite, monticellite, and Al-diopside—are essentially Fe-free compared to the ferroan olivine, kirschsteinite, and salite-hedenbergite in fine-grained rims and neighboring matrix. It appears that for some reason the Fe, abundant in an aqueous solution circulating through the Allende matrix (Krot et al. 1998a, 1998b; Ganino and Libourel 2017), has not been efficiently delivered to the interiors of the Allende coarse-grained igneous CAIs. The same may be true for Na and Cl which are predicted to have high concentrations in an aqueous solution equilibrated with matrix (Petaev and Moronenko 1997), but in wadalite replacing åkermanitic melilite in Type B CAIs, these elements are decoupled from each other.

While the source of water on chondritic parent bodies remains speculative, the accretion of H_2_O-rich ices alongside other nebular components is considered the most plausible mechanism (Krot et al. 2015). Upon subsequent heating, either internal or impact-induced, the ices sublimate and/or melt to generate H_2_O steam or liquid water or, most likely, both. The liquid water and steam would react with the surrounding minerals and amorphous phases and leach out some chemical elements to transform initially nearly pure water to an aqueous solution with concentrations of SiO_2_, Fe^2+^, Mn^2+^, Ca^2+^, Mg^2+^, Na^+^, Cl^−^, HCO_3_^−^, and other ions and neutral aqueous species gradually increasing with time. Such a solution is capable transporting of Ca, Mn, Fe, Si, Na, and Cl at low temperatures. The ability of steam to transport at least some of these elements depends upon P-T conditions. The numerous experimental and natural data suggest that in terrestrial hydrothermal environments a supercritical fluid (T > 647 K, P > 22 MPa) can efficiently transport these elements too, but the estimates of the Allende tensile strength imply that a gas pressure in excess of ~ 10 MPa should break the rock and release the gas (Grimm and McSween Jr. 1989). Thus, the presence of supercritical fluid on the CV parent body seems unlikely. Still, a low-density steam, either subcritical saturated (P_H2O_ = P_H2O, equil_, T < 647 K) or dry superheated (P_H2O_ < P_H2O, equil_, T > 647 K), can spread out of the aqueous solution source region and carry chemical species dissolved in it. Such a steam seems to be of low interest for geochemists, but it is a source of great concern in the steam power industry because it results in the deposition of solids on turbine blades and corrodes them.

A classic review of experimental data on concentrations of different solutes in subcritical and dry superheated steam (Harvey and Bellows 1997) shows that significant amounts of SiO_2_, NaCl, NaOH, Na_2_SO_4_, Na_3_PO_4_, and Fe oxides orders of magnitude higher than would be expected on their vapor pressures, can be dissolved in steam in the form of solvated complexes (e.g., SiO_2_•mH_2_O). The concentration of a solute (X_s_) in the steam depends upon temperature (T), pressure (P), and steam density (ρ) as described by empirical equations

27 $$ \ln\ \left({\mathrm{X}}_{\mathrm{s}}\right)=\mathrm{A}+\mathrm{B}/\mathrm{T}+\left({\mathrm{m}}_1+{\mathrm{m}}_3/\mathrm{T}\right)\times \ln \rho -\mathrm{lnP} $$

or

28 $$ \ln \left({\mathrm{X}}_{\mathrm{s}}\right)=\mathrm{A}+\mathrm{D}\times \mathrm{T}+\left({\mathrm{m}}_1+{\mathrm{m}}_2\times \rho \times {\mathrm{m}}_3\times \mathrm{T}\right)\times \ln \rho -\mathrm{lnP} $$

where A, B, D, m_1_, m_2_, and m_3_ are regression coefficients specific for each solute. Because of generally low distribution coefficients of these solutes between a “parental” aqueous solution and the steam equilibrated with it, subsequent condensation of such a steam would produce a “secondary” aqueous solution containing much lower concentrations of solutes than its “parental” solution. The “secondary” solution should be essentially free of solutes which cannot be efficiently transported by the steam. Because of the low solubility of iron oxides in water under oxidizing conditions, the equilibrium concentrations of iron oxides in the steam are negligible. Calcium concentrations in steam are also expected to be low because of very low distribution coefficients of CaCl_2_ and CaSO_4_ between an aqueous solution and steam derived from it (e.g., Svoboda 2006). Apparently, neither Fe nor Ca can be efficiently transported by the steam.

#### Physico-chemical analysis of alteration reactions

The inferences about the involvement of an aqueous solution or H_2_O steam or both in the alteration process can be made based on a physicochemical analysis of the observed and/or possible alteration reactions and the textural relationships and mineral crystallization sequences in altered refractory inclusions. The textures and mineral chemistry of altered areas leave no doubt that an aqueous alteration was a non-equilibrium, multistage process, with many observed reactions not going to completion (see Alteration of primary and secondary minerals in the Allende CAIs). Given the multitude of such non-equilibrium effects, considering them would make the problem intractable. Instead, we employ here an equilibrium approach in evaluating intensive parameters of the observed alteration reactions assuming that they have reached a steady state. Then, in order to evaluate the plausibility of these reactions, the calculated values of fugacities and activities of different gaseous and aqueous species for each reaction are compared with each other and with independent estimates of these parameters. The thermodynamic data for solid, gaseous, and aqueous species are taken from compilations of Robie and Hemingway (1995) and Oelkers et al. (1995), except for the gaseous H_4_SiO_4_ (Plyasunov 2011) and sodalite (Komada et al. 1995). The gaseous and solid solutions are treated as ideal. For the sake of temperature interpolation, the tabulated data were fitted with polynomials, with the deviation between fitted and tabulated data being typically < 0.1%. For the aqueous species, we have adopted the lowest pressure (0.004–16.55 MPa) tabulation set for the 25–350 °C temperature range, with the pressure at any given temperature being equal to the partial pressure of the saturated H_2_O vapor; i.e., the gaseous phase involved in alteration reactions is nearly pure H_2_O steam equilibrated with an aqueous solution at any given temperature.

**Fig. 15:**
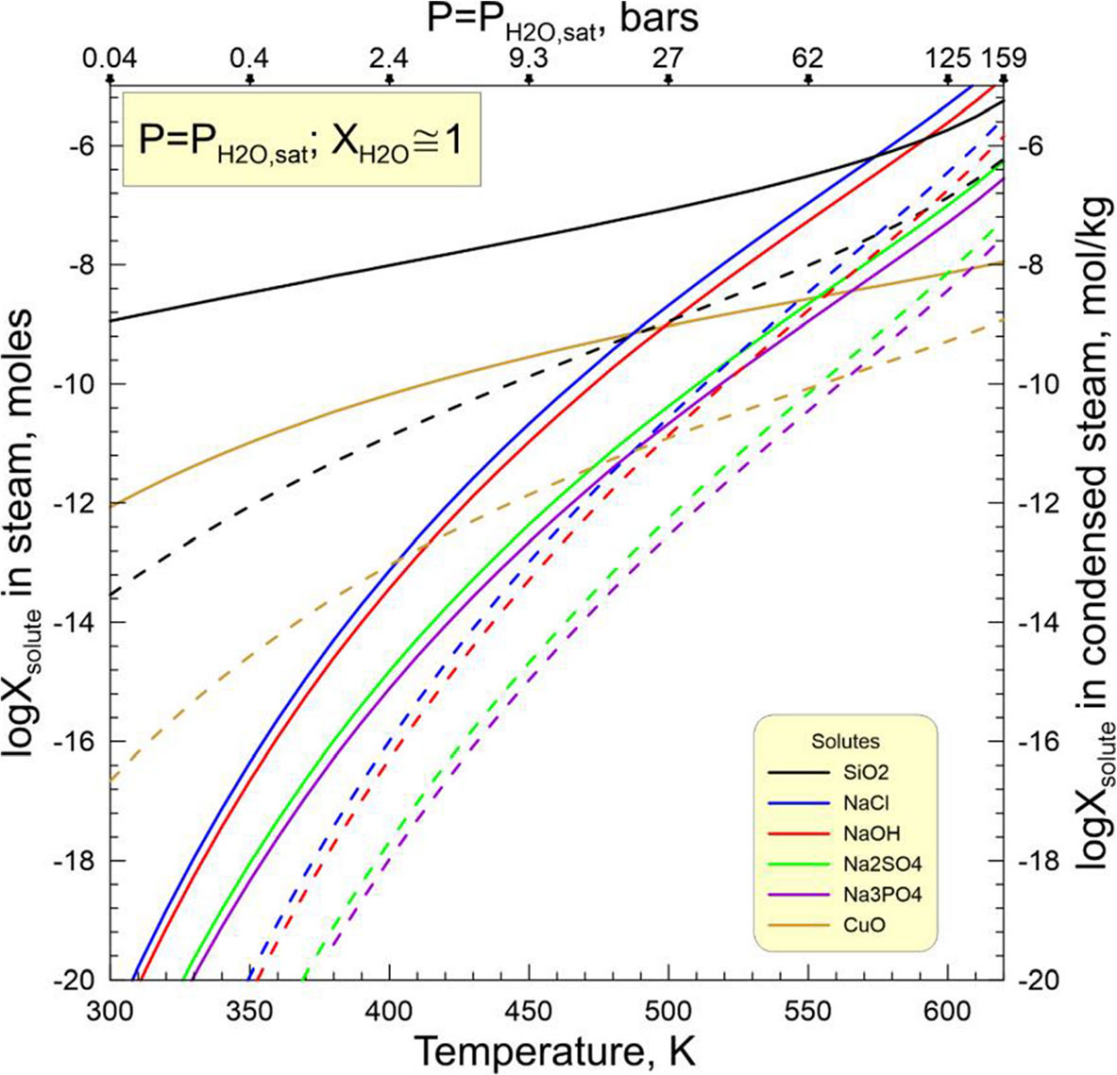
Maximium solubility of different solutes in the saturated H_2_O steam (solid lines) and the aqueous solution formed by its condensation (dashed lines)

For the same conditions, we calculated maximum concentrations of solutes in the steam and the aqueous solution formed by its condensation (Fig. 15), using equations and regression parameters of Harvey and Bellows (1997). The rather high concentrations of SiO_2_, Na, and Cl in both the steam and aqueous solution, especially at higher temperatures and pressures, imply that these elements potentially can be supplied to the CAIs from an aqueous solution equilibrated with matrix without the addition of Fe and Ca insoluble in steam. This may explain the Fe-rich and Fe-poor secondary mineralogy in the matrix and refractory inclusions, respectively. However, because both Na and Cl can be efficiently transported by the steam, they are not expected to decouple unless steam formed at an early stage of alteration contains only Cl-bearing species such as HCl and NH_4_Cl which might have been present in accreted ices (e.g., Zolotov and Mironenko 2007).

Because SiO_2_, CO_2_, Na, and Cl can be transported by steam, the reaction involving these elements do not require an aqueous solution to be in direct contact with the solid phases, while the reactions involving Ca, Fe, and Mg need an aqueous solution for addition and/or removal of these elements. The inferred alteration reactions are divided into several groups based on chemical elements added to and/or removed from the primary mineral assemblages of CAIs: (1) SiO_2_ only, (2) CO_2_ only, (3) SiO_2_, CO_2_, and Ca, (4) Na±Cl and Ca±SiO_2_, (5) Fe, Mg, Ca, SiO_2_.

The physico-chemical conditions of reactions describing the formation of Fe, Mg, Ca-bearing silicates during aqueous alteration have been previously evaluated by us (Krot et al. 1998b, 2001b) and are not considered here. They should be fully consistent with the current work because we use essentially the same approach and thermodynamic databases.

**Fig. 16:**
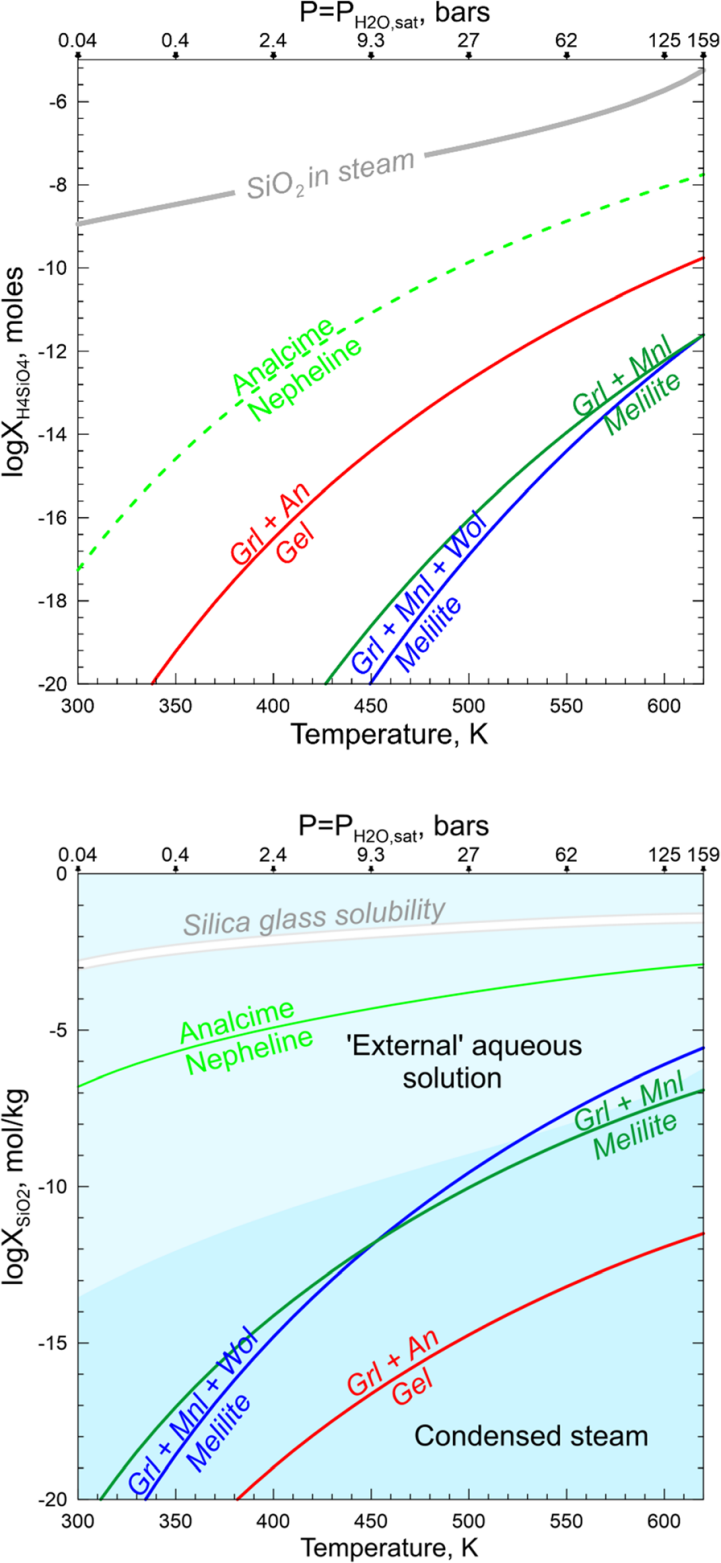
Phase boundaries of reactions involving either aqueous (top panel) or gaseous (lower panel) silica. The lack of analcime among alteration products places an upper limit on X_H4SiO4_ or X_SiO2_ in steam and aqueous solution, respectively. In the lower panel, the “condensed steam” field designates an aqueous solution formed by in situ condensation of steam as opposed to the “external” aqueous solution, which comes to a CAI from outside (matrix?) as a liquid

**Fig. 17:**
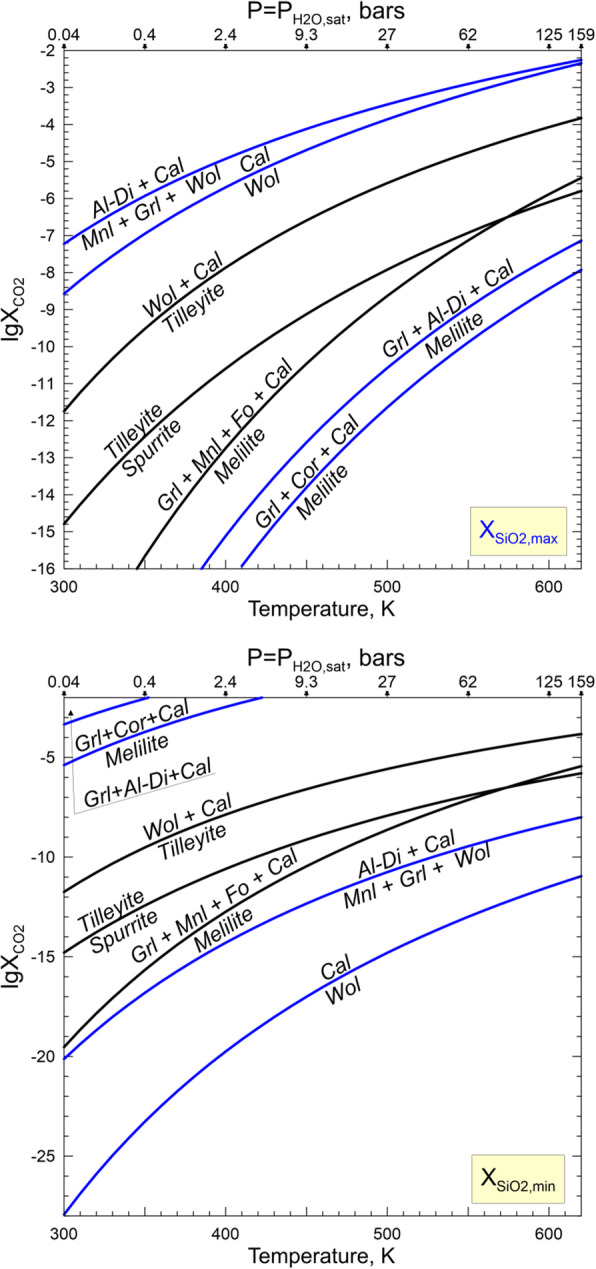
Phase boundaries of reactions involving CO_2_ (R16, R28, R29 - black lines) and aqueous SiO_2_ (R6, R7, R14, R30 - blue lines) at maximal (X_SiO2,max_ defined by R27 - top panel) and minimal (X_SiO2,min_ defined by R5 - bottom panel) values of SiO_2_ activity

**Fig. 18:**
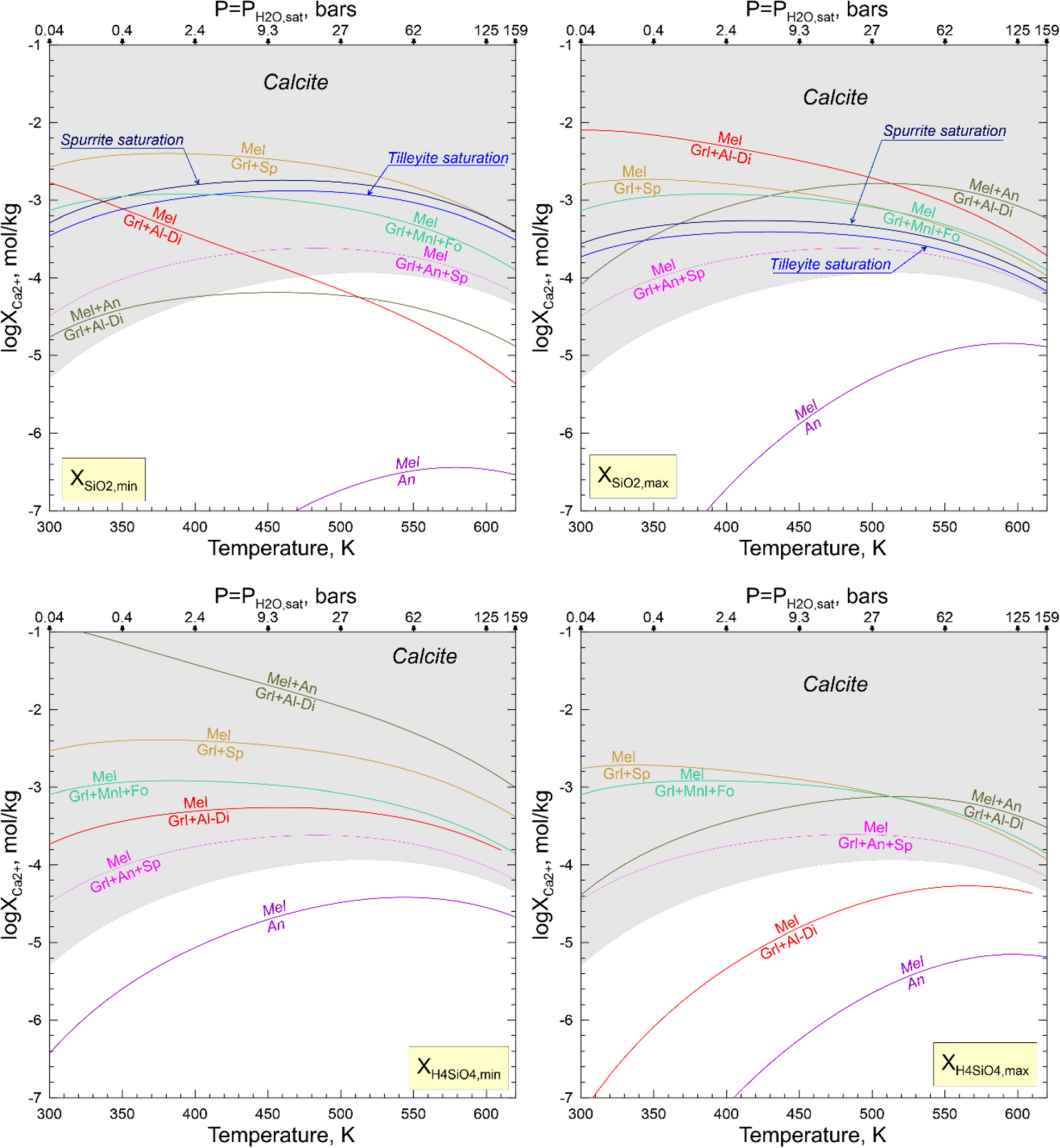
Phase boundaries of reactions involving addition of either aqueous SiO_2_ or gaseous H_4_SiO_4_ and removal of Ca in the form of dissolved Ca bicarbonate, Ca^2+^ + 2HCO_3_^−^, at X_CO2_ defined by R28 (wollastonite-calcite-tilleyite equilibrium) and different values of SiO_2_ activity and H_4_SiO_4_ fugacity defined by reactions R5 and R11 (Fig. 19). In each plot, the labeled curves of different colors separate the “primary” (above the curves) and “secondary” (below the curves) assemblages which correspond to the reactants and products of each reaction, respectively. The gray calcite saturation line separates two different phase assemblages containing silicates + calcite + aqueous solution above the line and silicates + aqueous solution below it. The tilleyite and spurrite saturation curves show conditions when a mineral starts to crystallize from an aqueous solution according to a reaction Ca^2+^ + HCO_3_^−^ + SiO_2_ → xCaO∙ySiO_2_∙zCO_2_ + CO_2(g)_ + H_2_O_(g)_

**Fig. 19:**
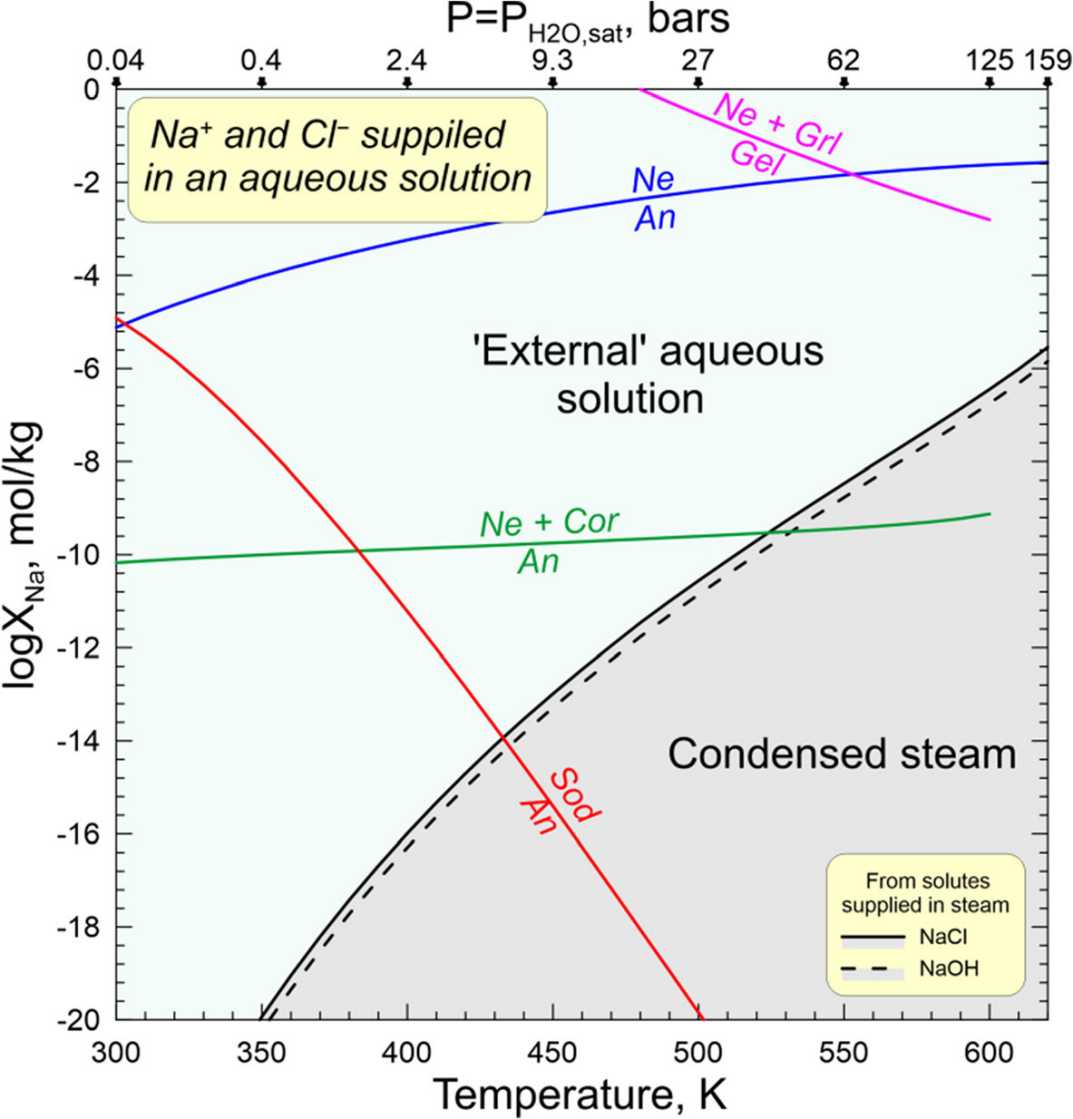
Phase boundaries of reactions R19, R20, R31 and R32 involving addition of Na^+^ and removal of Ca^2+^ at the X_CO2_ defined by R28, lowest X_Ca2+_ defined by R4 (melilite-anorthite equilibrium – top left panel of Fig. 20). The activity of aqueous SiO_2_ in reactions R31 and R32 is set at the upper limit of X_SiO2_ defined by R27. The “condensed steam” field designates an aqueous solution formed by in situ condensation of steam as opposed to the “external” aqueous solution, which comes to a CAI from outside (matrix?) as a liquid

To evaluate whether an observed alteration reaction can proceed via the gaseous phase only or requires a direct contact with an aqueous solution, one has to write appropriate reaction equations, calculate their intensive parameters, and compare them with each other and independent data if such exist. For example, reaction 7 can be described by the following equations:

29 $$ \mathrm{R}7g:2{\mathrm{Ca}}_2{\mathrm{Al}}_2{\mathrm{Si}\mathrm{O}}_{7\left(\mathrm{s}\right)}+{\mathrm{Ca}}_2{\mathrm{MgSi}}_2{\mathrm{O}}_{7\left(\mathrm{s}\right)}+2{\mathrm{H}}_4{\mathrm{Si}\mathrm{O}}_{4\left(\mathrm{g}\right)}+4{\mathrm{CO}}_{2\left(\mathrm{g}\right)}={\mathrm{Ca}}_3{\mathrm{Al}}_2{\mathrm{Si}}_3{\mathrm{O}}_{12\left(\mathrm{s}\right)}+{\mathrm{Ca}\mathrm{MgSi}}_2{\mathrm{O}}_{6\left(\mathrm{s}\right)}+{\mathrm{Ca}\mathrm{Al}}_2{\mathrm{Si}\mathrm{O}}_{6\left(\mathrm{s}\right)}+{{\mathrm{Ca}}^{2+}}_{\left(\mathrm{aq}\right)}+4{{{\mathrm{H}\mathrm{CO}}_3}^{-}}_{\left(\mathrm{aq}\right)}+{\mathrm{H}}_{2\left(\mathrm{g}\right)}+{\mathrm{H}}_2{\mathrm{O}}_{\left(\mathrm{aq}\right)} $$

30 $$ {\mathrm{lgK}}_{7g}=\lg \left[{\mathrm{X}}_{\mathrm{Di}}\right]+\lg \left[{\mathrm{X}}_{\mathrm{Cts}}\right]+\lg \left[{\mathrm{Ca}}^{2+}\right]+4\lg \left[{{\mathrm{H}\mathrm{CO}}_3}^{-}\right]+{\mathrm{lgP}}_{\mathrm{H}2}-2{\mathrm{lgP}}_{\mathrm{H}4\mathrm{SiO}4}-2\lg \left[{\mathrm{X}}_{\mathrm{Gel}}\right]-\lg \left[{\mathrm{X}}_{\overset{\circ }{A}\mathrm{k}}\right]-4{\mathrm{lgP}}_{\mathrm{CO}2} $$

31 $$ \mathrm{R}7 aq:2{\mathrm{Ca}}_2{\mathrm{Al}}_2{\mathrm{Si}\mathrm{O}}_{7\left(\mathrm{s}\right)}+{\mathrm{Ca}}_2{\mathrm{MgSi}}_2{\mathrm{O}}_{7\left(\mathrm{s}\right)}+2{\mathrm{Si}\mathrm{O}}_{2\left(\mathrm{aq}\right)}+4{\mathrm{CO}}_{2\left(\mathrm{g}\right)}+3{\mathrm{H}}_2{\mathrm{O}}_{\left(\mathrm{aq}\right)}={\mathrm{Ca}}_3{\mathrm{Al}}_2{\mathrm{Si}}_3{\mathrm{O}}_{12\left(\mathrm{s}\right)}+{\mathrm{Ca}\mathrm{MgSi}}_2{\mathrm{O}}_{6\left(\mathrm{s}\right)}+{\mathrm{Ca}\mathrm{Al}}_2{\mathrm{Si}\mathrm{O}}_{6\left(\mathrm{s}\right)}+{{\mathrm{Ca}}^{2+}}_{\left(\mathrm{aq}\right)}+4{{{\mathrm{H}\mathrm{CO}}_3}^{-}}_{\left(\mathrm{aq}\right)}+{\mathrm{H}}_{2\left(\mathrm{g}\right)} $$

32 $$ {\mathrm{lgK}}_{7 aq}=\lg \left[{\mathrm{X}}_{\mathrm{Di}}\right]+\lg \left[{\mathrm{X}}_{\mathrm{CTs}}\right]+2\lg \left[{\mathrm{Ca}}^{2+}\right]+4\lg \left[{{\mathrm{HCO}}_3}^{-}\right]-2\lg \left[{\mathrm{SiO}}_2\right]-2\lg \left[{\mathrm{X}}_{\mathrm{Gel}}\right]-2\lg \left[{\mathrm{X}}_{\overset{\circ }{A}\mathrm{k}}\right]-4{\mathrm{lgP}}_{\mathrm{CO}2} $$

where subscripts *s*, *g*, and *aq* denote solid, gaseous, and aqueous species, respectively; X_Di_, X_CTs_, X_Gel_, and X_Åk_ are mole fractions of diopside, Ca-Tschermakite, gehlenite, and åkermanite, respectively, in Al-diopside and melilte solid solutions; P_H2_, P_H4SiO4_, and P_CO2_ are partial pressures of H_2_, H_4_SiO_4_ and CO_2_ in the gaseous phase dominated by H_2_O; and [Ca^2+^], [HCO_3_^−^], and [SiO_2_] are activities of ions and neutral species in the aqueous solution. Note, that Si and C in the reaction R7*g* are supplied via gaseous phase, but the removal of Ca requires the presence of an aqueous solution; i.e., the H_2_O steam must condense to be able to dissolve the released Ca and drain it away from the reaction zone. The presence of calcite in some Allende CAIs (Fig. 12c) suggests that P_CO2_ in the gaseous phase was high enough to produce carbonic acid upon steam condensation, making the HCO_3_^−^ main anion of the aqueous solution. Similar reaction equations can be written for reactions 4−20. The results of modeling are shown in Figs. 16, 17, 18, and 19.

The reactions involving alteration of melilite (R5, R11, R12) by addition of either gaseous H_4_SiO_4_ or aqueous SiO_2_ (Fig. 16) can proceed forward at any temperature and pressure because the required H_4_SiO_4_ fugacities in steam and activities of SiO_2_ in aqueous solution are either much lower than or comparable with the solubility of SiO_2_ in the steam or the aqueous solution formed by its condensation.

Of special value here are the reactions describing possible alteration of nepheline to analcime by addition of SiO_2_ and ±H_2_O:

33 $$ \mathrm{R}27 aq:{\mathrm{NaAlSi}\mathrm{O}}_{4\left(\mathrm{s}\right)}+{\mathrm{SiO}}_{2\left(\mathrm{aq}\right)}+{\mathrm{H}}_2{\mathrm{O}}_{\left(\mathrm{aq}\right)}={\mathrm{NaAlSi}}_2{\mathrm{O}}_6\times {\mathrm{H}}_2{\mathrm{O}}_{\left(\mathrm{s}\right)} $$

34 $$ {\mathrm{lgK}}_{27 aq}=-\lg \left[{\mathrm{SiO}}_2\right] $$

35 $$ \mathrm{R}27g:{\mathrm{NaAlSi}\mathrm{O}}_{4\left(\mathrm{s}\right)}+{\mathrm{H}}_4{\mathrm{SiO}}_{4\left(\mathrm{g}\right)}={\mathrm{NaAlSi}}_2{\mathrm{O}}_6\times {\mathrm{H}}_2{\mathrm{O}}_{\left(\mathrm{s}\right)}+{\mathrm{H}}_2{\mathrm{O}}_{\left(\mathrm{g}\right)} $$

36 $$ {\mathrm{lgK}}_{27g}={\mathrm{lgP}}_{\mathrm{H}2\mathrm{O}}-{\mathrm{lgP}}_{\mathrm{H}4\mathrm{SiO}4}=-{\mathrm{lgX}}_{\mathrm{H}4\mathrm{SiO}4} $$

Experimental studies at T−P_H2O_ conditions identical to considered here (Wilkin and Barnes 1998) showed that analcime is a stable phase, which can be dissolved or replaced by other phases if activities of SiO_2_ and cations in the aqueous solution change. Therefore, the lack of analcime in the CV chondrites effectively places an upper limit on the H_4_SiO_4_ fugacity in the steam (Fig. 16, top panel) and SiO_2_ activity in aqueous solution (Fig. 16, bottom panel) implying that during CAIs’ alteration, neither steam nor aqueous solution was SiO_2_-saturated. Because the upper limit of aqueous SiO_2_ activity is 3−6 orders of magnitude higher than that in the aqueous solution made by in situ condensation of steam (Fig. 16, bottom panel), a solution with a higher concentration of SiO_2_, tentatively labeled as “external” aqueous solution, can be involved in CAI’s alteration. Such a solution must come to a CAI from outside (matrix?) as a liquid (Krot et al. 2001b; Ganino and Libourel 2017). In principle, it may be the same aqueous solution, which feeds the steam. Concentrations and ratios of solutes in such two solutions communicating with each other via steam would differ substantially due to different partition coefficients for different solutes. Another important chemical difference between these two solutions is that the “condensed steam” solution is expected to be free of Fe, Ca, and other elements, which are not efficiently transported by the steam. The lower limit on the H_4_SiO_4_ fugacity and SiO_2_ activity is set by reactions R11 and R5, respectively, which are widespread in the Allende coarse-grained igneous CAIs.

The phase equilibria involving loss or gain of CO_2_:

37 $$ \underset{\mathrm{calcite}}{\mathrm{R}28:\kern0.5em 3{\mathrm{Ca}\mathrm{CO}}_{3\left(\mathrm{s}\right)}}\underset{\mathrm{wollastonite}}{+2{\mathrm{Ca}\mathrm{SiO}}_{3\left(\mathrm{s}\right)}=}\underset{\mathrm{tilleyite}}{{\mathrm{Ca}}_5{\mathrm{Si}}_2{\mathrm{O}}_7{\left({\mathrm{CO}}_3\right)}_{2\left(\mathrm{s}\right)}+{\mathrm{CO}}_{2\left(\mathrm{g}\right)}} $$

38 $$ \underset{\mathrm{tilleyite}}{\mathrm{R}29:\kern0.5em {\mathrm{Ca}}_5{\mathrm{Si}}_2{\mathrm{O}}_7{\left({\mathrm{CO}}_3\right)}_{2\left(\mathrm{s}\right)}}\underset{\mathrm{spurrite}}{={\mathrm{Ca}}_5{\mathrm{Si}}_2{\mathrm{O}}_8{\left({\mathrm{CO}}_3\right)}_{\left(\mathrm{s}\right)}+{\mathrm{CO}}_{2\left(\mathrm{g}\right)}} $$

39 $$ \mathrm{R}30 aq:{\mathrm{CaCO}}_{3\left(\mathrm{s}\right)}+{\mathrm{SiO}}_{2\left(\mathrm{aq}\right)}={\mathrm{CaSiO}}_{3\left(\mathrm{s}\right)}+{\mathrm{CO}}_{2\left(\mathrm{g}\right)} $$

40 $$ \mathrm{R}30g:{\mathrm{CaCO}}_{3\left(\mathrm{s}\right)}+{\mathrm{H}}_4{\mathrm{SiO}}_{4\left(\mathrm{g}\right)}={\mathrm{CaSiO}}_{3\left(\mathrm{s}\right)}+{\mathrm{CO}}_{2\left(\mathrm{g}\right)}+2{\mathrm{H}}_2{\mathrm{O}}_{\left(\mathrm{g}\right)} $$

require rather low mole fraction of CO_2_ in the steam (Fig. 17), which seems to be reasonable for a C-bearing system such as carbonaceous chondrites. The occurrence of wollastonite, tylleyite, and calcite within the same CAI (*All-2*; Fig. 12a−c) gives a reasonable estimate of mole fractions (X_CO2_) and partial pressures (X_CO2_×P_H2O,sat_) of CO_2_ in the steam as well as activities of the HCO_3_^−^ ion in aqueous solution. At such conditions, the observed replacement of melilite by grossular + monticellite + forsterite (R16) can readily proceed, providing the released CaO reacts with CO_2_ to form calcite, regardless of whether an aqueous solution saturated in calcite is present or not.

Many alteration reactions require the addition of CO_2_±SiO_2_ accompanied by removal of Ca±SiO_2_. While both CO_2_ and SiO_2_ can be added or removed in either steam (bottom panels) or aqueous solution (top panels), the very low solubility of Ca in steam requires the presence of an aqueous solution, which would absorb the released Ca as either dissolved Ca bicarbonate, Ca^2+^ + 2HCO_3_^−^ (Fig. 18) or calcite (Fig. 17). If SiO_2_ is delivered in the steam, the latter must condense to form an aqueous solution, initially Ca-free, before alteration reactions can proceed. In such a case, the amount of aqueous solution is expected to be rather small, with its Ca absorption capacity being limited. The Ca concntration in the aqueous solution will affect the sequence of alteration reactions, with the alteration of gelenitic melilite to anorthite being the most sensitive to the Ca buildup. If such aqueous solution was not effectively drained before becoming calcite saturated, one would expect to see widespread calcite associated with altered silicates. While calcite does present at least in some altered Allende CAIs, it tends to occur in nodules hinting for effective removal of Ca^2+^ and HCO_3_^−^ with drained solution, which can accumulate in cavities and fill them with calcite and other CO_2_-bearing minerals upon evaporation of water to form nodules.

The formation of nepheline and sodalite (Fig. 19) requires an addition of Na and Cl and removal of Ca (R19, R20) and addition or removal of SiO_2_ (R31, R32):

41 $$ \mathrm{R}31 aq:2{\mathrm{Ca}}_2{\mathrm{Al}}_2{\mathrm{Si}\mathrm{O}}_{7\left(\mathrm{s}\right)}+3{\mathrm{Si}\mathrm{O}}_{2\left(\mathrm{aq}\right)}+2{{\mathrm{Na}}^{+}}_{\left(\mathrm{aq}\right)}={\mathrm{Ca}}_3{\mathrm{Al}}_2{\mathrm{Si}}_3{\mathrm{O}}_{12\left(\mathrm{s}\right)}+2{\mathrm{Na}\mathrm{AlSiO}}_{4\left(\mathrm{s}\right)}+{{{\mathrm{Ca}}^2}^{+}}_{\left(\mathrm{aq}\right)} $$

42 $$ \mathrm{R}32 aq:2{\mathrm{Ca}\mathrm{Al}}_2{\mathrm{Si}}_2{\mathrm{O}}_{8\left(\mathrm{s}\right)}+2{{\mathrm{Na}}^{+}}_{\left(\mathrm{aq}\right)}+2{\mathrm{CO}}_{2\left(\mathrm{g}\right)}+{\mathrm{H}}_2{\mathrm{O}}_{\left(\mathrm{aq}\right)}=2{\mathrm{Na}\mathrm{Al}\mathrm{SiO}}_{4\left(\mathrm{s}\right)}+{\mathrm{Al}}_2{\mathrm{O}}_{3\left(\mathrm{s}\right)}+2{{\mathrm{Ca}}^{2+}}_{\left(\mathrm{aq}\right)}+2{{{\mathrm{H}\mathrm{CO}}_3}^{-}}_{\left(\mathrm{aq}\right)}+2{\mathrm{Si}\mathrm{O}}_{2\left(\mathrm{aq}\right)} $$

Na, Cl, and SiO_2_ can be transported by either steam or aqueous solution, but the necessity to remove Ca requires the presence of an aqueous solution. As discussed above, the latter can be formed either by steam condensation or arrive from the matrix. The substitution of anorthite by sodalite at higher temperatures requires a solution with rather low Na (and Cl) concentrations that can be formed by the steam condensation. At lower temperatures, an “external” aqueous solution with higher Na and Cl concentration is required. Other reactions can proceed only in the presence of an “external” aqueous solution with high concentrations of Na and Cl. It is interesting to note that the presence of widespread Cl-bearing and Na-free wadalite in many altered Type B CAIs points to the decoupling of Na and Cl during at least the early stages of aqueous alteration. In this case, the Cl could be supplied by the steam containing HCl and NH_4_Cl but neither NaCl nor NaOH.

### Oxygen isotopic composition of metasomatic fluid on the CV chondrites parent asteroid

Most secondary minerals measured for oxygen isotopic composition in the Allende Type B and FoB CAIs (*TS-34*, *TS-31*, *AJEF*, *TS-21*, and *All-2*) replace primary melilite and to a lesser degree anorthite. These primary minerals are typically ^16^O-depleted relative to the minerals largely unaffected by secondary alteration – spinel, hibonite, forsterite, and most Al,Ti-diopside grains (very Ti-rich fassaite in *TS-34* is the only exception): ∆^17^O range from -5 to -1‰ vs. ~ -25 to -20‰, respectively. Larger variations in ∆^17^O (up to -10‰) are observed in anorthite of *TS-34* (Fig. 13a, b). In *TS-31*, *AJEF*, *TS-21*, and *All-2*, secondary minerals plot along mass-dependent fractionation line with ∆^17^O of ~ −3±2‰ and show a much smaller range of δ^18^O, from ~ 0 to ~ + 10‰, compared to the previously published data, from ~ −15 to + 20‰ (Fig. 13b, g) acquired without the use of proper mineral standards. Because metasomatic alteration of Allende CAIs occurred in the presence of aqueous solution/fluid and some minerals directly precipitated from it, we infer that ∆^17^O of the secondary minerals recorded ∆^17^O of the fluid. This conclusion is consistent with similar ∆^17^O (-1.5±1‰) of aqueously formed fayalite and magnetite in the oxidized CV3.1 chondrite Kaba (Doyle et al. 2015).

**Fig. 20:**
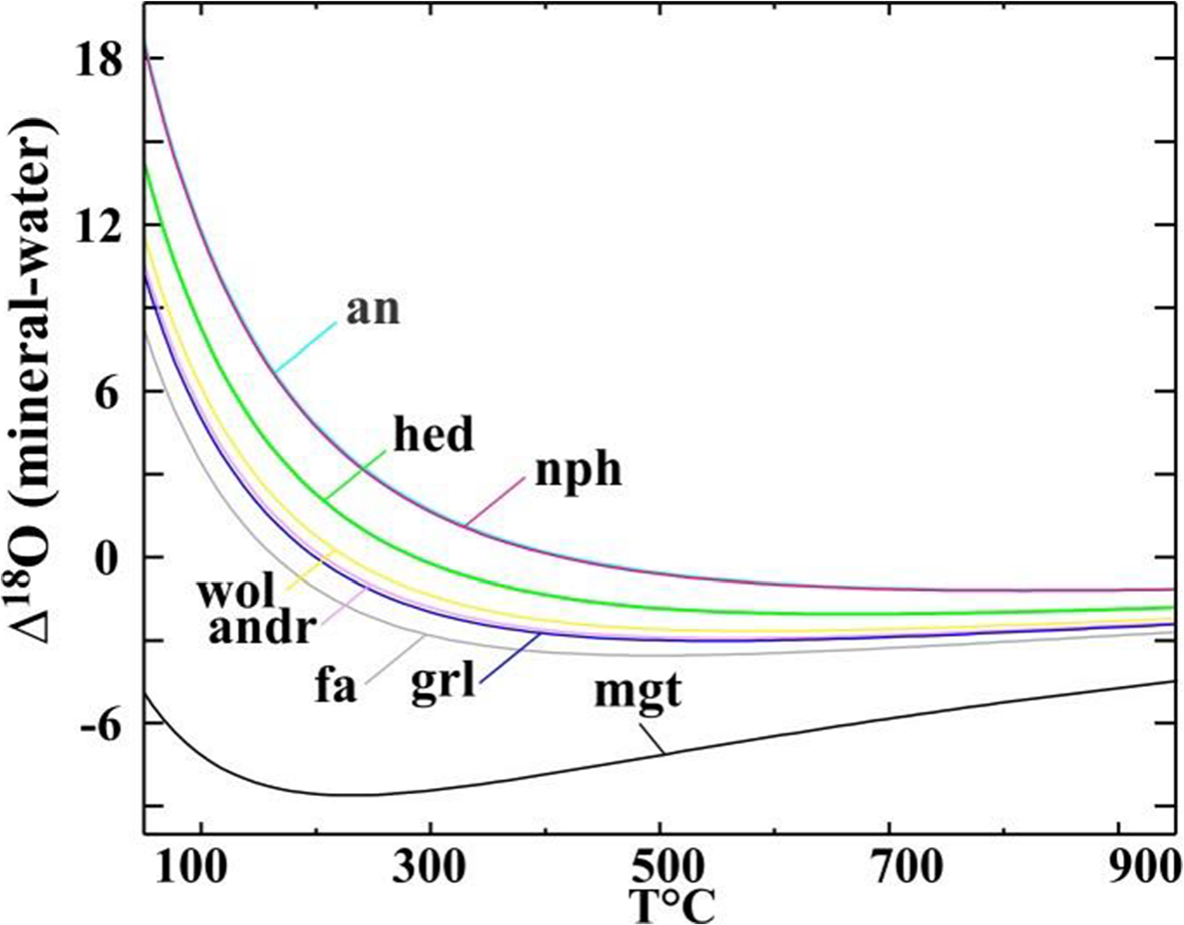
Calculated oxygen-isotope fractionations for anhydrous silicate-water and magnetite-water as a function of temperature (Zheng 1991, 1993)

**Fig. 21:**
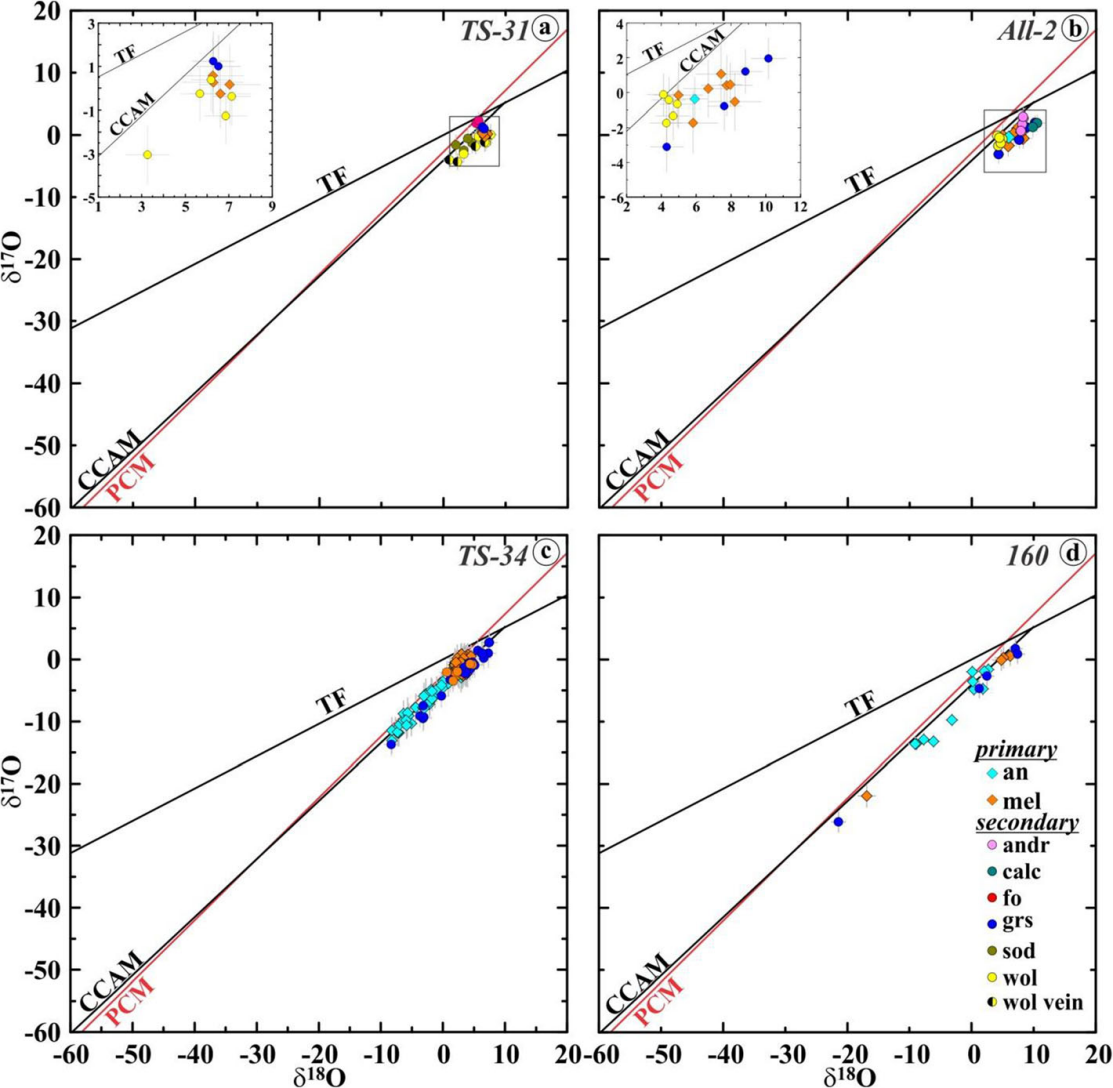
Three-isotope oxygen diagrams on primary melilite and anorthite, and secondary andradite, calcite, grossular, sodalite, and wollastonite in the Allende CAIs *TS-31* (B2), *All-2* (FoB), *TS-34* (B1), and *160* (C). Grossular and wollastonite (yellow circles) replace melilite and appear to have formed contemporaneously. Andradite, calcite, and wollastonite in veins (black-and-yellow circles) crosscutting the grossular±wollastonite-bearing assemblages precipitated directly from aqueous solutions. Primary melilite and anorthite are ^16^O-depleted relative to spinel, Al,Ti-diopside (except *TS-34*), and forsterite; melilite is more ^16^O-depleted than anorthite. Secondary minerals have similar compositions to those of anorthite and melilite. In *TS-31* and *All-2*, secondary minerals plot along mass-dependent fractionation line slightly below the TF line (∆^17^O ~ −3‰). Insets in “a” and “b” show O-isotope compositions of grossular and wollastonite that appear to have formed contemporaneously by replacing primary melilite; there is a small range of δ^18^O between these secondary minerals, 4 and 6‰ in *TS-31* and *All-2*, respectively. In *TS-34* and *160*, secondary grossular together with primary melilite and anorthite plot along CCAM line

Assuming that oxygen-isotope composition of the fluid was constant and that secondary minerals were in isotopic equilibrium with it, δ^18^O of the minerals with known water-mineral isotope fractionation can be used to constrain the temperature of alteration (Zheng 1991, 1993). Because O-isotope fractionation between mineral and water decreases with temperature increase (Fig. 20), the relatively small range of δ^18^O of the secondary minerals, ~ 10‰, which is much smaller than those between aqueously formed magnetite and carbonates in CI, CM, and CR chondrites (up to ~30‰; see Fig. 16 in Krot et al. 2015), is consistent with a relatively high temperature of the CV metasomatic alteration, > 200°C, compared to those of CIs, CRs, and CMs, < 100°C (Krot et al. 2015 and references therein). This is also consistent with a small range of δ^18^O, ~ 5‰, between aqueously formed magnetite and pure fayalite in the least metamorphosed CV chondrite Kaba, which apparently experienced lower temperature metasomatic alteration than Allende (Krot et al. 2018). We note, however, that our petrographic observations indicate that the chemical composition of the Allende fluid evolved with time and alteration was probably multistage (see above). Therefore it seems highly unlikely that the O-isotope composition of the fluid remained constant. As a result, the entire range of δ^18^O among secondary minerals measured, ~ 10‰, cannot be used to constrain the temperature of alteration of Allende CAIs: data for O-isotope compositions of secondary minerals that appear to have formed contemporaneously within an individual CAI could be more appropriate. Unfortunately, only a limited number of secondary minerals within individual CAIs have been measured with matrix-matched standards by now (Fig. 13c), and no special attention has been to whether the secondary minerals analyzed are cogenetic. To illustrate the latter point, in Fig. 21 a and b, we plotted O-isotope compositions of secondary minerals in two Allende CAIs, *TS-31* and *All-2*. Although the observed range of δ^18^O between grossular and wollastonite replacing melilite in these inclusions is rather limited, 4−6‰, the secondary minerals are apparently not in isotopic equilibrium (there are some differences in ∆^17^O) and may be not cogenetic. Therefore, these data cannot be used for estimating a temperature of metasomatic alteration as well.

Larger ranges of ∆^17^O are observed in secondary grossular and forsterite in the Type C CAIs *100* and *160* (from ~ −15 to ~ −1‰) and Type B1 CAI *TS-34* (from ~ −10 to ~ −1‰). In contrast to other CAIs measured, grossular and forsterite in these inclusions plot along CCAM line (Fig. 21c, d). The similar ranges of ∆^17^O and positions on a three-isotope oxygen diagram are observed for primary anorthite in these CAIs. Melilite is the most ^16^O-depleted primary phase in these inclusions (∆^17^O ~ −5 to ~ −1‰). We suggest that grossular and forsterite in *TS-34*, *100*, and *160* could have largely inherited oxygen isotopic compositions of the primary melilite and anorthite which experienced incomplete O-isotope exchange with the fluid phase (see Role of aqueous fluid–rock interaction in O-isotope exchange in primary CAI minerals). In the case of Type C CAIs, this might be due to alteration under relatively dry conditions (see Alteration of lacy melilite).

### Role of aqueous fluid–rock interaction in O-isotope exchange in primary CAI minerals

Oxygen isotopic composition of the CV aqueous fluid inferred from secondary minerals (∆^17^O ~ −3±2‰) is very similar to the most ^16^O-depleted compositions of primary melilite, anorthite, and very Ti-rich fassaite in the isotopically heterogeneous coarse-grained igneous CAIs from Allende studied (Fig. 13). Hibonite, spinel, forsterite, and most fassaite grains in these CAIs have ^16^O-rich, solar-like compositions. A similar O-isotope heterogeneity is observed in all types of igneous CAIs (CTA, B, FoB, and C) from metamorphosed CV chondrites, irrespective of mineral crystallization sequence and evaporation history (Fig. 13c, e, f). Because the vast majority of refractory inclusions in unmetamorphosed carbonaceous chondrites (CM2, CR2, CH3.0, CO3.0, and Acfer 094) have uniform solar-like oxygen isotopic compositions (∆^17^O ~ −24‰; Yurimoto et al. 2008; Makide et al. 2009; Ushikubo et al. 2017; Krot et al. 2020b), these observations may indicate that melilite and anorthite in Allende CAIs, experienced oxygen isotopic exchange with an aqueous fluid on the CV parent asteroid, whereas hibonite, spinel, Al,Ti-diopside, and forsterite retained their original compositions. Since ^16^O-rich and ^16^O-depleted gaseous reservoirs appear to have co-existed in the early solar nebula (e.g., Itoh and Yurimoto 2003; Simon et al. 2011, 2016, 2019; Krot et al. 2020b) and coarse-grained igneous CAIs experienced multiple melting events (Krot 2019 and references therein), this interpretation does not preclude O-isotope exchange during gas-melt interaction in the solar nebula of variable isotopic composition (e.g., Yurimoto et al. 1998; Kawasaki et al. 2018). To understand potential role of the CV aqueous fluid in O-isotope exchange, experimental data on oxygen self-diffusion in CAI minerals under high temperature and wet conditions are required.

### Aluminum-magnesium isotope systematics of secondary minerals in the Allende CAIs

Aluminum-magnesium isotope systematics of secondary minerals in the Allende CAIs is often used as evidence for a prolonged duration of the alteration that may have started in the solar nebula shortly after the formation of CAIs, continued during chondrule formation, and on the CV chondrite parent body (e.g., Ushikubo et al. 2007; Fagan et al. 2007). This interpretation is based on the occasional presence of resolvable ^26^Mg* in some secondary grossular and anorthite grains and its common absence in the vast majority of secondary minerals measured.

To test this interpretation, we measured Al-Mg isotope systematics in grossular grains replacing melilite of different chemical compositions (Fig. 14). Grossular in the nearly monomineralic veins crosscutting gehlenitic melilite mantle in the Type B1 CAI *TS-34* has a similar ^27^Al/^24^Mg ratio to those of the host melilite (6.5-9 vs. 5.8-10; up to 20 in melilite); all grossular grains measured show resolvable ^26^Mg*. In contrast, grossular associating with monticellite, forsterite, and Al-diopside, and replacing åkermanitic melilite (^27^Al/^24^Mg = 1.3-1.9) in *TS-34* and several other Type Bs has significantly higher ^27^Al/^24^Mg (30-100) ratio and lacks resolvable ^26^Mg* (0.2±2‰ for *TS-34*); the latter is indistinguishable from an average ^26^Mg* in the åkermanitic melilite (0.7±0.4‰). The high ^27^Al/^24^Mg ratio in these grossulars is probably due to Al-Mg fractionation between the grossular and other cogenetic secondary minerals: most magnesium is partioned into monticellite, forsterite, and Al-diopside. The upper limit on timing of this fractionation, and therefore, the timing of the metasomatic alteration is ~ 3.5 Ma. Based on these observations, we conclude that ^26^Mg* in grossular replacing both gehlenitic and åkermanitic melilite was largely inherited from melilite after nearly complete decay of ^26^Al, >3.5 Ma after crystallization of primary minerals of their host CAIs. The late-stage formation of grossular with and without resolvable ^26^Mg* is consistent with their similar O-isotope compositions. It is also consistent with ^53^Mn-^53^Cr ages of the metasomatically formed fayalite and kirschsteinite in the CV3 chondrites Asuka-881317 (${4.2}_{-0.7}^{+0.8}$ Ma, Doyle et al. 2015) and Vigarano (${3.2}_{-0.7}^{+0.8}$ Ma, MacPherson et al. 2017), respectively.

Ushikubo et al. (2007) reported ^26^Mg* in coarse-grained ragged anorthite surrounded by secondary grossular and monticellite in the Allende Type B2 CAI *2* that corresponds to (^26^Al/^27^Al)_0_ of ~ (1.2±0.2)×10^-5^. No resolvable ^26^Mg* was found in the grossular: (^26^Al/^27^Al)_0_ < 4.4×10^-7^. Largely based on the unusual morphology of the anorthite grains and their close association with grossular, Ushikubo et al. concluded that the anorthite is secondary, suggesting that alteration started 1.5 Ma after crystallization of CAIs with the canonical (^26^Al/^27^Al)_0_ and lasted for several Ma. Dr. T. Ushikubo kindly provided to us a polished section of this CAI. Our preliminary petrographic and mineralogical observations indicate that the ragged anorthite is in fact the primary igneous phase. The supporting arguments include: (*i*) the tripple junctions between ragged anorthite, Al,Ti-diopside, and unaltered åkermanitic melilite suggesting co-crystallization of these minerals; (*ii*) the presence of unaltered igneous inclusions of melilite and Al,Ti-diopside in some anorthite grains; (*iii*) the presence of secondary Na-rich melilite corroding edges of anorthite grains, as commonly observed in igneous anorthite in the Allende type Bs and FoBs (Fig. 7). We conclude that (1) secondary grossular, wollastonite, monticellite, forsterite, and Na-melilite around anorthite grains in the CAI *2* replace åkermanitic melilite, and are not genetically related to anorthite; and (2) the low initial ^26^Al/^27^Al ratio in ragged anorthite (Ushikubo et al. 2007) reflects the disturbance of its Al-Mg systematics during thermal metamorphism as often observed in anorthite of many Allende igneous CAIs (e.g., Kita et al. 2013; MacPherson et al. 2017). As a result, Al-Mg systematics of ragged anorthite in the Allende CAI *2* cannot be used for dating the CV metasomatic alteration.

## Conclusions

1. All major types of coarse-grained igneous CAIs (CTA, B1, B2, FoB, and C) from the oxidized CV3 chondrite Allende experienced metasomatic alteration. This alteration affected mainly melilite and to a lesser degree anorthite, and resulted in the formation of different secondary minerals, including adrianite, Al-diopside, andradite, anorthite, calcite, celsian, clintonite, corundum, dmisteinbergite, ferroan olivine, ferroan monticellite, ferroan Al-diopside, forsterite, grossular, heazlewoodite, hedenbergite, hutcheonite, kushiroite, margarite, monticellite, Na-melilite, nepheline, pentlandite, pyrrhotite, sodalite, spinel, tilleyite, wadalite, and wollastonite. The secondary mineral assemblages are mainly defined by chemical compositions of the primary melilite replaced:
  Gehlenitic melilite (Åk_<35_) in CTAs, mantles of B1s, and the outermost regions of other CAI types is mainly replaced by anorthite and grossular; clintonite, corundum, spinel, and Al-diopside are minor;
  Åkermanitic melilite (Åk_35-90_) in Type B2s, FoBs, cores of B1s, and CTA CAI *TS-2* is replaced by several mineral assemblages: grossular + monticellite + wollastonite, grossular + monticellite, and grossular + Al-diopside; forsterite, spinel, clintonite, and Na-melilite are minor;
  In type Cs, lacy melilite grains are pseudomorphically replaced by the grossular + forsterite + monticellite and grossular + Al-diopside mineral assemblages; Na-melilite is minor;
  Primary and secondary anorthites in the peripheral portions of Allende CAIs are replaced by nepheline, sodalite, and ferromagnesian olivine;
  All types of Allende CAIs studied contain voids and cracks filled by andradite, hedenbergite, wollastonite, ±sodalite, ±grossular, ±monticellite, ±tilleyite, and ±calcite.
2. Allende CAIs are surrounded by fine-grained matrix-like rims composed of the secondary lath-shaped ferroan olivine and abundant nepheline grains, and by a layer of salite-hedenbergite pyroxenes + andradite + wollastonite.
3. These observations and physico-chemical analysis of the inferred chemical reactions suggest that Allende CAIs experienced an open-system metasomatic alteration in situ in the presence of CO_2_- and H_2_O-bearing fluid on the CV chondrite parent asteroid at ~150-250 °C. During the alteration, Si, Na, Cl, K, Fe, S, and Ni were introduced, whereas Ca, Mg, and some Al were lost from the host inclusions; Ti and Ba were locally mobilized
4. In the Allende Type B CAIs, grossular associating with monticellite, forsterite, Al-diopside, and wollastonite, and replacing åkermanitic melilite has high ^27^Al/^24^Mg ratios (30−100) and shows no resolvable ^26^Mg*. The ^27^Al/^24^Mg ratios and ^26^Mg* in grossular replacing gehlenitic melilite are similar to those of the host melilite and plot along a regression line with ^26^Al/^27^Al ratio of ~5×10^−5^. We conclude that metasomatic alteration occurred after nearly complete decay of ^26^Al, >3 Ma after crystallization of CAIs with the canonical (^26^Al/^27^Al)_0_ of (5.25±0.02)×10^-5^. ^26^Mg* in grossular was inherited from primary melilite and provides no chronological significance.
5. Oxygen isotopic compositions of most secondary minerals in the Allende Type B CAIs measured in situ with the UH Cameca ims-1280 and matrix-matched standards plot along mass-dependent fractionation line with ∆^17^O of ~ −3±2‰ with δ^18^O ranging from ~ 0 to ~10‰. A smaller range of δ^18^O, ~5‰, is observed for cogenetic secondary minerals within individual CAIs. We conclude that ∆^17^O of ~ −3±2‰ corresponds to that of the aqueous fluid on the Allende parent asteroid. A small range of δ^18^O among the secondary minerals is consistent with high-temperature metasomatic alteration of ~ 150-250 °C.
6. Secondary grossular and forsterite in Type Cs and Type B1 CAI *TS-34* show a range of ∆^17^O, from ~ −15 to ~ −1±2‰; and on a three-isotope oxygen diagram plot along CCAM line. The similar variations in O-isotope compositions are observed for primary anorthite in these CAIs; primary melilite is generally more ^16^O-depleted (∆^17^O range from ~ −5 to ~ −1±2‰). Primary spinel, forsterite, and most Al,Ti-diopside grains have ^16^O-rich compositions (∆^17^O ~ −25±2‰). We suggest that oxygen isotopic compositions of grossular and forsterite in these CAIs were largely inherited from melilite and anorthite which may have experienced incomplete O-isotope exchange with a fluid.

## Supplementary Information

**Additional file 1: Figure EA1.** Backscattered electron images of the Allende Type B2 CAI #2 characterized by Ushikubo et al. 2007. Primary and secondary minerals are labeled by white and yellow symbols, respectively. The CAI consists of the irregularly-shaped (ragged) melilite (mel), anorthite (an), and Al,Ti-diopside (fas), all poikilitically enclosing spinel (sp) grains. Melilite, anorthite, and Al,Ti-diopside occasionally exhibit triple junctions (see “b”). Most anorthite grains contain exsolutions of submicron silicates. Some anorthite grains contain melt inclusions composed of Al,Ti-diopside and unaltered melilite (see “c”). Melilite is replaced to various degrees by grossular (grl), forsterite (fo), and monticellite (mnl). Anorthite is almost unaltered: only minor secondary Na-melilite (Na-mel), grossular, and kushiroite (kush) occur at the boundary with melilite. We conclude that ragged anorthite in CAI #2 is not a secondary phase; it is a primary mineral that crystallized from a CAI melt and largely escaped metasomatic alteration. Therefore, the Al-Mg isotope systematics of this anorthite cannot be used to date the metasomatic alteration. **Table EA1.** Representative electron microprobe analyses of secondary minerals in the Allende CAIs studied. **Table EA2.** Oxygen isotopic compositions of primary minerals in the Allende coarse-grained igneous CAIs measured by SIMS. **Table EA3.** Oxygen isotopic compositions of secondary minerals in the Allende coarse-grained igneous CAIs measured by SIMS.

## Data Availability

Please contact author (AK) for data requests.

## Footnotes

1 David Wark, Gerald Wasserburg, and Ian Hutcheon are deceased
