# Supplementary material for: Infiltration metasomatism of the Allende coarse-grained calcium-aluminum-rich inclusions
Source: Prog Earth Planet Sci. 2021 Nov 4;8(1):61. doi: 10.1186/s40645-021-00437-4 (PMC8568772; doi:10.1186/s40645-021-00437-4)
Supplement: Supplementary file 1 — Additional file 1: Figure EA1. Backscattered electron images of the Allende Type B2 CAI #2 characterized by Ushikubo et al. 2007. Primary and secondary minerals are labeled by white and yellow symbols, respectively. The CAI consists of the irregularly-shaped (ragged) melilite (mel), anorthite (an), and Al,Ti-diopside (fas), all poikilitically enclosing spinel (sp) grains. Melilite, anorthite, and Al,Ti-diopside occasionally exhibit triple junctions (see “b”). Most anorthite grains contain exsolutions of submicron silicates. Some anorthite grains contain melt inclusions composed of Al,Ti-diopside and unaltered melilite (see “c”). Melilite is replaced to various degrees by grossular (grl), forsterite (fo), and monticellite (mnl). Anorthite is almost unaltered: only minor secondary Na-melilite (Na-mel), grossular, and kushiroite (kush) occur at the boundary with melilite. We conclude that ragged anorthite in CAI #2 is not a secondary phase; it is a primary mineral that crystallized from a CAI melt and largely escaped metasomatic alteration. Therefore, the Al-Mg isotope systematics of this anorthite cannot be used to date the metasomatic alteration. Table EA1. Representative electron microprobe analyses of secondary minerals in the Allende CAIs studied. Table EA2. Oxygen isotopic compositions of primary minerals in the Allende coarse-grained igneous CAIs measured by SIMS. Table EA3. Oxygen isotopic compositions of secondary minerals in the Allende coarse-grained igneous CAIs measured by SIMS. [file 40645_2021_437_MOESM1_ESM.pdf]

**Supplementary Material**

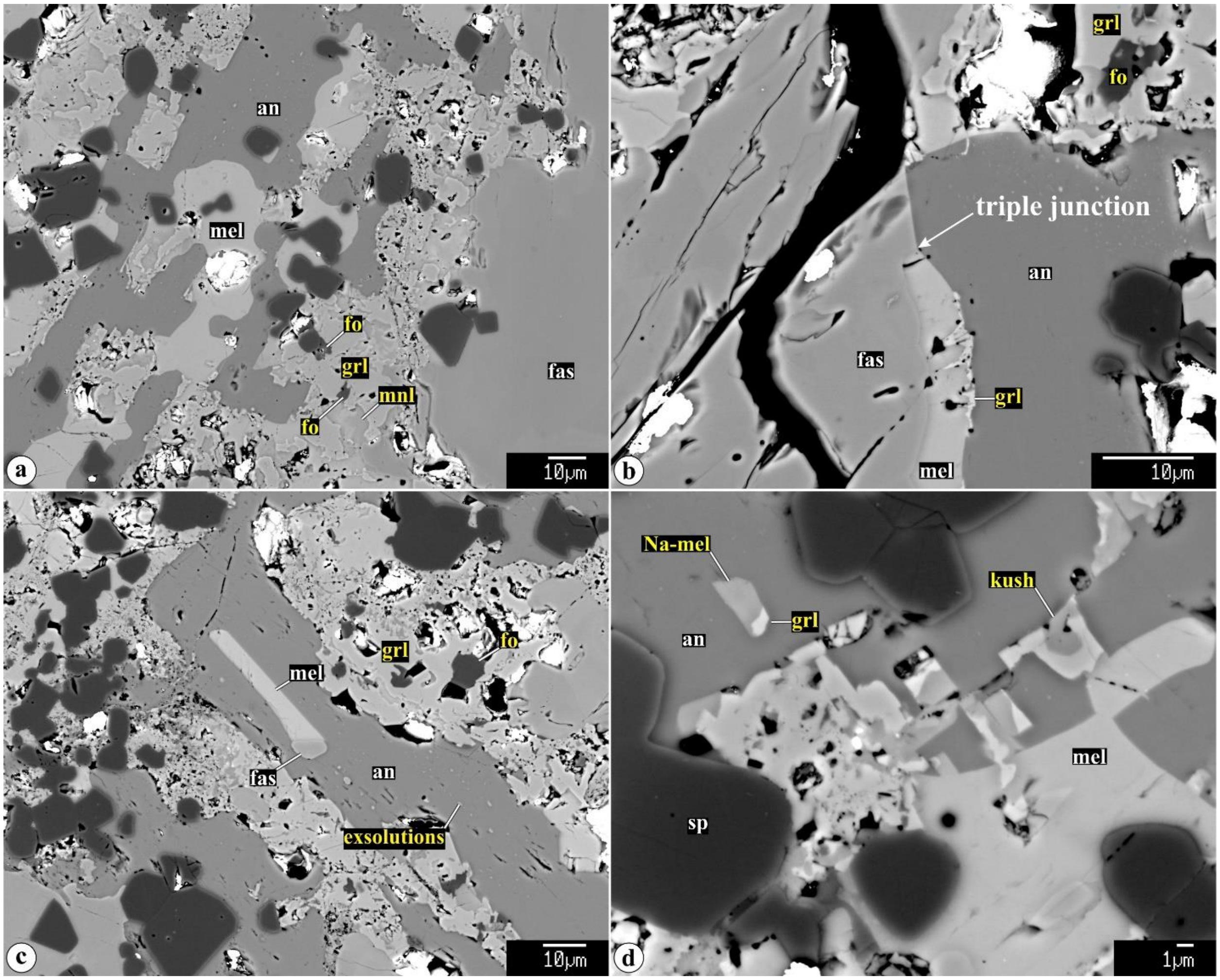


**Fig. EA1.** Backscattered electron images of the Allende Type B2 CAI *#2* characterized by Ushikubo et al. (2007). Primary and secondary minerals are labeled by white and yellow symbols, respectively. The CAI consists of the irregularly-shaped (ragged) melilite (mel), anorthite (an), and Al,Ti-diopside (fas), all poikilitically enclosing spinel (sp) grains. Melilite, anorthite, and Al,Ti-diopside occasionally exhibit triple junctions (see "b"). Most anorthite grains contain exsolutions of submicron silicates. Some anorthite grains contain melt inclusions composed of Al,Ti-diopside and unaltered melilite (see "c"). Melilite is replaced to various degrees by grossular (grl), forsterite (fo), and monticellite (mnl). Anorthite is almost unaltered: only minor secondary Na-melilite (Na-mel), grossular, and kushiroite (kush) occur at the boundary with melilite. We conclude that ragged anorthite in CAI *#2* is not a secondary phase; it is a primary mineral that crystallized from a CAI melt and largely escaped metasomatic alteration. Therefore, the Al-Mg isotope systematics of this anorthite cannot be used to date the metasomatic alteration.

Table EA1. Representative electron microprobe analyses of secondary minerals in the Allende CAIs studied.

| mineral | CAI | type | $SiO_2$ | $TiO_2$ | $Al_2O_3$ | $Cr_2O_3$ | FeO | MnO | MgO | CaO | $Na_2O$ | $K_2O$ | Cl | total |
|---|---|---|---|---|---|---|---|---|---|---|---|---|---|---|
| Al-diopside | *TS-23* | *B1* | 50.7 | 0.18 | 5.7 | n.d. | 0.65 | n.d. | 16.4 | 26.2 | n.d. | n.d. | n.a. | 99.7 |
| Al-diopside | *TS-34* | *B1* | 52.3 | 0.04 | 3.6 | n.d. | 0.69 | n.d. | 16.9 | 26.1 | n.d. | n.d. | n.a. | 99.6 |
| Al-diopside | *TS-31* | *B2* | 50.9 | n.d. | 7.1 | 0.16 | n.d. | n.d. | 15.6 | 26.2 | n.d. | n.d. | n.a. | 100.0 |
| Al-diopside | *All-2* | *FoB* | 53.0 | n.d. | 2.5 | n.d. | 0.07 | n.d. | 17.6 | 26.0 | n.d. | n.d. | n.a. | 99.2 |
| andradite | *All-2* | *FoB* | 35.6 | 0.05 | 0.33 | n.d. | 29.5 | n.d. | n.d. | 33.3 | n.d. | n.d. | n.a. | 98.8 |
| anorthite | *818-G* | *CTA* | 41.6 | 0.02 | 37.2 | n.d. | n.d. | n.d. | n.d. | 19.3 | n.d. | n.d. | n.a. | 98.1 |
| anorthite | *TS23* | *B1* | 41.6 | n.d. | 37.0 | n.d. | 0.07 | n.d. | 0.05 | 18.0 | 1.6 | n.d. | n.a. | 98.3 |
| anorthite | *AJEF* | B1/B2 | 40.7 | 0.072 | 39.0 | n.d. | 1.2 | n.d. | 0.938 | 17.6 | 0.19 | n.d. | n.a. | 99.76 |
| clintonite | *818-G* | *CTA* | 21.6 | 0.13 | 37.3 | 0.02 | 0.38 | n.d. | 20.9 | 13.7 | 0.21 | n.d. | n.a. | 94.2 |
| clintonite | *818-G* | *CTA* | 22.0 | 0.03 | 36.0 | n.d. | 1.8 | n.d. | 17.6 | 16.4 | 0.11 | n.d. | n.a. | 93.9 |
| clintonite | *TS-68* | *CTA* | 22.5 | n.d. | 36.3 | 0.04 | 0.52 | n.d. | 20.2 | 15.7 | 0.30 | n.d. | n.a. | 95.5 |
| corundum | *ALH-2* | *CTA/FTA* | n.d. | 0.16 | 98.2 | n.d. | 0.07 | n.d. | 0.02 | 0.29 | n.d. | n.d. | n.a. | 98.7 |
| forsterite | *AJEF* | B1/B2 | 41.2 | 0.03 | 1.2 | n.d. | n.d. | n.d. | 55.7 | 2.1 | n.d. | n.d. | n.a. | 100.2 |
| forsterite | *TS-23* | *B2* | 42.5 | n.d. | 0.54 | n.d. | 0.61 | n.d. | 56.5 | 1.5 | n.d. | n.d. | n.a. | 101.6 |
| ferroan grossular | *818-G* | *CTA* | 36 | 0.02 | 24.5 | 0.02 | 4.0 | 0.21 | 0.89 | 32.9 | n.d. | n.d. | n.a. | 98.8 |
| ferroan grossular | *TS-68* | *CTA* | 37.8 | n.d. | 20.0 | n.d. | 3.6 | n.d. | 1.8 | 36.1 | 0.02 | n.d. | n.a. | 99.3 |
| grossular, cgr | *TS-23* | *B1* | 38.9 | n.d. | 24.5 | n.d. | n.d. | n.d. | 0.23 | 37.2 | n.d. | n.d. | n.a. | 100.8 |
| grossular, cgr | *818-G* | *CTA* | 38.7 | 0.17 | 22.8 | n.d. | 1.3 | 0.05 | 0.20 | 35.9 | 0.02 | n.d. | n.a. | 99.0 |
| grossular, cgr | *TS-31* | *B2* | 39.4 | n.d. | 21.8 | n.d. | 0.024 | n.d. | 0.41 | 37.9 | n.d. | n.d. | n.a. | 99.51 |
| grossular, cgr | *All-2* | *FoB* | 39.1 | n.d. | 22.1 | n.d. | 0.15 | n.d. | 0.39 | 37.6 | n.d. | n.d. | n.a. | 99.3 |
| grossular, vein | *818-G* | *CTA* | 39.8 | 0.07 | 22.2 | n.d. | 0.52 | n.d. | 3.6 | 33.6 | n.d. | n.d. | n.a. | 99.8 |
| grossular, vein | *TS-23* | *B1* | 39.3 | 0.07 | 22.3 | n.d. | 0.66 | n.d. | 3.2 | 35.0 | n.d. | n.d. | n.a. | 100.5 |
| grossular, vein | *TS-23* | *B1* | 39.1 | n.d. | 23.0 | n.d. | 0.18 | n.d. | 2.3 | 35.5 | n.d. | n.d. | n.a. | 100.1 |
| grossular, vein | *TS-34* | *B1* | 39.1 | n.d. | 23.0 | n.d. | 0.18 | n.d. | 2.3 | 35.5 | n.d. | n.d. | n.a. | 100.1 |
| hedenbergite | *All-2* | *FoB* | 47.3 | n.d. | n.d. | n.d. | 27.4 | 0.28 | n.d. | 23.1 | n.d. | n.d. | n.a. | 98.1 |
| hutcheonite | *TS23* | *B1* | 25.1 | 19.8 | 14.2 | 0.30 | 1.2 | n.d. | 2.9 | 34.9 | 0.07 | n.d. | n.a. | 98.4 |
| hutcheonite | *Egg-3* | *B1* | 20.9 | 25.3 | 15.7 | n.d. | 0.70 | n.d. | 2.1 | 34.6 | n.d. | n.d. | n.a. | 99.3 |
| kushiroite | *TS-23* | *B1* | 28.0 | 0.09 | 47.6 | n.d. | n.d. | n.d. | 0.32 | 25.9 | n.d. | n.d. | n.a. | 101.8 |
| kushiroite | *TS-31* | *B2* | 28.9 | 0.07 | 40.6 | n.d. | 0.06 | n.d. | 1.1 | 27.4 | n.d. | n.d. | n.a. | 98.1 |

Table EA1 (cont.).

| mineral | CAI | type | $SiO_2$ | $TiO_2$ | $Al_2O_3$ | $Cr_2O_3$ | FeO | MnO | MgO | CaO | $Na_2O$ | $K_2O$ | Cl | total |
|---|---|---|---|---|---|---|---|---|---|---|---|---|---|---|
| tilleyite | *All-2* | *FoB* | 24.5 | n.d. | 0.03 | n.d. | n.d. | n.d. | n.d. | 57.2 | n.d. | n.d. | n.a. | 81.7 |
| monticellite | *All-2* | *FoB* | 38.4 | n.d. | n.d. | n.d. | 0.25 | n.d. | 25.6 | 35.7 | n.d. | n.d. | n.a. | 61.6 |
| ferroan monticellite | *All-2* | *FoB* | 35.4 | n.d. | 0.1 | n.d. | 12.2 | 0.28 | 16.5 | 33.2 | n.d. | n.d. | n.a. | 97.7 |
| ferroan monticellite | *818-G* | *CTA* | 35.3 | 0.09 | 2.3 | n.d. | 11.2 | 0.04 | 15.9 | 33.2 | n.d. | n.d. | n.a. | 98.0 |
| ferroan monticellite | *TS-68* | *CTA* | 35.0 | n.d. | 0.09 | 0.02 | 15.6 | 0.05 | 15.4 | 33.7 | n.d. | n.d. | n.a. | 99.9 |
| monticellite | *TS-23* | *B1* | 37.7 | n.d. | 0.26 | n.d. | 1.8 | n.d. | 24.7 | 36.0 | n.d. | n.d. | n.a. | 100.5 |
| monticellite, vein | *TS-34* | *B1* | 37.5 | n.d. | 0.61 | n.d. | 0.13 | n.d. | 24.7 | 36.2 | n.d. | n.d. | n.a. | 99.0 |
| monticellite | *TS-21* | *B2* | 37.6 | n.d. | 0.22 | n.d. | n.d. | n.d. | 25.2 | 36.3 | n.d. | n.d. | n.a. | 99.3 |
| monticellite | *TS-31* | *B2* | 37.3 | n.d. | n.d. | n.d. | 0.275 | n.d. | 25.4 | 36.4 | n.d. | n.d. | n.a. | 99.3 |
| Na-melilite | *TS-23* | *B1* | 32.2 | n.d. | 28.5 | n.d. | 0.08 | n.d. | 0.73 | 32.6 | 5.0 | n.d. | n.a. | 99.1 |
| Na-melilite | *TS-34* | *B1* | 31.1 | n.d. | 30.2 | n.d. | n.d. | n.d. | 0.43 | 34.0 | 4.3 | n.d. | n.a. | 100.1 |
| Na-melilite | *TS-31* | *B2* | 24.9 | n.d. | 34.1 | n.d. | n.d. | 0.07 | n.d. | 37.7 | 1.8 | n.d. | n.a. | 98.6 |
| Na-melilite | *All-2* | *FoB* | 27.4 | n.d. | 32.4 | n.d. | n.d. | n.d. | 0.99 | 37.7 | 1.7 | n.d. | n.a. | 100.2 |
| Na-melilite | *100* | *C* | 26.6 | n.d. | 33.0 | n.d. | 0.10 | 0.06 | 1.2 | 37.5 | 1.1 | n.d. | n.a. | 99.5 |
| nepheline | *TS-31* | *B2* | 43.2 | n.d. | 37.5 | n.d. | 0.17 | 0.05 | 0.03 | 2.6 | 15.4 | 1.7 | n.a. | 100.6 |
| nepheline | *AJEF* | *B1/B2* | 41.0 | n.d. | 36.0 | n.d. | 0.11 | 0.12 | n.d. | 2.7 | 16.9 | 1.9 | n.a. | 98.8 |
| ferroan olivine | *AJEF* | *B1/B2* | 39.0 | n.d. | 1.1 | n.d. | 13.8 | 0.25 | 43.9 | 0.21 | 0.44 | 0.04 | n.a. | 98.7 |
| ferroan olivine | *AJEF* | *B1/B2* | 36.6 | n.d. | 0.13 | n.d. | 26.3 | 0.52 | 34.2 | 0.24 | n.d. | n.d. | n.a. | 98.1 |
| ferroan olivine | *TS-31* | *B2* | 38.0 | n.d. | 0.20 | n.d. | 18.2 | 0.20 | 40.8 | 0.16 | 0.08 | n.d. | n.a. | 97.7 |
| wadalite/adrianite | *TS-23* | *B1* | 20.3 | n.d. | 26.4 | n.d. | n.d. | n.d. | 2.0 | 39.8 | 0.05 | n.d. | 10.2 | 98.8 |
| wadalite/adrianite | *TS-34* | *B1* | 20.9 | 0.23 | 21.0 | 0.03 | 0.12 | n.d. | 4.2 | 42.0 | 0.34 | 0.04 | 12.5 | 101.3 |
| wadalite/adrianite | *TS-21* | *B2* | 24.1 | 0.03 | 15.9 | n.d. | 0.16 | n.d. | 5.8 | 41.4 | 0.41 | n.d. | 12.2 | 100.0 |
| wollastonite | *TS-68* | *CTA* | 51.1 | n.d. | n.d. | n.d. | 0.74 | 0.05 | 0.05 | 47.7 | n.d. | n.d. | n.a. | 99.6 |
| wollastonite | *TS-23* | *B1* | 51.4 | n.d. | 0.42 | n.d. | n.d. | n.d. | 0.24 | 47.3 | n.d. | n.d. | n.a. | 99.4 |
| wollastonite | *TS-31* | *B2* | 50.5 | n.d. | n.d. | n.d. | n.d. | n.d. | 0.04 | 48.6 | n.d. | n.d. | n.a. | 99.1 |
| wollastonite | *All-2* | *FoB* | 51.3 | n.d. | 0.16 | n.d. | n.d. | n.d. | 0.09 | 48.2 | n.d. | n.d. | n.a. | 99.8 |

n.a. = not analyzed; n.d. = not detected.

Table EA2. Oxygen isotopic compositions of primary minerals in the Allend coarse-grained igneous CAIs measured by SIMS.

| CAI # | mineral | $\delta^{18}O$ | 2σ | $\delta^{17}O$ | 2σ | $\Delta^{17}O$ | 2σ |
|---|---|---|---|---|---|---|---|
| *TS-31* | mel | 7.0 | 1.4 | 0.2 | 1.8 | -3.5 | 2.0 |
| -"- | -"- | 6.3 | 1.4 | 0.6 | 1.7 | -2.7 | 1.9 |
| -"- | -"- | 6.6 | 1.4 | -0.3 | 1.6 | -3.7 | 1.7 |
| -"- | -"- | 6.3 | 1.4 | 0.3 | 1.6 | -3.0 | 1.7 |
| *AJEF* | an | -2.7 | 0.7 | -8.1 | 0.6 | -6.7 | 0.8 |
| -"- | -"- | 2.7 | 0.6 | -0.8 | 0.6 | -2.2 | 0.7 |
| -"- | -"- | -8.7 | 0.6 | -12.7 | 0.8 | -8.2 | 0.7 |
| -"- | fas | -47.4 | 0.7 | -47.0 | 0.6 | -22.4 | 0.8 |
| -"- | -"- | -46.5 | 0.6 | -48.3 | 0.7 | -24.1 | 0.7 |
| -"- | mel | 3.2 | 0.5 | -1.2 | 0.7 | -2.8 | 0.6 |
| -"- | -"- | 2.8 | 0.6 | -0.9 | 0.6 | -2.4 | 0.7 |
| -"- | -"- | 3.5 | 0.4 | -0.3 | 0.6 | -2.2 | 0.6 |
| -"- | -"- | 1.9 | 0.8 | -1.0 | 0.8 | -2.0 | 0.8 |
| -"- | -"- | 4.3 | 0.6 | -0.7 | 0.7 | -2.9 | 0.7 |
| -"- | sp | -45.2 | 0.6 | -47.3 | 0.6 | -23.7 | 0.7 |
| *TS-21* | an | 6.5 | 0.6 | 0.1 | 0.6 | -3.3 | 0.7 |
| -"- | -"- | 3.4 | 0.6 | -2.8 | 0.7 | -4.6 | 0.7 |
| -"- | -"- | 2.4 | 0.9 | -3.3 | 0.7 | -4.5 | 0.8 |
| -"- | -"- | 4.6 | 0.5 | -1.0 | 0.6 | -3.3 | 0.7 |
| -"- | fas | -46.4 | 0.7 | -48.1 | 0.6 | -24.0 | 0.7 |
| -"- | -"- | -44.8 | 0.5 | -47.4 | 0.5 | -24.1 | 0.6 |
| -"- | -"- | -45.1 | 0.8 | -49.1 | 0.6 | -25.6 | 0.7 |
| -"- | -"- | -46.2 | 0.8 | -48.9 | 0.6 | -24.9 | 0.7 |
| -"- | -"- | -43.9 | 0.4 | -47.3 | 0.4 | -24.5 | 0.5 |
| -"- | -"- | -38.7 | 0.6 | -43.7 | 0.6 | -23.6 | 0.7 |
| -"- | -"- | -41.5 | 0.5 | -46.4 | 0.5 | -24.8 | 0.6 |
| -"- | mel | 4.6 | 0.6 | -1.0 | 0.7 | -3.3 | 0.8 |
| -"- | -"- | 5.3 | 0.7 | -1.9 | 0.8 | -4.6 | 0.9 |
| -"- | -"- | 2.6 | 0.8 | -3.4 | 0.8 | -4.8 | 0.9 |
| -"- | -"- | 3.8 | 0.7 | -1.8 | 0.6 | -3.7 | 0.7 |
| -"- | sp | -44.5 | 0.6 | -47.2 | 0.6 | -24.0 | 0.7 |
| *All-2* | an | 5.9 | 0.7 | -0.4 | 1.2 | -3.4 | 1.3 |
| -"- | fas | -40.0 | 1.1 | -45.4 | 1.5 | -24.6 | 1.6 |
| -"- | -"- | -39.5 | 1.1 | -41.9 | 1.5 | -21.4 | 1.6 |
| -"- | -"- | -40.5 | 1.1 | -44.2 | 1.5 | -23.1 | 1.6 |
| -"- | -"- | -37.5 | 1.1 | -41.8 | 1.5 | -22.3 | 1.6 |
| -"- | -"- | -41.6 | 0.7 | -46.1 | 1.2 | -24.4 | 1.3 |
| -"- | -"- | -41.5 | 0.7 | -45.9 | 1.2 | -24.4 | 1.3 |
| -"- | -"- | -41.9 | 0.7 | -45.7 | 1.2 | -24.0 | 1.3 |
| -"- | -"- | -41.4 | 0.6 | -45.5 | 1.2 | -24.0 | 1.3 |

Table EA2 (cont.).

| CAI # | mineral | $\delta^{18}O$ | 2σ | $\delta^{17}O$ | 2σ | $\Delta^{17}O$ | 2σ |
|---|---|---|---|---|---|---|---|
| *All-2* | fas | -40.8 | 0.6 | -45.7 | 1.2 | -24.5 | 1.3 |
| -"- | fo | -43.0 | 0.7 | -46.6 | 1.2 | -24.2 | 1.3 |
| -"- | -"- | -43.2 | 0.7 | -46.6 | 1.2 | -24.2 | 1.3 |
| -"- | mel | 8.2 | 1.5 | -0.5 | 1.6 | -4.8 | 1.8 |
| -"- | -"- | 7.4 | 1.4 | 1.1 | 1.7 | -2.8 | 1.9 |
| -"- | -"- | 7.8 | 1.5 | 0.4 | 1.7 | -3.6 | 1.9 |
| -"- | -"- | 6.7 | 1.4 | 0.2 | 1.6 | -3.3 | 1.8 |
| -"- | -"- | 5.8 | 1.4 | -1.7 | 1.8 | -4.8 | 1.9 |
| -"- | -"- | 5.0 | 1.4 | -0.2 | 1.9 | -2.7 | 2.0 |
| -"- | -"- | 8.0 | 0.7 | 0.5 | 1.3 | -3.7 | 1.3 |
| -"- | sp | -42.2 | 1.0 | -45.3 | 1.7 | -23.4 | 1.8 |
| -"- | -"- | -47.8 | 1.2 | -47.8 | 1.5 | -23.0 | 1.6 |
| -"- | -"- | -42.4 | 1.0 | -46.8 | 1.7 | -24.8 | 1.8 |
| -"- | -"- | -41.4 | 0.6 | -45.5 | 1.2 | -24.0 | 1.3 |
| -"- | -"- | -44.3 | 0.7 | -47.2 | 1.2 | -24.2 | 1.3 |
| *100* | an | -17.6 | 0.8 | -23.5 | 0.6 | -14.3 | 0.7 |
| -"- | -"- | -13.4 | 0.8 | -20.1 | 0.7 | -13.1 | 0.8 |
| -"- | -"- | -15.7 | 0.8 | -21.2 | 0.7 | -13.0 | 0.8 |
| -"- | -"- | -12.8 | 0.8 | -19.6 | 0.7 | -13.0 | 0.8 |
| -"- | -"- | -14.0 | 0.8 | -20.0 | 0.7 | -12.7 | 0.8 |
| -"- | -"- | -12.9 | 0.8 | -18.8 | 0.7 | -12.1 | 0.8 |
| -"- | -"- | -6.2 | 0.8 | -11.6 | 0.6 | -8.4 | 0.8 |
| -"- | -"- | -4.4 | 0.8 | -10.5 | 0.7 | -8.2 | 0.8 |
| -"- | -"- | -3.7 | 0.8 | -9.8 | 0.6 | -7.9 | 0.7 |
| -"- | -"- | -3.6 | 0.8 | -9.4 | 0.7 | -7.5 | 0.8 |
| -"- | -"- | -0.8 | 0.8 | -6.5 | 0.7 | -6.1 | 0.8 |
| -"- | -"- | 0.9 | 0.8 | -5.3 | 0.7 | -5.8 | 0.8 |
| -"- | -"- | 2.4 | 0.8 | -3.1 | 0.7 | -4.4 | 0.8 |
| -"- | mel | 6.8 | 0.8 | 0.7 | 0.6 | -2.9 | 0.7 |
| -"- | -"- | 4.2 | 1.2 | 2.7 | 2.1 | 0.6 | 2.2 |
| -"- | -"- | 4.0 | 1.4 | -1.5 | 2.7 | -3.6 | 2.8 |
| -"- | -"- | 3.5 | 1.3 | 1.0 | 2.2 | -0.8 | 2.3 |
| -"- | -"- | 0.5 | 1.4 | -4.3 | 2.1 | -4.6 | 2.3 |
| -"- | -"- | -1.5 | 1.2 | -4.0 | 2.2 | -3.2 | 2.3 |
| -"- | -"- | 1.6 | 1.3 | -1.7 | 2.2 | -2.5 | 2.3 |
| -"- | sp | -42.8 | 0.8 | -48.0 | 0.7 | -25.7 | 0.8 |
| *160* | an | -6.1 | 0.8 | -13.2 | 0.6 | -10.0 | 0.8 |
| -"- | -"- | -8.9 | 0.8 | -13.6 | 0.6 | -9.0 | 0.7 |
| -"- | -"- | -7.7 | 0.8 | -12.9 | 0.6 | -8.9 | 0.7 |
| -"- | -"- | -9.1 | 0.8 | -13.6 | 0.6 | -8.9 | 0.8 |
| -"- | -"- | -9.1 | 0.4 | -13.6 | 0.3 | -8.9 | 0.4 |
| -"- | -"- | -3.1 | 0.8 | -9.8 | 0.6 | -8.1 | 0.7 |
| -"- | -"- | 1.8 | 0.8 | -4.8 | 0.6 | -5.7 | 0.7 |

Table EA2 (cont.).

| CAI # | mineral | $\delta^{18}O$ | 2σ | $\delta^{17}O$ | 2σ | $\Delta^{17}O$ | 2σ |
|---|---|---|---|---|---|---|---|
| *160* | an | 0.4 | 0.8 | -4.8 | 0.6 | -5.0 | 0.7 |
| -"- | -"- | 0.2 | 0.8 | -3.5 | 0.6 | -3.6 | 0.7 |
| -"- | -"- | 2.7 | 0.8 | -1.6 | 0.6 | -3.0 | 0.7 |
| -"- | -"- | 2.0 | 0.8 | -1.9 | 0.6 | -2.9 | 0.8 |
| -"- | -"- | 0.1 | 0.8 | -2.0 | 0.6 | -2.0 | 0.7 |
| -"- | fas | -38.3 | 0.8 | -44.1 | 0.6 | -24.1 | 0.8 |
| -"- | -"- | -39.2 | 0.8 | -43.5 | 0.6 | -23.1 | 0.7 |
| -"- | mel | 6.2 | 1.4 | 0.5 | 1.5 | -2.7 | 1.7 |
| -"- | -"- | 5.2 | 1.4 | 0.3 | 1.7 | -2.4 | 1.9 |
| -"- | -"- | -16.9 | 1.4 | -22.0 | 1.8 | -13.2 | 1.9 |
| -"- | -"- | 4.8 | 1.4 | -0.1 | 1.7 | -2.6 | 1.8 |
| -"- | sp | -47.0 | 0.8 | -48.6 | 0.6 | -24.1 | 0.8 |
| -"- | -"- | -46.2 | 0.8 | -47.8 | 0.6 | -23.8 | 0.7 |

Mineral abbreviations: an = anorthite; fas = fassaite; fo = forsterite; mel = melilite; sp = spinel.

Table EA3. Oxygen isotopic compositions of secondary minerals in the Allende coarse-grained igneous CAIs measured by SIMS.

| CAI # | mineral | $\delta^{18}O$ | 2σ | $\delta^{17}O$ | 2σ | $\Delta^{17}O$ | 2σ |
|---|---|---|---|---|---|---|---|
| *TS-34* | grl | 1.2 | 1.3 | -3.3 | 2.0 | -3.9 | 2.1 |
| -"- | -"- | -3.2 | 1.4 | -9.5 | 2.1 | -7.9 | 2.2 |
| -"- | -"- | 4.2 | 1.2 | -0.6 | 1.8 | -2.8 | 1.9 |
| -"- | -"- | -3.7 | 1.2 | -9.2 | 1.8 | -7.2 | 1.9 |
| -"- | -"- | -0.2 | 1.3 | -5.9 | 1.8 | -5.8 | 2.0 |
| -"- | -"- | 4.1 | 1.3 | -0.8 | 2.0 | -2.9 | 2.1 |
| -"- | -"- | 5.6 | 1.2 | 1.4 | 2.0 | -1.5 | 2.1 |
| -"- | -"- | 4.4 | 1.2 | -1.6 | 1.6 | -3.9 | 1.8 |
| -"- | -"- | 3.4 | 1.3 | -1.3 | 2.1 | -3.1 | 2.2 |
| -"- | -"- | 2.9 | 1.2 | -2.0 | 1.8 | -3.5 | 1.9 |
| -"- | -"- | 3.6 | 1.2 | -2.3 | 2.0 | -4.1 | 2.1 |
| -"- | -"- | 3.7 | 1.2 | -2.3 | 2.1 | -4.2 | 2.2 |
| -"- | -"- | -3.1 | 1.3 | -9.3 | 2.0 | -7.7 | 2.1 |
| -"- | -"- | -8.3 | 1.2 | -13.7 | 1.8 | -9.4 | 1.9 |
| -"- | -"- | 7.3 | 1.2 | 1.0 | 2.3 | -2.8 | 2.4 |
| -"- | -"- | 7.4 | 1.3 | 2.7 | 1.8 | -1.2 | 1.9 |
| -"- | -"- | 7.5 | 1.3 | 2.7 | 1.9 | -1.2 | 2.0 |
| -"- | -"- | 6.2 | 1.3 | 1.0 | 1.9 | -2.3 | 2.0 |
| -"- | -"- | 6.6 | 1.2 | 0.2 | 1.8 | -3.2 | 1.9 |
| -"- | -"- | 6.4 | 1.2 | 1.0 | 2.0 | -2.4 | 2.1 |
| -"- | -"- | 5.1 | 1.3 | -0.9 | 2.0 | -3.6 | 2.1 |
| -"- | -"- | 4.8 | 1.3 | -0.4 | 1.7 | -2.9 | 1.8 |
| -"- | -"- | -3.2 | 1.2 | -7.5 | 1.8 | -5.8 | 1.9 |
| -"- | Na-mel | 2.1 | 1.2 | -2.3 | 1.9 | -3.4 | 2.0 |
| -"- | -"- | 1.6 | 1.1 | -3.4 | 1.8 | -4.3 | 1.8 |
| -"- | -"- | 2.3 | 1.2 | -2.0 | 1.8 | -3.2 | 1.9 |
| -"- | -"- | 4.8 | 1.3 | -0.8 | 2.1 | -3.3 | 2.2 |
| -"- | -"- | 4.4 | 1.2 | -0.7 | 2.0 | -3.0 | 2.1 |
| -"- | -"- | 0.6 | 1.2 | -2.1 | 1.8 | -2.4 | 1.9 |
| *TS-31* | andr | 6.7 | 0.6 | 1.8 | 1.3 | 0.4 | 1.3 |
| -"- | -"- | 7.2 | 0.6 | 2.3 | 1.3 | 1.0 | 1.4 |
| -"- | grl | 7.4 | 1.0 | 1.2 | 1.3 | -2.0 | 1.4 |
| -"- | -"- | 8.1 | 1.0 | 0.9 | 1.4 | -2.1 | 1.5 |
| -"- | sod | 5.3 | 0.7 | -1.3 | 1.7 | -3.5 | 1.7 |
| -"- | -"- | 4.7 | 0.6 | -0.6 | 1.8 | -2.3 | 1.8 |
| -"- | -"- | 4.2 | 0.8 | -2.5 | 1.7 | -3.8 | 1.8 |
| -"- | -"- | 3.0 | 0.6 | -1.6 | 1.7 | -2.2 | 1.7 |
| -"- | wol | 4.3 | 1.0 | -3.1 | 1.4 | -4.6 | 1.5 |
| -"- | -"- | 1.9 | 1.0 | -4.2 | 1.3 | -4.4 | 1.4 |
| -"- | -"- | 7.2 | 0.9 | 0.3 | 1.5 | -2.6 | 1.6 |

Table EA3 (cont.).

| CAI # | mineral | $\delta^{18}O$ | $2\sigma$ | $\delta^{17}O$ | $2\sigma$ | $\Delta^{17}O$ | $2\sigma$ |
|---|---|---|---|---|---|---|---|
| *TS-31* | wol | 6.7 | 0.9 | -0.4 | 1.3 | -2.9 | 1.4 |
| -"- | -"- | 3.2 | 0.9 | -4.4 | 1.2 | -5.1 | 1.3 |
| -"- | -"- | 8.1 | 0.9 | -0.5 | 1.4 | -3.6 | 1.4 |
| -"- | -"- | 6.1 | 1.0 | -1.9 | 1.2 | -3.9 | 1.3 |
| -"- | -"- | 7.6 | 0.9 | -1.2 | 1.5 | -3.9 | 1.6 |
| -"- | -"- | 8.6 | 1.0 | 0.0 | 1.7 | -3.2 | 1.8 |
| -"- | -"- | 7.9 | 1.0 | -1.3 | 1.3 | -4.0 | 1.4 |
| *AJEF* | grl | 3.1 | 0.6 | -1.8 | 0.7 | -3.4 | 0.7 |
| -"- | -"- | 3.9 | 0.6 | -0.2 | 0.7 | -2.2 | 0.7 |
| -"- | -"- | 3.5 | 0.6 | -1.2 | 0.7 | -3.0 | 0.7 |
| -"- | -"- | 4.7 | 0.5 | -0.4 | 0.7 | -2.8 | 0.6 |
| *TS-21* | grl | 4.8 | 0.6 | -0.6 | 0.6 | -3.1 | 0.7 |
| -"- | -"- | 1.3 | 1.1 | -2.4 | 0.7 | -3.1 | 0.9 |
| -"- | -"- | 3.2 | 0.9 | 0.1 | 0.7 | -1.6 | 0.8 |
| -"- | -"- | 4.7 | 1.1 | -0.2 | 1.0 | -2.6 | 1.1 |
| -"- | -"- | 3.0 | 0.7 | -1.1 | 0.7 | -2.7 | 0.8 |
| -"- | mnl | 4.4 | 0.8 | -0.1 | 0.6 | -2.4 | 0.7 |
| -"- | sod | 1.1 | 0.4 | -2.2 | 1.4 | -2.8 | 1.4 |
| -"- | -"- | 1.1 | 0.4 | -1.8 | 1.4 | -2.3 | 1.4 |
| -"- | -"- | 1.9 | 0.5 | -1.7 | 1.8 | -2.7 | 1.8 |
| -"- | -"- | 2.9 | 0.6 | -1.9 | 1.8 | -3.4 | 1.8 |
| -"- | -"- | 1.9 | 0.7 | -1.4 | 1.8 | -2.3 | 1.8 |
| -"- | -"- | -0.3 | 0.7 | -3.5 | 1.8 | -3.3 | 1.8 |
| *All-2* | andr | 9.9 | 0.5 | 2.8 | 1.5 | -2.4 | 1.5 |
| -"- | -"- | 9.6 | 0.5 | 1.8 | 1.3 | -3.3 | 1.4 |
| -"- | -"- | 9.3 | 0.5 | 0.6 | 1.2 | -4.3 | 1.2 |
| -"- | -"- | 9.7 | 0.7 | 2.8 | 1.4 | -2.2 | 1.5 |
| -"- | cal | 10.3 | 2.1 | 2.1 | 1.3 | -3.8 | 1.7 |
| -"- | -"- | 9.2 | 2.1 | 1.5 | 1.5 | -3.9 | 1.8 |
| -"- | grl | 7.6 | 0.9 | -0.8 | 1.4 | -4.8 | 1.5 |
| -"- | -"- | 8.8 | 1.0 | 1.2 | 1.3 | -3.4 | 1.4 |
| -"- | -"- | 10.2 | 1.0 | 2.0 | 1.2 | -3.3 | 1.3 |
| -"- | -"- | 4.3 | 0.9 | -3.1 | 1.4 | -5.4 | 1.5 |
| -"- | wol | 5.0 | 1.0 | -0.3 | 1.2 | -2.9 | 1.3 |
| -"- | -"- | 5.8 | 1.0 | -0.8 | 1.3 | -3.8 | 1.4 |
| -"- | -"- | 5.1 | 0.9 | -1.9 | 1.4 | -4.6 | 1.5 |
| -"- | -"- | 5.7 | 1.0 | -1.4 | 1.4 | -4.3 | 1.5 |
| -"- | -"- | 5.4 | 1.0 | -0.5 | 1.3 | -3.3 | 1.4 |
| *100* | fo | -8.3 | 1.4 | -15.1 | 2.4 | -10.8 | 2.5 |
| -"- | -"- | -20.9 | 1.2 | -25.0 | 2.4 | -14.1 | 2.5 |
| -"- | grl | -19.6 | 0.8 | -23.5 | 1.7 | -13.3 | 1.8 |
| -"- | -"- | -1.1 | 0.7 | -3.7 | 2.0 | -3.1 | 2.0 |
| -"- | -"- | -20.9 | 1.0 | -26.6 | 1.9 | -15.7 | 2.0 |

Table EA3 (cont.).

| CAI # | mineral | $\delta^{18}O$ | 2σ | $\delta^{17}O$ | 2σ | $\Delta^{17}O$ | 2σ |
|---|---|---|---|---|---|---|---|
| *100* | grl | -5.7 | 0.7 | -9.3 | 1.6 | -6.3 | 1.6 |
| -"- | -"- | -7.9 | 0.8 | -11.5 | 1.9 | -7.4 | 2.0 |
| -"- | -"- | -6.2 | 0.9 | -10.7 | 2.2 | -7.5 | 2.2 |
| -"- | -"- | -1.9 | 1.0 | -7.3 | 2.0 | -6.3 | 2.1 |
| -"- | -"- | 7.4 | 0.9 | 1.7 | 2.2 | -2.2 | 2.2 |
| -"- | -"- | 5.3 | 1.0 | -1.7 | 2.1 | -4.5 | 2.2 |
| -"- | -"- | 3.5 | 0.9 | -1.2 | 1.9 | -3.1 | 1.9 |
| -"- | -"- | 3.6 | 0.7 | -1.4 | 2.0 | -3.3 | 2.0 |
| -"- | -"- | -10.3 | 0.9 | -13.9 | 1.8 | -8.5 | 1.9 |
| -"- | -"- | 3.4 | 0.9 | -1.8 | 2.0 | -3.6 | 2.1 |
| *160* | grl | 4.7 | 1.2 | -4.8 | 1.5 | -6.6 | 1.6 |
| -"- | -"- | 7.1 | 1.1 | 1.8 | 1.5 | -1.3 | 1.6 |
| -"- | -"- | 2.5 | 1.1 | -2.7 | 1.6 | -3.2 | 1.7 |
| -"- | -"- | -21.4 | 1.1 | -26.1 | 1.6 | -14.1 | 1.7 |
| -"- | -"- | 1.3 | 1.2 | -4.7 | 1.5 | -4.4 | 1.6 |
| -"- | -"- | 7.4 | 1.1 | 0.8 | 1.4 | -2.0 | 1.6 |
| -"- | -"- | -2.7 | 0.7 | -8.0 | 0.6 | -6.5 | 0.7 |
| -"- | -"- | -1.3 | 0.6 | -8.0 | 0.5 | -7.3 | 0.6 |
| -"- | -"- | -10.3 | 0.9 | -14.5 | 0.8 | -9.1 | 0.9 |
| -"- | -"- | 6.8 | 0.6 | 0.3 | 0.5 | -3.3 | 0.6 |
| -"- | -"- | 5.3 | 0.5 | -0.4 | 0.6 | -3.1 | 0.7 |
| -"- | -"- | 6.5 | 0.6 | -0.4 | 0.6 | -3.8 | 0.7 |
| -"- | -"- | 8.5 | 0.6 | 1.0 | 0.6 | -3.4 | 0.7 |

Mineral abbreviations: andr = andradite; cal = calcite; fo = forsterite; grl = grossular; mnl = monticellite; Na-mel = Na-melilite; sod = sodalite; wol = wollastonite.
